# Small-molecule inhibition of PTPRZ reduces tumor growth in a rat model of glioblastoma

**DOI:** 10.1038/srep20473

**Published:** 2016-02-09

**Authors:** Akihiro Fujikawa, Asako Nagahira, Hajime Sugawara, Kentaro Ishii, Seiichi Imajo, Masahito Matsumoto, Kazuya Kuboyama, Ryoko Suzuki, Naomi Tanga, Masanori Noda, Susumu Uchiyama, Toshiyuki Tomoo, Atsuto Ogata, Makoto Masumura, Masaharu Noda

**Affiliations:** 1 Division of Molecular Neurobiology, National Institute for Basic Biology (NIBB), 5-1 Higashiyama, Myodaiji-cho, Okazaki, Aichi, 444-8787, Japan; 2 Asubio Pharma Co., Ltd., 6-4-3 Minatojima-Minamimachi, Chuo-ku, Kobe, Hyogo, 650-0047, Japan; 3 Department of Bioorganization Research, Okazaki Institute for Integrative Bioscience, 5-1 Higashiyama, Myodaiji-cho, Okazaki, Aichi, 444-8787, Japan; 4 School of Life Science, The Graduate University for Advanced Studies (SOKENDAI), 5-1 Higashiyama, Myodaiji-cho, Okazaki, Aichi, 444-8787, Japan; 5 Department of Biotechnology, Graduate School of Engineering, Osaka University, 2-1 Yamadaoka, Suita, Osaka, 565-0871, Japan

## Abstract

Protein tyrosine phosphatase receptor-type Z (PTPRZ) is aberrantly over-expressed in glioblastoma and a causative factor for its malignancy. However, small molecules that selectively inhibit the catalytic activity of PTPRZ have not been discovered. We herein performed an *in vitro* screening of a chemical library, and identified SCB4380 as the first potent inhibitor for PTPRZ. The stoichiometric binding of SCB4380 to the catalytic pocket was demonstrated by biochemical and mass spectrometric analyses. We determined the crystal structure of the catalytic domain of PTPRZ, and the structural basis of the binding of SCB4380 elucidated by a molecular docking method was validated by site-directed mutagenesis studies. The intracellular delivery of SCB4380 by liposome carriers inhibited PTPRZ activity in C6 glioblastoma cells, and thereby suppressed their migration and proliferation *in vitro* and tumor growth in a rat allograft model. Therefore, selective inhibition of PTPRZ represents a promising approach for glioma therapy.

Gliomas are the most common primary brain tumor^1^. Gliomas of WHO grade I are curable with complete surgical resection and rarely evolve into higher-grade lesions. Grade II or III gliomas are invasive, and progress to higher-grade lesions with a poor outcome. Glioblastoma (or glioblastoma multiforme: Although “multiforme” is no longer part of the WHO designation, glioblastoma is still often abbreviated as “GBM”) is the highest grade glioma (grade IV). Glioblastomas, which arise *de novo* or progress from lower-grade gliomas, are known as the most malignant and common brain tumors because their tumor cells are highly proliferative and invade surrounding normal brain tissues. The median survival of patients diagnosed with glioblastoma is 14 months due to the lack of effective therapeutic options for patients with this lethal disease^2^.

Protein tyrosine phosphorylation controls many cellular functions in metazoans, and its dysregulation has been implicated in the etiology of various human cancers including gliomas^3^,^4^. Protein tyrosine kinases (PTKs) are well-known molecular targets for anticancer drugs. Protein tyrosine phosphatases (PTPs), which function as the enzymatic counterpart of PTKs, have generally been assumed to act as tumor suppressors because many PTKs have been identified as oncogenic proteins. Nevertheless, some PTPs including PTP1B and SHP1/2 have been positively implicated in oncogenesis and tumor progression^3^,^4^. Such PTPs may promote tumor cell growth by dephosphorylating some key components of the signaling pathways, thereby facilitating signal transduction. For example, PTP1B, a non-receptor type PTP, was shown to be overexpressed in breast tumors together with epidermal growth factor receptor (EGFR)-2 tyrosine kinase (also known as ERBB2, human HER2, or rat Neu)^5^. The genetic deletion of PTP1B resulted in resistance to lung metastasis of human breast cancer in the NDL2 (*Neu* deletion in extracellular domain 2) mouse model, which expresses an activated mutant form of Neu (refs 6 and 7). Moreover, MSI-1436, a specific inhibitor of PTP1B, is known to antagonize HER2 signaling, inhibits tumorigenesis in xenografts, and abrogates metastasis in the NDL2 transgenic mouse model^8^.

Regarding glioblastoma, EGFRvIII, an oncogenic mutant of EGFR, is commonly found in glioblastoma. EGFRvIII is a constitutively autophosphorylated receptor, which has been shown to facilitate the pathological development of glioblastoma^9^. The success of first-generation EGFR kinase inhibitors such as gefitinib in the treatment of lung cancer^10^ raised expectations that these kinase inhibitors may show activities against glioblastoma; however, this has remained largely unfulfilled. Drugs targeting EGFR including gefitinib have not improved the survival rates over those with standard therapies^11^. Previous studies reported that malignant gliomas strongly expressed PTPRZ (also called PTPζ or RPTPβ), one of the receptor-type PTPs (RPTPs) (refs 12 and 13). As more clinically relevant findings, it was recently reported that transcripts encoding PTPRZ were highly expressed in individual cells by single-cell RNA sequencing of primary human glioblastomas, and analyses of intratumoral heterogeneity annotated PTPRZ as the major positive regulator of cancer stemness *in vivo* (ref. 14). Anti-PTPRZ immunotoxin was previously shown to delay human U87 glioma formation in a xenograft model^15^. Furthermore, PTPRZ has been shown to play roles in cell migration and adhesion in variegated cells including neuronal, glial, and gastric mucosal cells^16^,^17^,^18^. *PTPRZ*-knockdown glioblastoma cells exhibited decreases in cell migration and proliferation *in vitro* and tumor size *in vivo* (refs 12, 13, 14, 15, 16, 17, 18, 19, 20). However, the significance of the intrinsic phosphatase activity of PTPRZ in cancer malignancy has not yet been elucidated.

In the present study, we determined whether the inhibition of PTPRZ was effective as an anticancer therapy. We herein identified the compound SCB4380 as the first potent PTPRZ inhibitor. We examined the molecular basis of the inhibition of PTPRZ by SCB4380, and subsequently demonstrated that the intracellular delivery of SCB4380 via liposome vehicles suppressed the tumor growth of glioblastoma cells by using rat C6 glioblastoma as a model.

## Results

### Involvement of the catalytic activity of PTPRZ in the high malignant phenotype of rat C6 glioblastoma

Three isoforms are generated by alternative splicing from a single *PTPRZ* gene; the two receptor-types PTPRZ-A and PTPRZ-B, and the secretory PTPRZ-S (also known as phosphacan)^21^. All three isoforms expressed in the normal brain have been shown to be highly glycosylated with chondroitin sulfate^21^, while a non-proteoglycan form of PTPRZ-B has also been detected in some peripheral tissues such as the gastric gland^16^. Rat C6 glioblastoma cells with a highly invasive phenotype are widely used as an experimental model for studying glioblastoma^22^. C6 cells mainly express the short receptor isoform PTPRZ-B (Supplementary Fig. S1A). The chondroitinase ABC treatment that removes chondroitin sulfate modifications had only a slight impact on the Western blot visualization of PTPRZ-B, indicating that PTPRZ-B expressed in C6 cells is the non-proteoglycan form (Supplementary Fig. S1B). We also found that a significant accumulation of the 75 kDa protein in C6 cells, the proteolytic whole intracellular fragment (Z-ICF) of PTPRZ-B (Supplementary Fig. S1B,C): this fragment is produced by the metalloproteinase- and presenilin (PS)/γ-secretase-mediated processing of PTPRZ-A/B (ref. 21).

**Figure 1:**
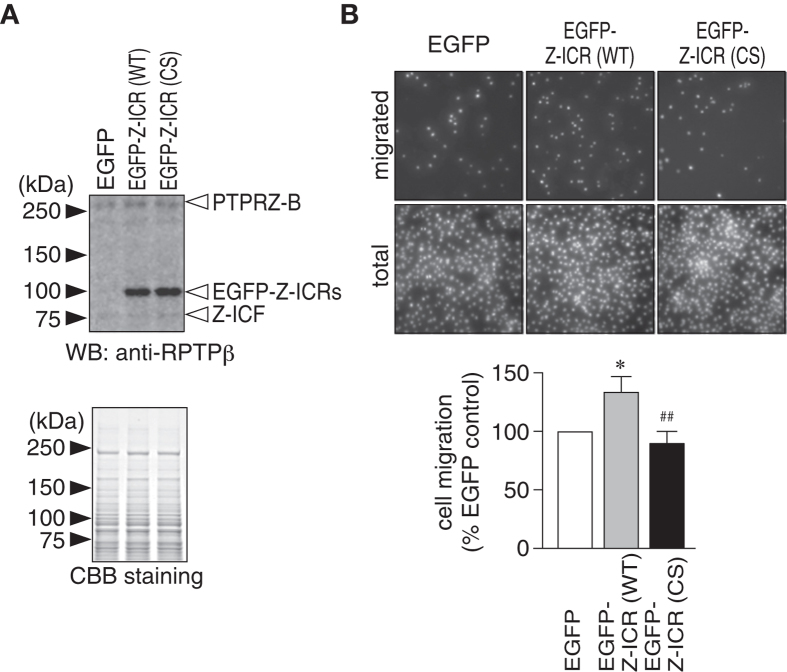
Enhanced migration of C6 glioblastoma cells by forced expression of the catalytically active intracellular fragment of PTPRZ. (**A**) Forced expression of the intracellular catalytic fragment of PTPRZ in rat C6 glioblastoma cells. C6 cells were electroporated with an EGFP-fused expression construct for a mock control, EGFP-fused PTPRZ-ICR (EGFP-Z-ICR), or its catalytically inactive CS mutant. Cell extracts were analyzed by Western blotting using anti-RPTPβ against the intracellular epitope (Supplementary Fig. S1A). The amount of protein loading was verified by coomassie brilliant blue (CBB) staining of the gel. (**B**) Boyden chamber assay. The cells described above were plated on the upper compartment of a laminin-coated chamber, and allowed to migrate to the lower side of the filter for 3 h. DAPI-stained nuclei were counted before (total) and after (migrated) the removal of cells remaining in the top chamber. The graph shows the quantification of the migrated cells to the lower side. Data are the mean ± S.E. (*n* = 5). *P < 0.05, significantly different from *EGFP*-transfected cells, ^##^*P* < 0.01, significantly different from *Z-ICR* (WT)-transfected cells by ANOVA with Fisher’s PLSD *post hoc* tests.

We investigated whether a large amount of Z-ICF contributed to the tumorigenic properties of C6 cells by overexpressing the entire intracellular region of PTPRZ (Z-ICR) fused with the green fluorescent protein (EGFP) in C6 cells. The overall expression of exogenous Z-ICRs was much higher than that of endogenous Z-ICF (Fig. 1A) under the condition in which approximately half of the cells showed fluorescent signals. Boyden chamber assays indicated that the overexpression of Z-ICR (WT) significantly enhanced epidermal growth factor (EGF)-induced cell migration, whereas that of the phosphatase-dead ICR mutant, Z-ICR (CS) did not (Fig. 1B). This result strongly suggested the causal role of the phosphatase activity of Z-ICF in glioblastoma malignancy.

**Figure 2:**
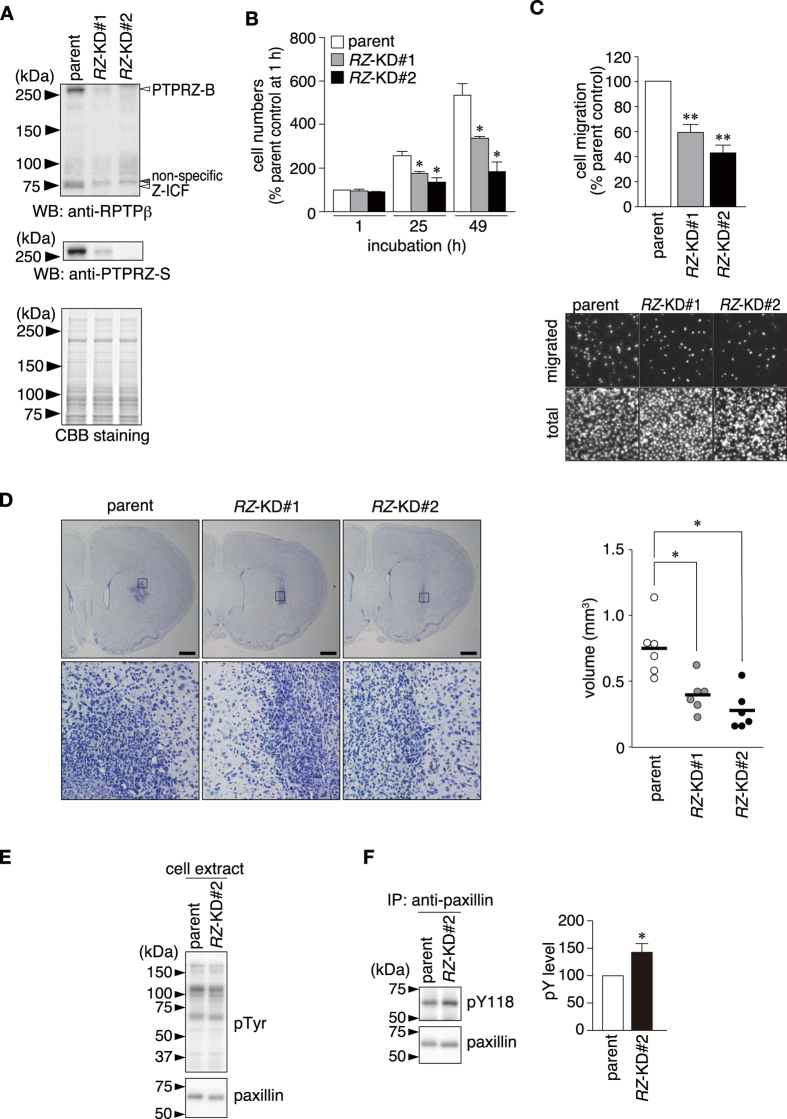
Involvement of PTPRZ catalytic activity in the high malignancy of C6 glioblastoma cells. (**A**) Establishment of stable *Ptprz-*knockdown C6 clones (#1 and #2). The amount of PTPRZ proteins in cell extracts was analyzed by Western blotting using anti-RPTPβ or anti-PTPRZ-S against the intracellular or extracellular epitope (Supplementary Fig. S1A). Sample loading was verified by CBB staining. (**B**) Cell proliferation assay. After plating equal numbers of cells, cell numbers were counted 1, 25, and 49 h after cultivation by hemacytometer. Cell numbers relative to the number of parental cells at 1 h are shown (the mean ± S.E., *n* = 4). *P < 0.05, significantly different from parent cells at the same time point by the Student’s *t*-test. (**C**) Boyden chamber assay. Data are the mean ± S.E. (*n* = 5). **P < 0.01 significantly different from the parent cells by the Student’s *t-*tests. (**D**) The effects of *Ptprz-*knockdown on tumor formation in a rat allograft model. Parental or *Ptprz*-knockdown C6 cells (5 × 10^5^ cells) were transplanted into the left striatum of Wistar rats, and the tumor was allowed to grow for 7 days. Representative coronal sections stained with hematoxylin are shown. The lower panels show the enlargement of the boxed area in the upper panels. Scale bars, 1 mm. The right graph shows the tumor volume determined by the serial sectioning of brains (*n* = 6 each). The horizontal bars indicate the average of each group. *P < 0.05, significantly different from that of parent cells by the Student’s *t*-test. (**E,F**) Increases in phosphorylation levels of paxillin at Tyr118 by *Ptprz* knockdown. The tyrosine phosphorylation of cellular proteins was examined by Western blotting using PY20, and paxillin expression was verified with anti-paxillin (**E**). The phosphorylation of paxillin at Tyr118 was analyzed by immunoprecipitation from the cell extracts with anti-paxillin, followed by Western blotting with anti-pY118-paxillin (**F**). Tyr118 phosphorylation levels were determined by densitometric analyses. Data are the mean ± S.E. (*n* = 3). *P < 0.05, significantly different from that of parent cells by the Student’s *t*-test.

On the other hand, the siRNA knockdown of *Ptprz* reduced the migration of C6 glioblastoma cells (Supplementary Fig. S2), as reported previously in other glioblastoma cell lines^12^,^13^,^14^,^15^,^16^,^17^,^18^,^19^,^20^. The stable knockdown of *Ptprz* in C6 cells also reduced cellular migration and proliferation, and these effects correlated with reductions in the expression of *Ptprz* (Fig. 2A–C). In order to determine the antitumor effects of *Ptprz* knockdown *in vivo*, we inoculated parent C6 cells or *Ptprz*-knockdown C6 cells into the brains of syngeneic Wistar rats, and evaluated their tumor sizes by hematoxylin staining. On the seventh day after tumor cell inoculation, the tumor size of *Ptprz*-knockdown cells appeared to be significantly smaller than that of parent glioblastoma cells (Fig. 2D).

We previously identified paxillin as a substrate for PTPRZ and phospho-Tyr118 of paxillin as the dephosphorylation site^23^. Paxillin is a major focal adhesion protein, and its phosphorylation at Tyr31 and Tyr118 has been shown to control cell migration activity in rat ascites hepatoma cells^24^. Phosphorylation levels at Tyr118 of paxillin in *Ptprz*-knockdown C6 cells were significantly higher than those from the parent C6 cells (Fig. 2E,F), indicating that the loss of PTPRZ affected the phosphorylation level of the substrate. However, notably, no significant differences were observed in the overall tyrosine phosphorylation pattern of cellular proteins and expression levels of paxillin (Fig. 2E). These results prompted us to determine whether pharmacological PTPRZ inhibition blocked the high malignant phenotypes of glioblastoma cells.

### Inhibitor screening

**Figure 3:**
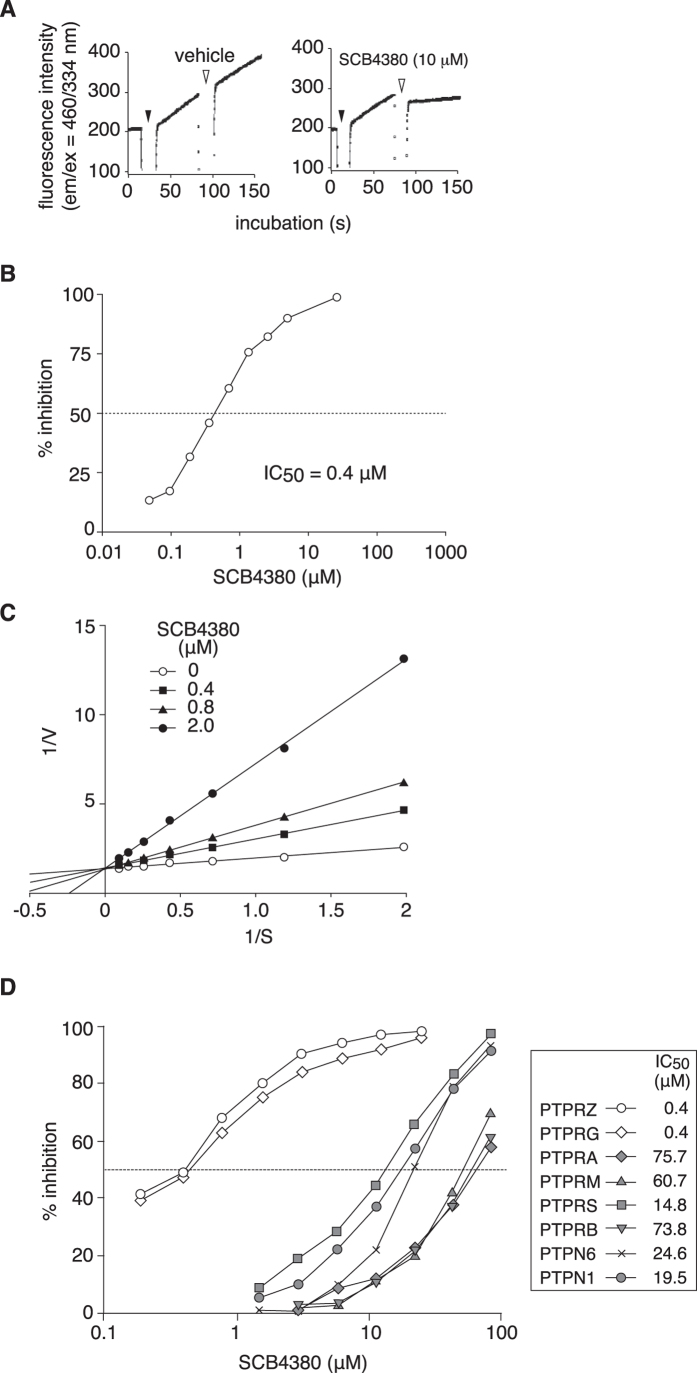
Enzymatic assay of PTPRZ and inhibition characteristics of SCB4380. (**A**) Time-course curve of the hydrolysis of a GIT1-derived pCAP peptide substrate (pCAP-GIT1_549–556_, 20 μM). Human PTPRZ-ICR (0.1 nM) was added to the substrate solution (closed arrowheads), and was followed by the addition of test compounds at the indicated concentrations (open arrowheads). The slope of the fluorescence signal during the 40 s before and after the addition of a compound was measured, and the inhibitory effects of each compound were determined by the ratio of the two. (**B**) Concentration-inhibition curves of SCB4380 for the hydrolysis of pCAP-GIT1_549–556_ (5 μM) by human PTPRZ. The IC_50_-value was 0.4 μM. (**C**) A Lineweaver-Burk plot analysis of PTPRZ inhibition by SCB4380. (**D**) Selectivity of SCB4380 for representative PTP members: human PTPRG (PTPγ, R5 RPTP subfamily member), human PTPRS (PTPσ, R2A subfamily), human PTPRM (PTPμ, R2B subfamily), mouse PTPRB (PTPβ, R3 subfamily), human PTPRA (PTPα, R4 subfamily), human PTPN1 (PTP1B, non-transmembrane, NT1 subfamily), and human PTPN6 (SHP1, NT2 subfamily). The inhibition assay was performed with 20 μM DiFMUP, a non-specific PTP substrate. The IC_50_-values are indicated in the inset. The results shown are representative ones of three separate experiments.

We took advantage of phosphotyrosine-mimic phosphocoumaryl amino propionic acid (pCAP) peptide technology developed as a simple method for probing PTP activity. The fluorogenic peptide probe pCAP-GIT1_549–556_ representing the typical PTPRZ substrate sequence^23^ was efficiently dephosphorylated by Z-ICR *in vitro* (Fig. 3A). After optimizing the assay conditions, we performed a screening of ~26,000 compounds in our original chemical library (Asubio Pharma) including small organic molecules and drug-oriented species (see Supplementary Table S1). We validated our hit compounds (a total of 32 initial hits of >40% inhibition at 10 μg/ml) with freshly purchased or prepared compounds, in which 23 compounds were eliminated due to their oxidizing potential or no reproducible inhibition. Among the remaining compounds, we found that SCB4380 (Trisodium 3-hydroxy-4-[(4-sulfonato-1-naphthyl)diazenyl]-2,7-naphthalenedisulfonate) exhibited the most potent and selective inhibition, with an IC_50_ of 0.4 μM against human Z-ICR-catalyzed hydrolysis (Fig. 3B). A Lineweaver-Burk plot analysis revealed typical competitive inhibition kinetics (Fig. 3C). We assessed the inhibitor selectivity of SCB4380 for a panel of PTP members, including PTPRG, another R5 family member, using a well-known non-specific PTP substrate, DiFMUP (6,8-difluoro-4-methylumbiliferyl phosphate). The results revealed that SCB4380 selectively and strongly inhibits PTPRZ and PTPRG (Fig. 3D).

### Structural basis of PTPRZ inhibition by SCB4380

**Figure 4:**
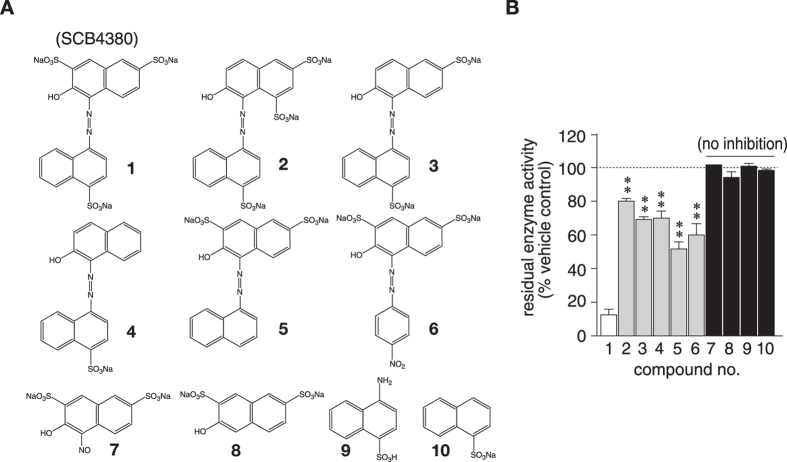
Inhibitory activities of SCB4380 and its analogues. (**A**) Structures of SCB4380 (compound 1) and its analogues (compounds 2 to 10). (**B**) Inhibitory effects of the compounds (10 μM each) on the hydrolysis of pCAP-GIT1_549–556_ (20 μM) by human PTPRZ-ICR (0.1 nM). Residual enzymatic activity is presented as a relative value to the DMSO control (mean ± S.E., *n* = 3). **P < 0.01 significantly different from that of SCB4380 by ANOVA and Scheffé’s *post hoc* tests. No significant effects were detected with compounds 7 to 10.

We noticed that SCB4380 is identical to Acid Red 27, a sulfonated azo dye (Fig. 4A). Acid Red 27 and its structural analogues are commercially available and widely used in food and cosmetic industries. The inhibition potency of these commercially available analogues was markedly lower than that of SCB4380, or even negligible (Fig. 4B). The high residual enzymatic activity of Z-ICR by compounds 2 to 4 suggested the pivotal role of the sulfonate group at the 2-position of the disulfonated naphthalene moiety of SCB4380 for inhibition. The removal of the sulfonate from the 4-position of another naphthalene (compound 5) or substitution of the naphthalene unit by a nitro benzene (compound 6) also markedly abolished inhibitory activity. Furthermore, the disulfonated naphthalene moiety (compounds 7 and 8) or another naphthalene moiety (compounds 9 and 10) by itself exhibited no inhibition, indicating that the whole chemical structure of SCB4380 was required for optimal inhibition.

**Figure 5:**
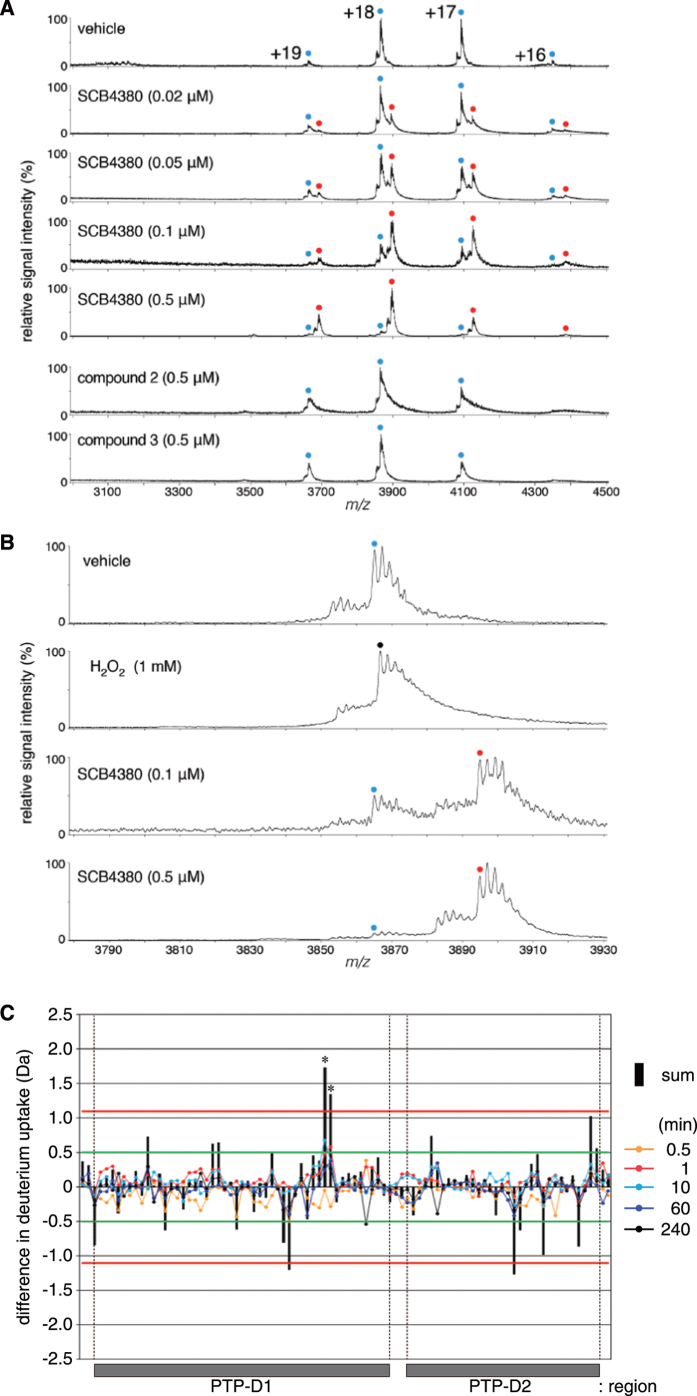
One-to-one binding of SCB4380 to the catalytic D1 of PTPRZ-ICR. (**A**) MS spectra of PTPRZ-ICR in the presence of SCB4380. PTPRZ-ICR was mixed with the indicated compounds, and their binding to PTPRZ-ICR was monitored by MS under non-denaturing conditions. Peaks corresponding to PTPRZ-ICR and PTPRZ-ICR with SCB4380 (1:1 complex) are indicated by blue and red dots (with charge states), respectively. No detectable binding was observed with compound 2 or 3. (**B**) MS spectra of PTPRZ-ICR after incubation with SCB4380 or hydrogen peroxide solution. The smallest peaks corresponding to native and oxidized PTPRZ-ICR, and the complex of PTPRZ-ICR with SCB4380 are indicated by blue (69552.5 ± 2.9 Da), black (69589.7 ± 6.6 Da), and red (70092.1 ± 1.4 Da) dots, respectively. The masses of the three forms were estimated from their parent ion peaks ranging from 3500 to 4500 *m*/*z*, and shown as the mean ± S.D. of triplicate measurements. The concentrations of PTPRZ-ICR proteins used for the assay were estimated to be approximately 0.2 to 0.3 μM by SDS-PAGE followed by CBB staining. (**C**) Hydrogen/deuterium (H/D) exchange protection of PTPRZ-ICR by SCB4380. The X-axis represents the sequential ordering of the ninety peptides derived from the PTPRZ-ICR sequence, while the Y-axis shows the mass difference between the presence and absence of SCB4380. The line plots show the average mass difference values at the indicated time points for each peptide, and black vertical bars show the sum of their mass differences for each peptide. The criteria of significant differences in the H/D exchange experiments as described previously^47^: Differences were considered to be significant with 98% confidence, when the individual H/D exchange difference was greater than 0.5 Da for at least one labeling time point (the green horizontal lines) and also the total H/D exchange difference summed from five time points was greater than 1.1 Da (the red horizontal lines). By this criteria, two overlapping peptides, 1913–1942 and 1914–1943 regions, indicated by asterisk exhibited significant changes in H/D exchange kinetics upon SCB4380 binding. H/D exchange MS data for the peptides analyzed are shown in Supplementary Fig. S3B.

**Figure 6:**
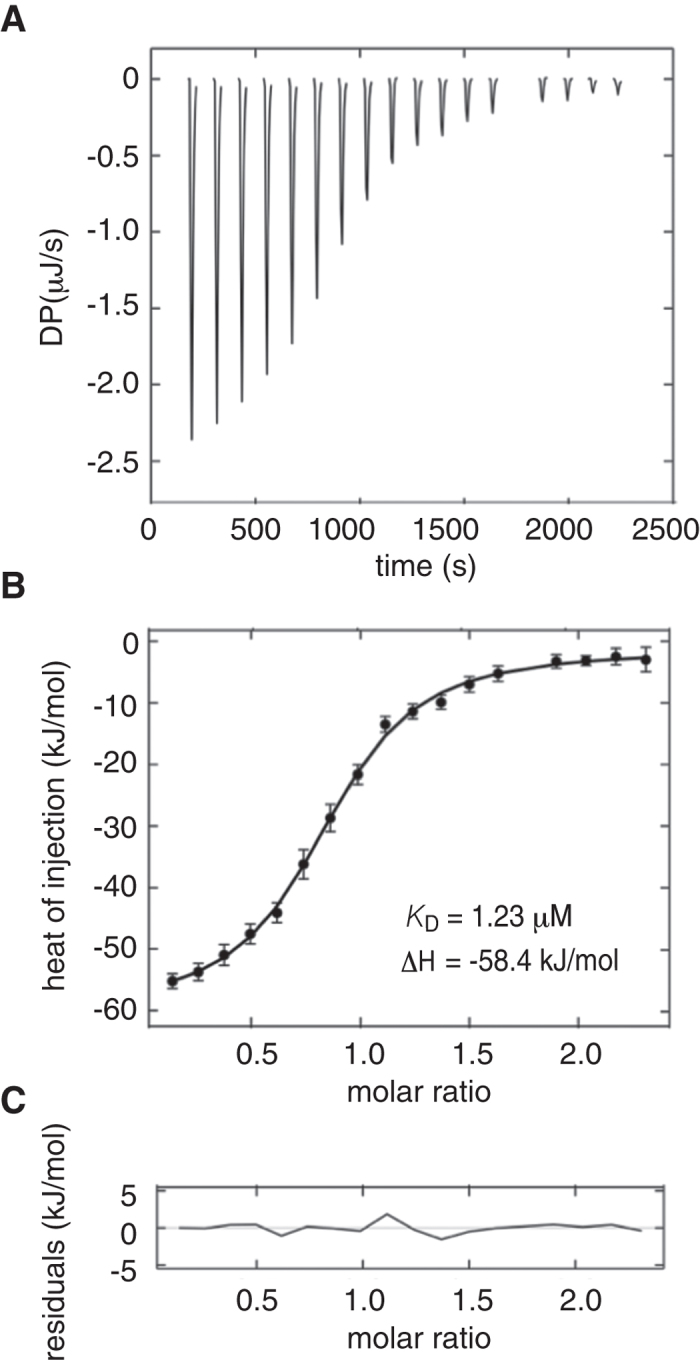
Isothermal titration calorimetry of SCB4380 into PTPRZ-ICR. (**A**–**C)** ASCB4380 at a concentration of 250 μM was titrated into 20.9 μM of PTPRZ-ICR. Degrees of polymerization (DP) of the experimental thermogram after automated thermogram processing using NITPIC (**A**). Titration isotherm (with error bars). The isotherm was fit to a one-to-one binding model (solid line) using the program SEDPHAT (ref. 48) with a 1:1 binding model (**B**). The single dissociation constant and enthalpy change are indicated in the inset. Residuals calculated by subtracting fit values from observed values (**C**).

To determine the binding properties of SCB4380 to Z-ICR, we took advantage of mass spectrometry (MS) under non-denaturing conditions (a technique often referred to as native MS), which is a powerful technique for studying noncovalent molecular interactions^25^). The MS analyses revealed that SCB4380 bound to Z-ICR in a one-to-one stoichiometry even in the presence of an excess amount of SCB4380 (0.5 μM), whereas compound 2 or compound 3 exhibited no binding activity (Fig. 5A). These results demonstrated the specific binding of SCB4380, and excluded the possibility that SCB4380 induced protein aggregation, and secondarily resulted in enzyme inhibition. Isothermal titration calorimetry experiments showed that the titration of SCB4380 into Z-ICR solution generated exothermic heat. The non-linear fit of the results obtained revealed 1:1 binding with *K*_D_ of 1.23 μM (Fig. 6), which was reasonably consistent with the IC_50_ of 0.4 μM.

Oxidizing agents such as hydrogen peroxide are non-selective PTP inhibitors because they irreversibly oxidize the catalytic cysteine residue that exists as a thiolate anion, a highly reactive nucleophile, to sulfinic acid or sulfonic acid^26^. When the enzyme solution was incubated in the presence of hydrogen peroxide, the molecular mass of Z-ICR (indicated by blue circle in Fig. 5B) shifted by approximately +37 Da (black circle), which corresponded to a change from thiolate to a protonated sulfinic acid anion. However, SCB4380 did not affect the molecular mass of Z-ICR itself: the +537 Da shift corresponded to the binding of a protonated SCB4380 (red circle), clearly eliminating the possibility of oxidation-dependent inactivation.

Most RPTPs including PTPRZ contain two tandem PTP domains in the intracellular region; the catalytic activity is retained in the membrane-proximal domain (D1), but not in the membrane-distal D2 domain^27^. In order to determine the SCB4380 binding site on Z-ICR, we performed hydrogen/deuterium exchange mass spectrometry (H/D exchange MS), which measures the solution-phase incorporation of deuterium into proteins: The H/D exchange rate for 90 peptides of peptic digest (89% sequence coverage) was compared between Z-ICR alone and Z-ICR plus SCB4380 (Supplementary Fig. S3). Among these peptide fragments, two peptides containing the catalytic Cys residue of D1 showed significant decreases in the H/D exchange rate by incubation with SCB4380 (Fig. 5C), suggesting its binding site. Taken together with the inhibition mode and the results of the native MS under non-denaturing conditions, we concluded that SCB4380 specifically bound to the catalytic pocket of PTPRZ-D1.

**Figure 7:**
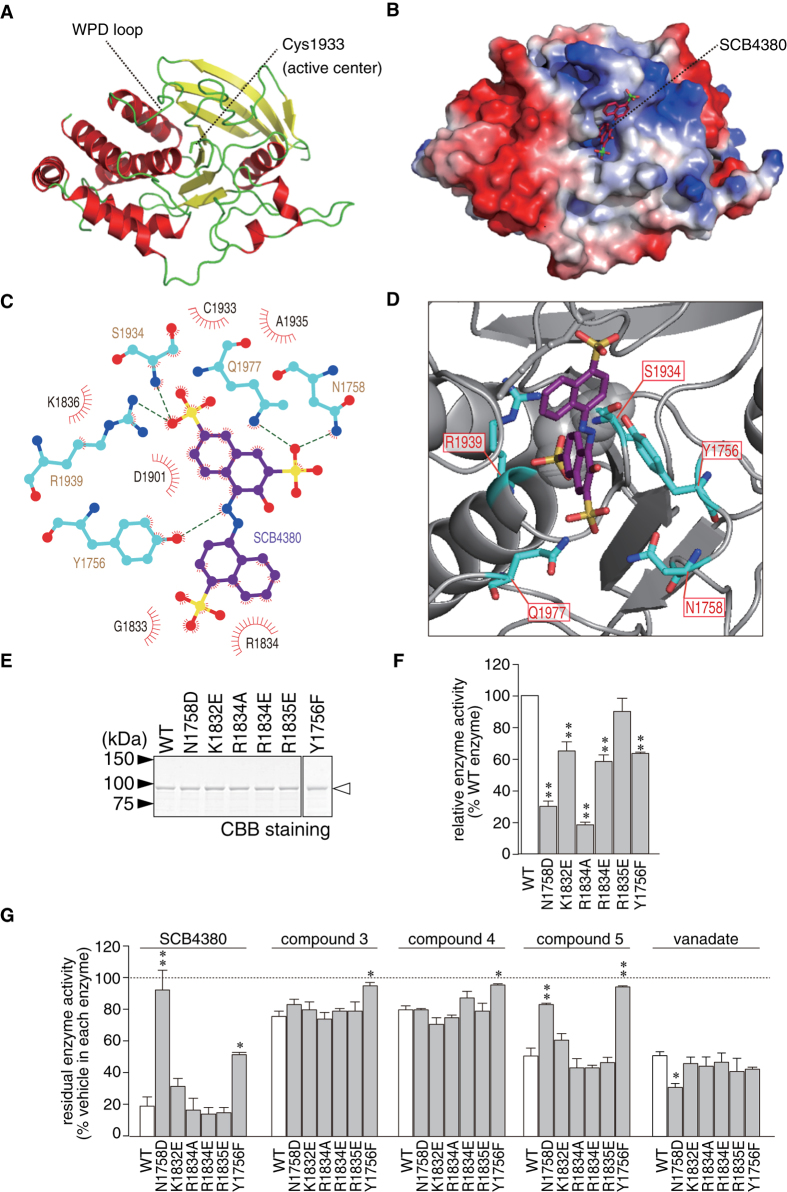
Predicted binding mode of SCB4380 to the PTPRZ catalytic pocket. (**A**) X-ray structure of human PTPRZ-D1 (PDB ID: 5AWX). The representation is colored red for the α-helix, yellow for the β-sheet, and green for the loop. (**B**–**D**) *In silico* docking of SCB4380 into the active site using a closed structure model of human PTPRZ-D1. Whole view of the best docking pose of SCB4380 shown by stick representation (**B**). The protein surface is colored in blue (positive charge) to red (negative charge) according to the surface potential calculated using vacuum electrostatics by PyMOL. The docking search was restricted to the catalytic pocket based on H/D exchange MS data. LigPlot representation of SCB4380 bound to the D1 active-site (**C**). The predicted hydrogen bonds (broken green lines), and residues (red spoked arcs) involved in the hydrophobic contact are shown in the LigPlot. The enlarged 3D image (**D**). Amino acid residues predicted to form hydrogen bonds with SCB4380 are shown by thick representation. (**E**,**F**) Effects of site-directed mutagenesis on PTPRZ catalytic activity. Purified GST-fused proteins were analyzed by SDS-PAGE and CBB staining (**E**), and their activities were determined using an aliquot of 5 nM enzymes with 20 μM pCAP-GIT1_549–556_ (**F**). Data are presented as a relative value to the wild-type enzyme (mean ± S.E., *n* = 3 to 5). **P < 0.01, significantly different from the wild-type by ANOVA and Dunnett's test. (**G**) Inhibitor sensitivity. The inhibition assay was performed by adjusting relative enzyme activity with 20 μM pCAP-GIT1_549–556_ by adding compounds (10 μM each) or vanadate (200 μM). Residual enzymatic activity is presented as a relative value to the DMSO control in each enzyme (mean ± S.E., *n* = 3). *P < 0.05 or **P < 0.01, significantly different from the wild-type enzyme in the same group by ANOVA and Dunnett's test.

We achieved the crystallization of the intracellular D1 of human PTPRZ using the sitting-drop vapor-diffusion method, and succeeded in solving the structure at 1.86 Å resolution (Fig. 7A, see also Supplementary Fig. S4) by applying the molecular replacement method using the apo structure of the D1 of PTPRG (3QCB) as a search model. In order to understand the structural basis of the inhibitory mechanism of SCB4380 in details, we challenged to determine the PTPRZ-SCB4380 complex structure. However, our attempts to soak SCB4380 into the PTPRZ-D1 crystals, or to cocrystallize SCB4380 with PTPRZ-D1 domain were not successful. We therefore performed docking studies for two tautomers of SCB4380 to the two D1 structures of PTPRZ (the catalytic WPD-loop was modeled in two states, open- or closed-loop structure). Among the four combinations, the best docking pose was predicted between the azo tautomer and the closed D1 structure (Fig. 7B–D). The sulfonate group at the 2-position of the naphthalenedisulfonic acid moiety was predicted to form hydrogen bonds with the side chains of Asn1758 and Gln1977, respectively. Asn1758 residue locates near the C-terminal side of the pTyr-recognition loop (Asn1750 to Tyr1756) that reportedly affects substrate specificity^27^. Notably, Asn1758 is one of the non-conserved residues that are reportedly involved in the recognition of PTP substrates and non-peptide inhibitors^27^, suggesting that this interaction may be responsible for the inhibitory effects of SCB4380 (This Asn residue is also present in PTPRG). Gln1977 is a conserved residue in the Q loop (Gln1977 to Phe1984, the motif sequence is QTXXQYXF), and its side chain has been reported to mediate the hydrolysis of the cysteinyl phosphate, a catalytic intermediate^27^. The other sulfonate group at the 7-position of the naphthalenedisulfonic acid moiety and the azo group of SCB4380 potentially form hydrogen bonds with conserved Ser1934 and Arg1939 in the PTP loop (Pro1928 to Gly1941, the motif is (PXXVHCSAGXGRTG) and Tyr1756, respectively. Thus, the binding of SCB4380 was expected to prevent substrate access and hydrolysis.

We performed site-directed mutagenesis to test the structural prediction of binding. Six mutants of Z-ICR at five residues exhibited variable decreases in enzymatic activity (Fig. 7E,F), and their impact on inhibitor sensitivity was estimated with the same fixed enzyme activity (Fig. 7G). Since the Asn at the position 1758 is most frequently substituted with Asp in the PTP family members including CD45 and PTP1B (ref. 27), Asn1758 was substituted with Asp (N1758D). The N1758D mutant was resistant to inhibition by SCB4380 and compound 5, with no changes in sensitivity to others (compound 3 and compound 4). This result supported the presence of the predicted interaction between the sulfonate at the 2-position and the amino group in the side chain of Asn1758. Tyr1756 is well conserved among PTP family members, though substituted with Phe in PTPN21. The Y1756F mutant impaired the inhibition by SCB4380 markedly and by its analogues weakly, but not by vanadate, supporting the prediction that the hydroxyl group of Tyr1756 interacts with the azo group. Mutations of the other predicted residues such as Gln1977, Ser1934, and Arg1939 were omitted because these residues are conserved in PTPs and play essential roles in catalysis^27^. Compound 5 and compound 6 lacking the sulfonate at the 4-postion of another naphthalene weakly inhibited PTPRZ (see Fig. 4B), and this was probably attributed to the loss of the electrostatic interaction with the positively-charged side chain of Arg1834 or its neighbors (K1832 or Arg1835); while, these interactions were not predicted by our docking studies. We attempted to test this notion, but observed no significant changes in the inhibitor sensitivity for R1834A, R1834E, K1832E, or R1835E. The results of site-directed mutagenesis generally supported the structural model for SCB4380 binding to the catalytic pocket of PTPRZ.

### Blockade of cell proliferation and migration by liposome-mediated delivery of SCB4380

**Figure 8:**
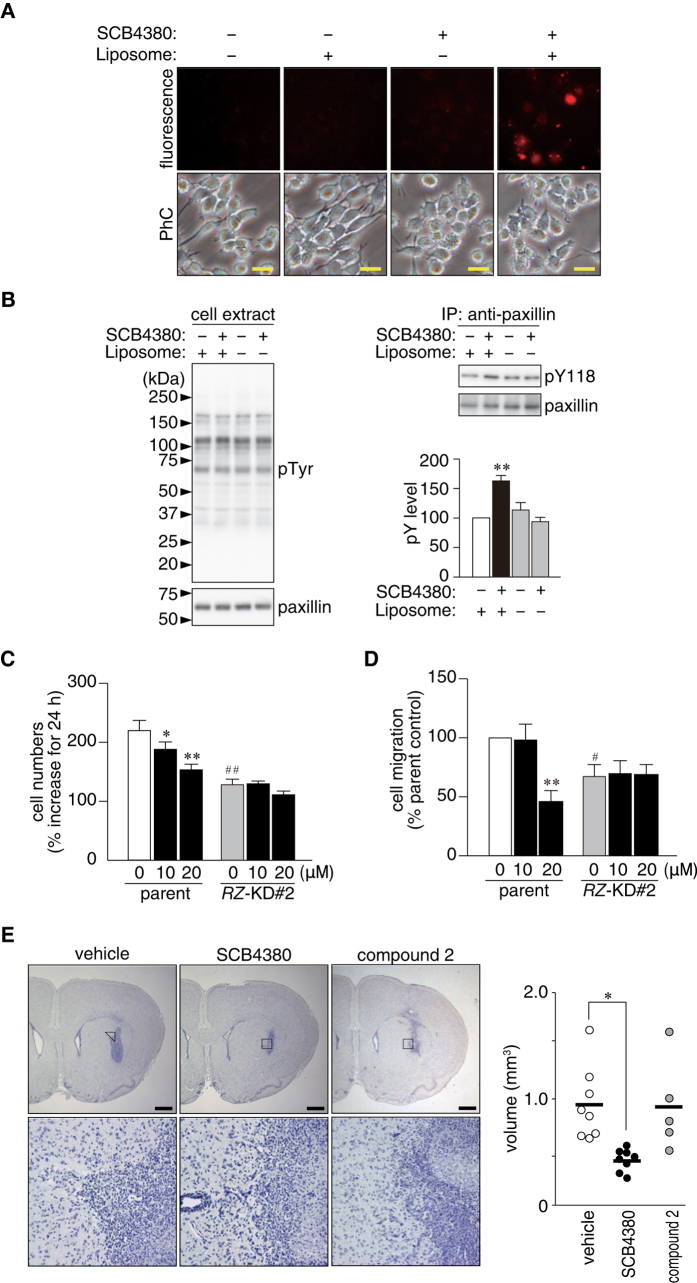
Suppressive activity of the SCB4380/liposome complex on malignant phenotypes of C6 glioblastoma cells. (**A**) Liposome-mediated SCB4380 delivery. Cells were incubated with the indicated combinations of SCB4380 and liposome for 3 h. Scale bars, 20 μm. (**B**) Effects of the SCB4380/liposome treatment. After the treatment for 30 min, paxillin phosphorylation at Tyr118 was analyzed by Western blotting. Data are the mean ± S.E. (*n* = 3). *P < 0.05, significantly different from liposome-treated control cells by ANOVA and Scheffé’s *post hoc* tests. (**C**) Cell proliferation assay. Cells were counted 1 and 25 h after the addition of the liposome complex, and increases in 24 h were presented as relative values. No differences were observed in cell numbers at 1 h, indicating no effects on cellular adhesion (Supplementary Fig. S5D). Data are the mean ± S.E. (*n* = 4). *P < 0.05 or **P < 0.01, significantly different from vehicle-treated cells in the same group, and ^##^*P* < 0.01, significantly different from parent cells treated with vehicle by ANOVA and Scheffé’s *post hoc* tests. (**D**) Boyden chamber assay. C6 cells treated were allowed to migrate across transwell inserts for 2 h. Data are the mean ± S.E. (*n* = 5). **P < 0.01, significantly different from vehicle-treated cells in the same group, and ^#^*P* < 0.05, significantly different from vehicle-treated parent cells by ANOVA and Scheffé’s *post hoc* tests. Representative images are shown in Supplementary Fig. 5E. (**E**) Effects of the SCB4380/liposome on tumor formation of C6 cells. C6 cells transplanted into rat brains were allowed to grow for 7 days. One day after surgery, 5 μl of the liposome complexes (1 mM), or vehicle (0.9% NaCl) was icv injected daily for five days. Representative sections are shown together with an enlargement of the boxed area. Scale bars, 1 mm. The graph shows tumor volume (vehicle, *n* = 8; SCB4380/liposome, *n* = 8; compound 2/liposome, *n* = 5). The horizontal bars indicate the average of each group. *P < 0.05, significantly different from the vehicle-treated cells by the Student’s *t*-test.

Negatively charged arylsulfonic acids of SCB4380 were unfavorable for cellular uptake, as was commonly reported for many competitive inhibitors of PTP family members^4^. Indeed, membrane permeability of SCB4380 was deficient in conventional parallel artificial membrane permeability assays (PAMPA). However, we found that SCB4380 was efficiently delivered into living C6 glioblastoma cells using a cationic liposome reagent (Fig. 8A, also see Supplementary Fig. S5A). As observed in stable *Ptprz*-knockdown cells, the Tyr118 phosphorylation of paxillin was significantly increased in C6 cells treated with the SCB4380/liposome complex (right panels in Fig. 8B), notwithstanding no significant changes in the overall tyrosine phosphorylation pattern or paxillin expression (left panels in Fig. 8B). These results indicated that SCB4380 was able to inhibit phosphatase activity of PTPRZ, once it was delivered into cells.

And so, we next investigated whether the SCB4380/liposome complex can suppress the malignant phenotypes of glioblastoma. The SCB4380/liposome treatment dose-dependently reduced the proliferation and migration of C6 glioblastoma cells *in vitro*, but not those of *Ptprz*-knockdown C6 cells as control cells (Fig. 8C,D; see also Supplementary Fig. S5D,E). On the other hand, compound 2, the most structurally related compound to SCB4380, which showed almost no inhibition for PTPRZ (see Fig. 4), did not show suppressive effects on the growth of C6 cells (Supplementary Fig. S6), excluding the possibility of off-target effects of SCB4380. Noteworthily, we found that PTPRG is also expressed in C6 cells, however, the siRNA knockdown of *Ptprg* did not affect their migration (Supplementary Fig. S2). These results indicate that SCB4380 exerted these effects through inhibition of phosphatase activity of PTPRZ. The larger effect of SCB4380 on migration than proliferation might be due to a chemical instability of this compound in living cells (Supplementary Fig. S5B,C), as it takes 2 h for migration assay but 24 h for proliferation assay.

We then examined *in vivo* effects by treating the orthotopic C6 glioblastoma tumor model with repeated icv administrations of the SCB4380/liposome complex. Tumor sizes were significantly smaller in SCB4380/liposome-treated animals than in vehicle-treated and compound 2/liposome-treated controls (Fig. 8E). Thus, SCB4380 inhibited malignant growth and migration of glioblastoma cells both *in vitro* and *in vivo*, when it was delivered into cells using a cationic liposome reagent. Taken altogether, these results indicate that phosphatase activity of PTPRZ is responsible for the malignant phenotypes of C6 glioblastoma.

## Discussion

In the present study, we identified SCB4380 as a potent competitive inhibitor of R5 RPTP subfamily members (PTPRZ and PTPRG) through high-throughput screening. Molecular docking to the 3D structure of the D1 domain of PTPRZ and mutational experiments predicted the structural basis of this selective inhibition. The intracellular delivery of SCB4380 by liposome carriers inhibited cellular PTPRZ activity, and thereby suppressed cell migration and proliferation *in vitro* as well as tumor growth *in vivo* in rat C6 glioblastoma cells. These results demonstrated that the catalytic activity of PTPRZ was responsible for the malignancy of glioblastomas. Therefore, the selective inhibition of PTPRZ represents a promising approach for glioma therapy.

The contribution of the intrinsic phosphatase activity of PTPRZ to the malignancy of glioblastomas has remained unclear. We herein showed that the phosphatase activity of PTPRZ was associated with the malignancy of glioblastomas. We, for the first time, discovered SCB4380 (known as Acid Red 27 or amaranth) as the most potent PTPRZ inhibitor. Synthetic red tar dyes including amaranth and its structural analogues are widely used in food and cosmetic industries. We found that SCB4380, but not its chemical analogues, exhibited strong inhibitory effects on PTPRZ. The results obtained by recent H/D exchange and native MS techniques provided clear evidence for the stoichiometric and specific binding of SCB4380 to the catalytic site of PTPRZ. Furthermore, native MS results excluded the binding of SCB4380 inducing the protein aggregation or oxidization of PTPRZ-ICR, which have been often identified as the mechanisms underlying the false-positive inhibition of PTPs (ref. 4). The molecular docking experiment successfully predicted the binding mode of SCB4380 to the catalytic pocket, which was then verified experimentally.

The extracellular domain of PTPRZ is known to bind with various cell adhesion molecules, extracellular matrix molecules, and growth factors (refs 18, 19, 20, 21, 22, 23, 24, 25, 26, 27, 28 and cited therein). However, the functional significance of these interactions has not yet been elucidated in detail, except for the binding of pleiotrophin^18^,^19^,^20^,^21^,^22^,^23^,^24^,^25^,^26^,^27^,^28^. On the other hand, PTPRZ receptor proteins undergo metalloproteinase- and γ-secretase-mediated proteolytic processing on the cell surface, and are ultimately converted to the cytoplasmic PTPRZ fragment (Z-ICF) (ref. 21). Also, metalloproteinases are known to play important roles in tumor growth and the invasion of cancer cells^29^. A very recent study reported that the extracellular segment of PTPRZ had positive effects on the migration of glioblastoma by using a different cell line^19^. Thus, the interaction between the extracellular region of PTPRZ-B and these molecules may also contribute to the invasion of glioblastomas, while our results showed that overexpression of Z-ICF with normal phosphatase activity promotes the migration of C6 cells. In the present study, the marked accumulation of Z-ICF was detected in C6 glioblastoma cells. Pleiotrophin and its family member, midkine are also overexpressed together with PTPRZ in a number of tumors^30^, and promote the migration and invasiveness of glioblastoma cells^30^,^31^. Z-ICF cannot interact anymore with the extracellular inhibitory ligands. Therefore, the tumor growth-promoting effects induced by the pleiotrophin family mediated through receptors other than PTPRZ, such as anaplastic lymphoma kinase^30^,^31^.

Regarding the signaling pathways downstream of PTPRZ, we recently identified a conserved motif of Glu/Asp-Glu/Asp-Glu/Asp-Xaa-Ile/Val-Tyr(P)-Xaa (Xaa is not an acidic residue) in physiologically relevant substrates of PTPRZ, including paxillin at the tyrosine residue 118 site (Tyr118) (refs 17, 18, 19, 20, 21, 22, 23), G protein-coupled receptor kinase-interactor 1 (GIT1) at Tyr554, GTPase-activating protein for Rho GTPase (p190RhoGAP) at Tyr1105, and membrane-associated guanylate kinase, WW and PDZ domain-containing 1 (MAGI1) at Tyr373. Paxillin, GIT1, and p190RhoGAP are key regulators of focal adhesion turnover and invadosome formation, all of which are crucial for the motility and invasiveness of cancer cells^32^. The specific dephosphorylation of GIT1 at Tyr554 by PTPRZ appears to fine-tune the targeting of GIT1 complexes to appropriate subcellular locations in order to ensure coordinated cell motility^17^. We considered the accelerated phosphorylation-dephosphorylation cycles of these substrate molecules to be involved in migration and invasiveness.

MAGI1 is a binding partner of the tumor suppressor PTEN (phosphatase and tensin homolog)^33^. The loss of *PTEN* is a frequent event in high-grade gliomas, and is associated with a poorer prognosis^34^. PTEN counteracts the most critical cancer-promoting PI3 kinase-AKT pathway by dephosphorylating the PI3 kinase product, PI 3,4,5-tris-phosphate (PIP_3_) to PIP_2_. Although the functional role of MAGI1 phosphorylation at Tyr373 currently remains unclear, PTEN is known to cooperate with MAGI1 proteins in order to block PI3 kinase-AKT, which is important for stabilizing of cell-cell contacts and suppressing of tumor invasiveness^34^. PTEN (ref. 33) and PTPRZ (ref. 23) both bind to the same second PDZ domain of MAGI1 with their carboxy terminal PDZ-binding sequences, and, thus, the two may be mutually exclusive. The signaling pathway responsible for malignant proliferation needs to be investigated in more detail in future studies.

In summary, we herein demonstrated that the enzymatic activity of PTPRZ was responsible for the malignancy of glioblastomas. The aberrant expression of PTPRZ has also been observed in other tumors, such as neuroblastoma^35^, cutaneous melanomas^35^, gastric cancers^36^, and small-cell lung carcinoma^37^, suggesting the involvement of PTPRZ signaling in general tumor malignancy. SCB4380 (Acid Red 27, amaranth) shows high solubility in water, low membrane permeability, and substantial instability in living cells. Considering the possibility of use as a therapeutic drag, the latter two prosperities are not desirable. Combination with drug delivery systems (DDS) including liposome may be effective to overcome the problem of membrane permeability. However, it would be better to continue our efforts to develop derivatives of SCB4380 or to search for completely different chemical agents for the treatment of gliomas. Our structural model of the SCB4380-PTPRZ complex is informative for this purpose.

## Methods

### Chemicals

The high purity chemical of SCB4380; trisodium 3-hydroxy-4-[(4-sulfonato-1-naphthyl) diazenyl]-2,7-naphthalenedisulfonate (compound 1) was purchased from Sigma-Aldrich (amaranth dye, ≥98% purity, cat no. 87612). SCB4380 analogue, new coccine; trisodium 7-hydroxy-8-[(4-sulfonato-1-naphthyl)diazenyl]-1,3-naphthalenedisulfonate (compound 2, cat no. 147-06542) were purchased from Wako Pure Chemical Industries, while Acid Red 13; disodium 6-hydroxy-5-[(4-sulfonato-1-naphthyl)diazenyl]-2-naphthalene sulfonate (compound 3, A1243), Acid Red 88; sodium 4-(2-hydroxy-1-naphthalenylazo)-1-naphthalenesulfonate (compound 4, F0087), bordeaux red; disodium 3-hydroxy-4-(1-naphthyldiazenyl)-2,7-naphthalenedisulfonate (compound 5, B0779), β-naphthol violet; sodium 3-hydroxy-4-[(4-nitrophenyl)diazenyl]-2,7-naphthalenedisulfonate (compound 6, N0037), disodium 1-nitroso-2-naphthol-3,6-disulfonate monohydrate (compound 7, N0268), disodium 3-hydroxy-2,7-naphthalenedisulfonate (compound 8, N0029), 4-amino-1-naphthalenesulfonic acid (compound 9, A0344), and sodium 1-naphthalenesulfonate (compound 10, N0015) were from Tokyo Chemical Industry Co., Ltd.

### In *vitro* dephosphorylation assays

Two hundred microliters of pCAP-peptide substrates (20 μM) in a three-component buffer of pH 7.0 (100 mM acetate, 50 mM Tris, and 50 mM Bis-Tris) containing 5 mM DTT and 0.01% (*v/v*) Brij-35 were preincubated in a cuvette at room temperature. The reaction was initiated by adding 2 μl of an enzyme solution, and incubated for ~100 s; the amount of the enzyme was adjusted to give a convenient rate. Compounds were then added to the same cuvette at a 1/100 volume. In each experiment, the hydrolysis of the pCAP residue was continuously monitored as an increase in fluorescence at 460 nm (excitation at 334 nm) using a spectrofluorometer (FI-4500, Hitachi). We measured the slope of the linear increase of the fluorescence signal as an indicator of catalytic activity, which was obtained during the 40 s before and after the addition of a compound. The inhibitory effects of each compound were determined by the ratio of the activities, and indicated as the relative value to that of DMSO control.

### Mass spectrometry under non-denaturing conditions

PTPRZ-ICR solution (23.1 mg/ml in the stock solution) was buffer exchanged to 100 mM ammonium acetate, pH 7.0 by passing through a Bio-Spin 6 column (Bio-Rad), diluted 100-fold with the same buffer, and then kept on ice. Ten microliter aliquots were mixed with equal volumes of each compound solution, and the samples were immediately analyzed by nanoflow electrospray using in-house made gold-coated glass capillaries (~2 μl sample was loaded for each analysis). Spectra were recorded on a SYNAPT G2-S*i* HDMS mass spectrometer (Waters) in the positive ionization mode at 1.36 kV with 150 V of a sampling cone voltage and source offset voltage, 0 V of trap and transfer collision energy, and 2 ml/min trap gas flow. Spectra were calibrated using 1 mg/ml cesium iodide, and analyzed by Mass Lynx software (Waters).

### Isothermal titration calorimetry (ITC)

ITC experiments were performed at 298.15 K in a buffer (20 mM Tris-HCl, pH 7.5 containing 5 mM DTT and 150 mM NaCl) using a MicroCal iTC_200_ (Malvern). AR27 at a concentration of 250 μM was titrated into 20.9 μM of PTPRZ-ICR. The titration sequence included a single 0.4 μl injection followed by 19 injections of 2 μl each at an interval of 120 s between the injections. NITPIC (ref. 38) was used to analyze the raw data obtained from the experiments.

### Hydrogen/Deuterium exchange mass spectrometry (H/D exchange MS)

Analyses were performed using the H/D exchange system with DynamX 2.0 software (Waters). In order to initiate H/D exchange, PTPRZ-ICR (80 μM), which was incubated with or without SCB4380 (200 μM), was diluted 19-fold with 20 mM Tris-DCl, pD 7.5, containing 5 mM DTT and 150 mM NaCl. The exchange reaction was carried out at 20 °C for durations of 0.5, 1, 10, 60, and 240 min, and stopped by adding a double volume of cold quench and denature buffer (200 mM Tris-HCl, pH 2.1 containing 150 mM NaCl, 4 M guanidine HCl, and 250 mM TCEP). The quenched samples were injected into the H/D exchange system, digested by flowing through an online immobilized pepsin column (Applied Biosystems), and the resulting peptide mixture was desalted on the VanGuard precolumn (Waters) for 6 min, followed by separation on a reverse phase UPLC column at a flow rate of 40 μl per min. An acetonitrile gradient from 8 to 40% (*v/v*), with 0.1% (*v/v*) formic acid, over 9 min was used for peptide separation. The eluent was directly injected into a SYNAPT G1 mass spectrometer (Waters) running in the ESI positive mode.

Data were acquired in the full MS scan over an *m/z* range of 100–2000 with lock mass spray correction using the Glu-fibrinogen B peptide. The peptides obtained by online pepsin digestion were identified by running a separate experiment without deuterium labeling. MS/MS data were analyzed using the ProteinLynx Global Server (PLGS, Waters) and DynamX software to identify peptides with sufficient signals and confidence to be reliably used for deuterium labeling analyses.

The number of deuterium labels in each peptide at different time points was calculated using DynamX software, and the H/D exchange differential was plotted to compare deuterium labeling of respective peptides in PTPRZ alone and the PTPRZ/SCB4380 complex.

### Crystallization and structure determination

The initial screening of crystallization conditions was performed using Morpheus (Molecular Dimensions). Crystals of human PTPRZ-D1 (amino acid residues, 1698–2000) were obtained by the sitting-drop vapor-diffusion method using a crystallization solution containing 10% (*w/v*) PEG8000, 20% (*v/v*) ethylene glycol, 30 mM sodium fluoride, 30 mM sodium bromide, 30 mM sodium iodide, and 0.1 M Tris-Bicine, pH 8.5 at 277 K. Crystals were flash-frozen in a cold nitrogen stream at about 100 K, and X-ray diffraction data were collected using an RAXIS IV^++^ imaging-plate area detector mounted on a Rigaku MicroMax-007 rotating-anode source with CuKα radiation (λ = 1.5418 Å, 40 kV, 20 mA). All data were processed and scaled with HKL2000 (ref. 39). The crystal structure was solved by the molecular replacement method using the apo structure of PTPRG (PDB ID, 3QCB). A molecular-replacement calculation was performed using AMoRe (ref. 40). The structure model was fit using Coot (ref. 41) and refined with Refmac-5 (ref. 42). Figures were created by Pymol (DeLano Scientific).

### Molecular docking simulation

After the removal of water and ion molecules, Lys1878-Gly1888 residues that were disordered in the crystals of apo PTPRZ-D1 were modeled using the apo structure of PTPRG (2NLK). Moreover, the WPD loop (Tyr1896 to Ser1908) positioned to facilitate the cleavage of the phospho-Tyr substrate^27^ was modeled using the crystal structure of the PTPRG-vanadate complex (3QCC). Prior to docking studies, energy minimizations of the D1 structure were performed using the OPLS2005 force field^43^ on the MacroModel program (Schrödinger, LLC, version 9.9). All three sulfonate groups of SCB4380 were regarded as being singly ionized, and both tautomers, the azo and hydrazone forms, were docked into the modeled PTPRZ structure using the GOLD program^44^, respectively: We here defined the docking area within a radius of 20 Å from the sulfur atom of Cys1933 (based on the results of the inhibition mechanism, and the MS experiments), and calculated 30 solutions for each tautomer. The best fitness score was found for azo SCB4380. Interactions between PTPRZ and SCB4380 were shown using LigPlot^+^ (ref. 45). Hydrogen bonds and non-bonded contacts were assigned by HBPLUS (ref. 46).

### SCB4380/liposome and compound 2/liposome complexes

Lipofectamine 2000 reagent (1 mg/ml, cat no. 11668-027, Invitrogen) was 25-fold diluted with the Opti-MEM (x1) + GlutaMAX medium (cat no. 51985-034, Invitrogen), and incubated for 5 min at room temperature. In order to prepare the SCB4380 (or compound 2)/liposome complex, an equal volume of 2 mM SCB4380 (or compound 2) in the same solvent was added, gently mixed, and incubated at least for 30 min. The resulting mixture was immediately used as a 1 mM SCB4380 (or compound 2)/liposome solution. Regarding the intracerebroventricular injection, 0.9% NaCl was used as the solvent.

### Intracerebral allografts and Histological analyses

All animal experiments in this study were approved by the Institutional Animal Care and Use Committee of the National Institutes of Natural Sciences, Japan, and were performed in accordance with the institutional guidelines of the care and use of laboratory animals. All surgeries were performed under isoflurane anesthesia, and all efforts were made to minimize suffering and number of animals used in this study.

Wistar rats (males, 3 weeks old) were anesthetized with isoflurane during surgical procedures. Parental or *Ptprz-*knockdown C6 cells (5 × 10^5^ cells) were suspended in 5 μl of PBS, and the cell suspension was stereotactically transplanted into the left striatum with a Hamilton syringe at a depth of 5 mm (coordinates with respect to the bregma: 0.7 mm anterior and 3 mm lateral). Regarding the icv injection of compounds, a stainless guide cannula (22 gauge, 7.4 mm long, prepared in house) was implanted into the left lateral brain ventricle (coordinates with respect to bregma: 0.9 mm posterior, 1.4 mm lateral, and 2.7 mm ventral) during surgery. One day after surgery, 1 mM SCB4380/liposome complex solution was icv injected in a volume of 5 μl over 5 min, and repeated daily for a total of five days. All rats were monitored daily for spontaneous pain and other abnormal behaviors. Seven days after surgery, animals were intracardially perfused with 0.9% NaCl, followed by 10% neutral formalin (Wako), and the brains were post-fixed overnight in the same fixative. Paraffin-embedded brains were serially sectioned at 7-μm intervals in the coronal plane, and stained with hematoxylin to estimate tumor volumes. Digital photomicrographs of each specimen were taken with an Eclipse microscope Ci-L with a DS-Fi2 CCD (Nikon), and analyzed using Adobe Photoshop CS6 (Adobe Systems).

## Additional Information

**How to cite this article**: Fujikawa, A. *et al.* Small-molecule inhibition of PTPRZ reduces tumor growth in a rat model of glioblastoma. *Sci. Rep.* **6**, 20473; doi: 10.1038/srep20473 (2016).

## Supplementary Material

Supplementary Information

## References

[b1] Louis D. N. *et al.* The 2007 WHO classification of tumours of the central nervous system. Acta Neuropathol. 114, 97–109 (2007). 17618441 10.1007/s00401-007-0243-4 PMC1929165

[b2] Stupp R. *et al.* Effects of radiotherapy with concomitant and adjuvant temozolomide versus radiotherapy alone on survival in glioblastoma in a randomised phase III study: 5-year analysis of the EORTC-NCIC trial. Lancet Oncol. 10, 459–466 (2009). 19269895 10.1016/S1470-2045(09)70025-7

[b3] Navis A. C. *et al.* Protein tyrosine phosphatases in glioma biology. Acta Neuropathol. 119, 157–175 (2010). 19936768 10.1007/s00401-009-0614-0 PMC2808538

[b4] Barr A. J. Protein tyrosine phosphatases as drug targets: strategies and challenges of inhibitor development. Future Med. Chem. 2, 1563–1576 (2010). 21426149 10.4155/fmc.10.241

[b5] Zhai Y. F. *et al.* Increased expression of specific protein tyrosine phosphatases in human breast epithelial cells neoplastically transformed by the *neu* oncogene. Cancer Res. 53, 2272–2278 (1993). 8097963

[b6] Julien S. G. *et al.* Protein tyrosine phosphatase 1B deficiency or inhibition delays ErbB2-induced mammary tumorigenesis and protects from lung metastasis. Nat. Genet. 39, 338–346 (2007). 17259984 10.1038/ng1963

[b7] Bentires-Alj M. & Neel B. G. Protein-tyrosine phosphatase 1B is required for HER2/*Neu*-induced breast cancer. Cancer Res. 67, 2420–2424 (2007). 17347513 10.1158/0008-5472.CAN-06-4610

[b8] Krishnan N. *et al.* Targeting the disordered C terminus of PTP1B with an allosteric inhibitor. Nat. Chem. Biol. 10, 558–566 (2014). 24845231 10.1038/nchembio.1528 PMC4062594

[b9] Sugawa N., Ekstrand A. J., James C. D. & Collins V. P. Identical splicing of aberrant epidermal growth factor receptor transcripts from amplified rearranged genes in human glioblastomas. Proc. Natl. Acad. Sci. USA 87, 8602–8606 (1990). 2236070 10.1073/pnas.87.21.8602 PMC55005

[b10] Paez J. G. *et al.* EGFR mutations in lung cancer: correlation with clinical response to gefitinib therapy. Science 304, 1497–1500 (2004). 15118125 10.1126/science.1099314

[b11] De Witt Hamer P. C. Small molecule kinase inhibitors in glioblastoma: a systematic review of clinical studies. Neuro Oncol. 12, 304–316 (2010). 20167819 10.1093/neuonc/nop068 PMC2940593

[b12] Müller S. *et al.* A role for receptor tyrosine phosphatase ζ in glioma cell migration. Oncogene 22, 6661–6668 (2003). 14555979 10.1038/sj.onc.1206763

[b13] Ulbricht U. *et al.* Expression and function of the receptor protein tyrosine phosphatase ζ and its ligand pleiotrophin in human astrocytomas. J. Neuropathol. Exp. Neurol. 62, 1265–1275 (2003). 14692702 10.1093/jnen/62.12.1265

[b14] Patel A. P. *et al.* Single-cell RNA-seq highlights intratumoral heterogeneity in primary glioblastoma. Science 344, 1396–1401 (2014). 24925914 10.1126/science.1254257 PMC4123637

[b15] Foehr E. D. *et al.* Targeting of the receptor protein tyrosine phosphatase β with a monoclonal antibody delays tumor growth in a glioblastoma model. Cancer Res. 66, 2271–2278 (2006). 16489031 10.1158/0008-5472.CAN-05-1221

[b16] Fujikawa A. *et al.* Mice deficient in protein tyrosine phosphatase receptor type Z are resistant to gastric ulcer induction by VacA of *Helicobacter pylori*. Nat. Genet. 33, 375–381 (2003). 12598897 10.1038/ng1112

[b17] Fujikawa A., Matsumoto M., Kuboyama K., Suzuki R. & Noda M. Specific dephosphorylation at tyr-554 of git1 by ptprz promotes its association with paxillin and hic-5. PLoS ONE 10, e0119361 (2015). 25742295 10.1371/journal.pone.0119361 PMC4351203

[b18] Maeda N. & Noda M. Involvement of receptor-like protein tyrosine phosphatase ζ/RPTPβ and its ligand pleiotrophin/heparin-binding growth-associated molecule (HB-GAM) in neuronal migration. J. Cell Biol. 142, 203–216 (1998). 9660874 10.1083/jcb.142.1.203 PMC2133018

[b19] Bourgonje A. M. *et al.* Intracellular and extracellular domains of protein tyrosine phosphatase PTPRZ-B differentially regulate glioma cell growth and motility. Oncotarget. 5, 8690–8702 (2014). 25238264 10.18632/oncotarget.2366 PMC4226714

[b20] Ulbricht U., Eckerich C., Fillbrandt R., Westphal M. & Lamszus K. RNA interference targeting protein tyrosine phosphatase ζ/receptor-type protein tyrosine phosphatase β suppresses glioblastoma growth *in vitro* and *in vivo*. J. Neurochem. 98, 1497–1506 (2006). 16923162 10.1111/j.1471-4159.2006.04022.x

[b21] Chow J. P., Fujikawa A., Shimizu H., Suzuki R. & Noda M. Metalloproteinase- and γ-secretase-mediated cleavage of protein-tyrosine phosphatase receptor type Z. J. Biol. Chem. 283, 30879–30889 (2008). 18713734 10.1074/jbc.M802976200 PMC2662165

[b22] Grobben B., De Deyn P. P. & Slegers H. Rat C6 glioma as experimental model system for the study of glioblastoma growth and invasion. Cell Tissue Res. 310, 257–270 (2002). 12457224 10.1007/s00441-002-0651-7

[b23] Fujikawa A. *et al.* Consensus substrate sequence for protein-tyrosine phosphatase receptor type Z. J. Biol. Chem. 286, 37137–37146 (2011). 21890632 10.1074/jbc.M111.270140 PMC3199461

[b24] Iwasaki T. *et al.* Involvement of phosphorylation of Tyr-31 and Tyr-118 of paxillin in MM1 cancer cell migration. Int. J. Cancer 97, 330–335 (2002). 11774284 10.1002/ijc.1609

[b25] Hernández H. & Robinson C. V. Determining the stoichiometry and interactions of macromolecular assemblies from mass spectrometry. Nat. Protoc. 2, 715–726 (2007). 17406634 10.1038/nprot.2007.73

[b26] Salmeen A. *et al.* Redox regulation of protein tyrosine phosphatase 1B involves a sulphenyl-amide intermediate. Nature 423, 769–773 (2003). 12802338 10.1038/nature01680

[b27] Andersen J. N. *et al.* Structural and evolutionary relationships among protein tyrosine phosphatase domains. Mol. Cell Biol. 21, 7117–7136 (2001). 11585896 10.1128/MCB.21.21.7117-7136.2001 PMC99888

[b28] Fukada M. *et al.* Protein tyrosine phosphatase receptor type Z is inactivated by ligand-induced oligomerization. FEBS lett. 580, 4051–4056 (2006). 16814777 10.1016/j.febslet.2006.06.041

[b29] Gialeli C., Theocharis A. D. & Karamanos N. K. Roles of matrix metalloproteinases in cancer progression and their pharmacological targeting. FEBS J. 278, 16–27 (2011). 21087457 10.1111/j.1742-4658.2010.07919.x

[b30] Wellstein A. ALK receptor activation, ligands and therapeutic targeting in glioblastoma and in other cancers. Front Oncol. 10.3389/fonc.2012.00192 eCollection (2012). PMC3525999 23267434

[b31] Lu K. V. *et al.* Differential induction of glioblastoma migration and growth by two forms of pleiotrophin. J. Biol. Chem. 280, 26953–2664 (2005). 15908427 10.1074/jbc.M502614200

[b32] Parsons J. T., Slack-Davis J. K., Tilghman R. W., Iwanicki M. & Martin K. H. Integrin signaling: cell migration, proliferation, and survival. Intercellular Signaling in Development and Disease: Cell Signaling Collection (Elsevier, California, 2011).

[b33] Kotelevets L. *et al.* Implication of the MAGI-1b/PTEN signalosome in stabilization of adherens junctions and suppression of invasiveness. FASEB J. 19, 115–117 (2005). 15629897 10.1096/fj.04-1942fje

[b34] Kwon C. H. *et al.* *Pten* haploinsufficiency accelerates formation of high-grade astrocytomas. Cancer Res. 68, 3286–3294 (2008). 18451155 10.1158/0008-5472.CAN-07-6867 PMC2760841

[b35] Goldmann T., Otto F. & Vollmer E. A receptor-type protein tyrosine phosphatase PTP zeta is expressed in human cutaneous melanomas. Folia Histochem. Cytobiol. 38, 19–20 (2000). 10763119

[b36] Wu C. W., Kao H. L., Li A. F., Chi C. W. & Lin W. C. Protein tyrosine-phosphatase expression profiling in gastric cancer tissues. Cancer Lett. 242, 95–103 (2006). 16338072 10.1016/j.canlet.2005.10.046

[b37] Makinoshima H. *et al.* PTPRZ1 regulates calmodulin phosphorylation and tumor progression in small-cell lung carcinoma. BMC Cancer 10.1186/1471-2407-12-537 (2012). PMC3577502 23170925

[b38] Keller S. *et al.* High-precision isothermal titration calorimetry with automated peak-shape analysis. Anal. Chem. 84, 5066–5073 (2012). 22530732 10.1021/ac3007522 PMC3389189

[b39] Otwinowski Z. & Minor W. DENZO and SCALEPACK. *Int. Tables Crystallogr*. F 11, 226–235 (2011).

[b40] Navaza J. *AMoRe*: an automated package for molecular replacement. Acta Crystallogr. A 50, 157–163 (1994).

[b41] Emsley P., Lohkamp B., Scott W. G. & Cowtan K. Features and development of Coot. Acta Crystallogr. D 66, 486–501 (2010). 20383002 10.1107/S0907444910007493 PMC2852313

[b42] Murshudov G. N. *et al.* REFMAC5 for the refinement of macromolecular crystal structures. Acta Crystallogr. D 67, 355–367 (2011). 21460454 10.1107/S0907444911001314 PMC3069751

[b43] Banks J. L. *et al.* Integrated modeling program, applied chemical theory (IMPACT). J. Comp. Chem. 26, 1752–1780 (2005). 16211539 10.1002/jcc.20292 PMC2742605

[b44] Verdonk M. L., Cole J. C., Hartshorn M. J., Murray C. W. & Taylor R. D. Improved protein-ligand docking using GOLD. Proteins 52, 609–623 (2003). 12910460 10.1002/prot.10465

[b45] Laskowski R. A. & Swindells M. B. LigPlot^+^: Multiple ligand-protein interaction diagrams for drug discovery. J. Chem. Inf. Model 51, 2778–2786 (2011). 21919503 10.1021/ci200227u

[b46] McDonald I. K. & Thornton J. M. Satisfying hydrogen bonding potential in proteins. J. Mol. Biol. 238, 777–793 (1994). 8182748 10.1006/jmbi.1994.1334

[b47] Houde D., Berkowitz S. A. & Engen J. R. The utility of hydrogen/deuterium exchange mass spectrometry in biopharmaceutical comparability studies. J. Pharm. Sci. 100, 2071–2086 (2011). 21491437 10.1002/jps.22432 PMC3164548

[b48] Houtman J. C. *et al.* Studying multisite binary and ternary protein interactions by global analysis of isothermal titration calorimetry data in SEDPHAT: application to adaptor protein complexes in cell signaling. Protein Sci. 16, 30–42 (2007). 17192587 10.1110/ps.062558507 PMC1794685

