# Supplementary material for: Small-molecule inhibition of PTPRZ reduces tumor growth in a rat model of glioblastoma
Source: Sci Rep. 2016 Feb 9;6:20473. doi: 10.1038/srep20473 (PMC4746629; doi:10.1038/srep20473)

Supplementary Information

Small-molecule inhibition of PTPRZ reduces tumor growth in a rat model of glioblastoma

Akihiro Fujikawa,[1] Asako Nagahira,[2] Hajime Sugawara,[2] Kentaro Ishii,[3] Seiichi Imajo,[2] Masahito Matsumoto,[1] Kazuya Kuboyama,[1] Ryoko Suzuki,[1] Naomi Tanga,[1,4] Masanori Noda,[5] Susumu Uchiyama,[3,5] Toshiyuki Tomoo,[2] Atsuto Ogata,[2] Makoto Masumura,[2] and Masaharu Noda[1,4*]

[1]Division of Molecular Neurobiology, National Institute for Basic Biology (NIBB), 5-1 Higashiyama, Myodaiji-cho, Okazaki, Aichi 444-8787, Japan

[2]Asubio Pharma Co., Ltd., 6-4-3 Minatojima-Minamimachi, Chuo-ku, Kobe, Hyogo, 650-0047, Japan

[3]Department of Bioorganization Research, Okazaki Institute for Integrative Bioscience, 5-1 Higashiyama, Myodaiji-cho, Okazaki, Aichi 444-8787, Japan

[4]School of Life Science, The Graduate University for Advanced Studies (SOKENDAI), 5-1 Higashiyama, Myodaiji-cho, Okazaki, Aichi 444-8787, Japan

[5]Department of Biotechnology, Graduate School of Engineering, Osaka University, 2-1 Yamadaoka, Suita, Osaka 565-0871, Japan

*Corresponding author

E-mail: madon@nibb.ac.jp

Materials and Methods

Peptide. The phosphocoumaryl-aminopropionic acid (pCAP, a fluorogenic mimic of phosphotyrosine) and pCAP-containing peptide was synthesized as described[23]. The peptide sequence prepared was as follows: pCAP-GIT1$_{549\text{-}556}$, Ac-Glu-Asp-Asp-Ala-Ile-pCAP-Ser-Val-NH_2 (where the N-terminal amino group was acetylated and C-terminal carboxyl group was amidated). The peptide was purified by reverse phase high performance liquid chromatography (HPLC) to >90% purity, and stored as 10 mM stock solutions in dimethyl sulfoxide (DMSO) at -30°C.

Expression plasmids. The full-length cDNAs of the human PTPRZ-B isoform (GenBank accession no. NM_001206839), PTPRA (M34668), PTPRM (X58288), PTPRG (NM_002841), PTPRS (NM_002850), PTPN1 (NM_002827), and PTPN6 (NM_002831) were obtained by reverse transcription-PCR (RT-PCR) from a commercial human total RNA (Sawady Technology), which were then subcloned into a mammalian expression vector, pZeoSV2(+) (Invitrogen). The entire intracellular region of human PTPRZ (ICR, amino acid residues 1698-2315, numbered from the full length PTPRZ-A isoform, M93426) and PTP-D1 (1698-2000) of human PTPRZ, PTPRA-ICR (218-807), and PTPRM-ICR (765-1452) were amplified from the full-length clones, and their cDNA inserts were subcloned into the baculovirous transfer vector, pBm050 to produce recombinant enzymes using a silkworm-baculovirous gene expression system (ref. S1). The cDNA regions of PTPRZ-ICR, PTPRG-ICR (790-1445), PTPRS-ICR (1304-1948), PTPN1 catalytic domain (1-321), and PTPN6 catalytic domain (220-523) were amplified from the respective full-length clones, and the cDNA fragments were fused in frame to the coding sequence of glutathione S-transferase (GST) of the *Escherichia coli* (*E. coli*) expression vector, pGEX-6P (GE Healthcare). The expression plasmid used for GST-fused PTPRB-ICR (mouse) was described previously (ref. S2). To generate point mutants, pGEX-6P-PTPRZ-ICR was used as a template with a Quikchange multisite-directed mutagenesis kit (Stratagene).

The mammalian expression plasmids of pEGFP-PtprzICR (WT) and pEGFP-PtprzICR (CS) for the expression of EGFP-fused rat PTPRZ-ICR (wild type

or its PTP inactive CS mutant) proteins were generated by an in-frame insertion of its corresponding cDNA (ref. 23) into the pEYFP-C1 vector (Clontech). Predesigned mouse/rat *Ptprz* shRNA plasmid DNA (TRC number: TRCN0000081069) was purchased from Sigma-Aldrich.

Recombinant proteins. The pBm050 transfer vector of human PTPRZ-ICR, PTPRZ-D1, PTPRA-ICR, or PTPRM-ICR was co-transfected with *Bombyx mori* nucleopolyhedrovirus (BmNPV; CPd strain) genomic DNA into cells derived from *Bombyx mori*. After being incubated for 6 days, the recombinant virus was recovered and injected into the body of silkworm pupae. The hemolymph was collected 6 days after the virus injection. Recombinant proteins were expressed as an N-terminal FLAG-tagged form with a HRV 3C protease cleavage sequence between the FLAG sequence and target protein. FLAG-tagged proteins in hemolymph tissues were trapped on anti-FLAG-immobilized beads (Sigma-Aldrich). The target proteins were selectively eluted by on-column digestion using HRV 3C Protease (Novagen), and further purified by ion exchange chromatography on HiTrap Q (GE Healthcare), and gel filtration on HiLoad 16/600 Superdex 200pg (GE Healthcare). The purities of the proteins thus obtained were over 95% as determined by SDS-PAGE, and their molecular sizes were verified by mass spectrometry. The other recombinant enzymes (PTPRG, PTPRS, PTPRB, PTPN1, PTPN6, and PTPRZ mutants) were expressed as GST-fused proteins in *E. coli* strain BL21, and purified by glutathione affinity chromatography. These recombinant proteins were used to determine IC_{50} values. .

Compound screening and kinetic analysis. We miniaturized the assay volume for a 384-well platform and adjusted the assay conditions in order to improve the sensitivity and reproducibility of the inhibitor screen. The assay buffer was changed to 20 mM Hepes, pH 7.0, containing 50 mM NaCl, 5 mM DTT, 0.01% (*v*/*v*) Brij-35, and 0.2% (*w*/*v*) BSA. A mixed lot of rat PTPRZ-ICR was used for the initial screening by diluting to an appropriate concentration. In a 384-well plate, the enzyme solution (13 μl) was mixed with each compound (7 μl) in the assay buffer, and preincubated for 15 min at room temperature. The reaction was

initiated by the addition of pCAP-GIT1$_{549-556}$ (5 μl, 100 μM) and terminated after 90 min with 1 M Tris HCl, pH 9.2 (20 μl). The final concentrations of compounds, the pCAP substrate, and DMSO were 10 μg/ml, 20 μM, and 1% (*v*/*v*), respectively. Fluorescence was measured at excitation/emission wavelengths of 380/460 nm using an EnVision Multilabel Reader (PerkinElmer). A decrease of more than 40% in the signal intensity against the DMSO control was considered to be a potential hit, and reproducibility was examined with a freshly prepared stock.

Kinetic analyses of SCB4380 inhibition was performed in the three-component buffer at pH 7.0 using a 384-well plate. The reaction was initiated by the addition of the mixture of SCB4380 and appropriate pCAP-peptide substrates into the enzyme (human PTPRZ-ICR) solution. IC$_{50}$ value was calculated by nonlinear regression (JMP software, SAS Institute). The inhibition type was analyzed by a Lineweaver-Burk plot according to Michaelis-Menten kinetics.

Selectivity of SCB4380 for PTP family members. Assays were performed in the reaction buffer (the three-component buffer at pH 6.5 containing 100 μg/ml BSA, 5 mM DTT and 0.01% Brij-35) with DiFMUP (6,8-difluoro-4-methylumbiliferyl phosphate, Molecular probe) as a nonspecific PTP substrate. One hundred microliters of 40 μM DiFMUP were preincubated in a cuvette at room temperature. The reaction was initiated by adding 100 μl of the mixture of SCB4380 and an enzyme solution; the amount of the enzyme was adjusted to give a convenient rate. In each experiment, the hydrolysis of DiFMUP was continuously monitored for ~100 s as an increase in fluorescence at 455 nm (excitation at 358 nm) using a spectrofluorometer (FI-4500, Hitachi). We measured the slope of the linear increase of the fluorescence signal as an indicator of catalytic activity. IC$_{50}$ values were determined as above.

Cell culture and DNA transfection. Rat C6 glioblastoma cells were maintained with Dulbecco's modified Eagle's medium (DMEM, cat no. 11995-065, Life Technologies) supplemented with 10% fetal bovine serum (FBS) and 100 U/ml penicillin–streptomycin (cat no. 15140-122, Invitrogen) in a humidified incubator

at 37°C with 5% CO_2. Cells (2 × 10^6 cells) were electroporated with plasmid (1 μg) or siRNA (100 pmol) using the Amaxa Nucleofector (Amaxa) according to the manufacturer's protocol. After a 24- to 36-h culture, cells were used for migration and proliferation assays. C6 cell lines stably expressing shRNA against PTPRZ were selected by limited dilution in the presence of 5 μg/ml of puromycin.

Microscopic observation of SCB4380 incorporation in cells. C6 cells (1.5 × 10^4 cells per well) were plated on a 24-well plastic tissue culture plate and cultured with 500 μl of DMEM supplemented with 10% FBS for microscopic observation. The next day, the medium was changed to 500 μl of Opti-MEM, and 100 μl of the compound solution was added as indicated. Cells were then incubated for 3 h at 37°C. After washing, cells were immediately observed using the 40× objective of a fluorescence microscope (BZ-8000, Keyence) with a filter for TexasRed (excitation wavelength, 560/40 nm; absorption wavelength, 630/60 nm; dichroic mirror wavelength, 595 nm).

Boyden chamber assay. Boyden chamber assays were performed in the following manner using transwell inserts with an 8-μm pore-sized membrane (Chemotaxicell, Kurabo) and 24-well plastic tissue culture plates (BD Falcon). Cells in 250 μl of the Opti-MEM medium containing 100 μg/ml BSA were transferred onto a laminin (10 μg/ml)-coated transwell insert (1.5 × 10^5 cells per well). The lower chambers were filled with 200 μl of the medium containing epidermal growth factor (EGF, 10 ng/ml) as a chemoattractant. After a 3-h incubation, cells were fixed with 10% neutral formalin and stained with DAPI (4', 6-diamidino-2-phenylindole). Cells present in the upper chamber were removed with cotton swabs, and the number of cells that migrated to the lower surface was counted under a conventional fluorescence microscope. The cell treatment with the SCB4380/liposome complex was carried out as follows. Parental or *Ptprz*-knockdown C6 cells suspended in the Opti-MEM medium at 6 × 10^5 cells per ml were mixed with a one-fifth volume of a SCB4380/liposome solution that was diluted at the desired concentration with the same medium. After a 1-h

incubation at 37°C, the cell suspension was directly added to transwell inserts and then allowed to migrate for 2 h.

Cell proliferation assay. Cells were plated on a 24-well plastic tissue culture plate (1.5×10^4 cells per well), and cultured with 500 µl of DMEM supplemented with 10% FBS. The cultured cells were harvested at the indicated time points by trypsinization, and manually counted with a hemacytometer. In order to treat cells with SCB4380 (or compound 2), medium was changed to 500 µl of Opti-MEM supplemented with 2% FBS at 1 h after plating, and 100 µl of the SCB4380 (or compound 2)/liposome complex diluted to the desired concentration with the same Opti-MEM medium was added to each well.

Reverse transcription (RT)-PCR analyses. Total RNA of cultured cells was isolated with a TRIzol Reagent kit (Invitrogen), and cDNAs were synthesized using the SuperScript III Reverse Transcriptase kit (Invitrogen) with oligo (dT) primers. Specific cDNA regions for the Ptprz, Ptprg, and glyceraldehyde-3-phosphate dehydrogenase (GAPDH, used as a control) were then amplified by PCR with the following primer sets: *Ptprz*, forward 5'-cctccagaccacttgatttg-3' and reverse 5'-ttttcagcaagttgtgtgag-3'; *Ptprg*, forward 5- cagtattggccaacggagaacact-3' and reverse 5'- gagaatggcttccaacaaggcatc-3'; or *GAPDH*, forward 5'- ggatttggccgtatcggacgcctggttacc -3' and reverse 5'- tcttctgagtggcagtgatggcatggactg -3'. The relative quantity of the target mRNA was normalized to GAPDH.

Immunoprecipitation and Western blotting. Cells were extracted with lysis buffer containing 20 mM Tris-HCl, pH 7.4, 1% NP-40, 150 mM NaCl, 10 mM NaF, 1 mM sodium orthovanadate, and an EDTA-free protease inhibitor cocktail (complete EDTA-free, Roche). The supernatants, which were preabsorbed with protein G Sepharose (GE Healthcare) for 30 min at 4°C, were incubated with Protein G Sepharose coated with a mouse anti-paxillin antibody (BD Bioscience) for 1 h at 4°C. The immunocomplexes formed were boiled with SDS-PAGE sample buffer, and eluted proteins were subjected to SDS-PAGE followed by semi-dry electroblotting onto a polyvinylidene difluoride membrane. The membrane was

blocked with a blocking solution (4% non-fat dry milk and 0.1% Triton X-100 in 10 mM Tris-HCl, pH 7.4, 150 mM NaCl) and incubated overnight with the respective antibodies in the blocking solution. The binding of these antibodies was detected using an ECL Western blotting detection reagent (GE Healthcare). In order to detect phosphorylated proteins, the membranes were blocked with 1% BSA and 0.1% Triton X-100 in 10 mM Tris-HCl, pH 7.4, and 150 mM NaCl, and then incubated with the HRP-conjugated anti-phospho-Tyr mAb (clone PY20) or mouse anti-pY118-paxillin antibody (BD Bioscience).

Legends to Supplementary Figures and Table

Supplementary Figure S1. Predominant expression of PTPRZ-B and its proteolytic intracellular fragment in C6 glioblastoma cells. (**A**) Schematic representation of PTPRZ-B and its proteolytic fragments. Z_B-ECF (the extracellular fragment of PTPRZ-B) and ZΔE (the membrane-tethered fragment) were generated by metalloproteinase-mediated cleavage, and ZΔE was further processed to the intracellular fragment (Z-ICF) by PS/γ-secretase[21]. Domains are highlighted in different colors: CAH (red), carbonic anhydrase-like domain; FNIII (blue), fibronectin type III domain; PTP-D1 (orange, active PTP) and PTP-D2 (green, inactive PTP), protein tyrosine phosphatase domain. Regions corresponding to the epitopes of antibodies used in this study are indicated by vertical lines. (**B**) PTPRZ expression in rat C6 glioblastoma cells. C6 cells were cultured under usual conditions. Cell extracts (the left two panels) and culture medium (the right panel) were treated with (+) or without (-) chondroitinase ABC (chABC) in order to examine chondroitin sulfate modifications, and were then analyzed by Western blotting using anti-RPTPβ, which recognizes the intracellular C-terminal region of PTPRZ, or anti-PTPRZ-S, which recognizes the extracellular region, as shown in **A**. The chABC treatment scarcely affected the Western blot visualization of PTPRZ-B as described (ref. S3) or Z_B-ECF, indicating that PTPRZ-B expressed in C6 cells was the non-proteoglycan form. On the other hand, PTPRZ-S was slightly modified with chondroitin sulfate, but less so than that in the normal brain[21]. (**C**) Metalloproteinase-mediated ectodomain shedding of PTPRZ-B in C6 cells. C6 cells were washed once and incubated with or without PMA for 1 h in fresh serum-free medium, to which GM6001 was added 20 min before the PMA stimulation. Cell extracts (upper panel) and conditioned media (the lower panel) were analyzed by Western blotting using anti-PTPRZ-S without the chABC treatment. When C6 cells were treated with the tumor-promoting phorbol ester 12-myristate 13-acetate (PMA), which has been shown to stimulate metalloproteinase-mediated PTPRZ processing[21], the 180-kDa band in the medium was increased, and PTPRZ-B in cell extracts inversely decreased. These effects

were completely inhibited with a broad-spectrum metalloproteinase inhibitor, GM6001.

Supplementary Figure S2. Effects of *Ptprz* or *Ptprg* knockdown in C6 glioblastoma cells. (A) RT-PCR. (B) Boyden chamber assay. C6 cells (2 × 10^6 cells) were electroporated with 100 pmol siRNA (for rat *Ptprz*, siRNA ID; SASI_Rn01_00053281: for rat *Ptprg* siRNA, siRNA ID; SASI_Rn01_00066571: Sigma-Aldrich) or control siRNA using the Amaxa Nucleofector (Amaxa) according to the manufacturer's protocol. After a 24- to 36-h culture, total RNAs were subjected to RT-PCR with *GAPDH* as internal control. Boyden chamber assay of cells were performed as in Figure 1B. Data are the mean ± S.E. (n = 4). *, $P < 0.05$, significantly different from the control by the Student's t-test.

Supplementary Table S1.
Data on the screening of small-molecule inhibitors for PTPRZ.

Supplementary Figure S3. Hydrogen/deuterium (H/D) exchange protection of PTPRZ-ICR by SCB4380. (A) H/D exchange MS analysis of Peptide coverage map of PTPRZ-ICR. A peptide coverage map was created by MSTools (ref. S4). A coverage map of human PTPRZ-ICR (for the full-length PTPRZ-A isoform, GenBank accession no. M93426) showing the location of the 90 common peptides both in PTPRZ-ICR alone and the PTPRZ-ICR and SCB4380 complex. **(B)** Relative deuterium incorporation curves for all PTPRZ-ICR fragments by pepsin. The amount of deuterium exchanged (%) as a function of time was measured in the absence (red) or presence (blue) of SCB4380. Peptide sequences with amino acid residue numbers are shown at the top of each figure.

Supplementary Figure S4. X-ray structure of human PTPRZ-D1. (A) Stereo view of the crystal structure around the catalytic residue, Cys1933, in the D1 of human PTPRZ. Cross-eyed stereo view of $2F_O$-F_C map around the catalytically essential residue, Cys1933, contoured at 2σ. Orientation of the

molecule is the same as Figure 7A. (**B**) Data collection and refinement statistics of X-ray structure of human PTPRZ-D1.

Supplementary Figure S5. Suppressive activity of the SCB4380/liposome complex on malignant phenotypes of C6 glioblastoma cells. (A) Direct microinjection of SCB4380 into C6 cells cultured on a glass bottom dish. A microinjection of SCB4380 was performed using a micromanipulator Femtojet B 5247 and Injectman NI 2 (Eppendorf) with a pressure of 100 hPa and time of 0.5 s. Fluorescence and bright-field (BF) images were taken from the same region before and after the injection of SCB4380, as indicated by arrow heads. This injection experiment showed that SCB4380 inside the cell exhibited red fluorescence by fluorescence excitation. (**B**) Stability of SCB4380 in C6 cells. Analysis with time-lapse microscopy (Biostation IM, Nikon) showed that the average half-life for disappearance of the fluorescent signal of SCB4380 was ~50 min. (**C**) SCB4380 uptake assay. C6 cells were suspended in Opti-MEM medium at 2×10^6 cells per ml. Five hundred microliter aliquots of the cell suspension were mixed with 100 μl of a SCB4380/liposome solution that was diluted to the desired concentration with the same medium. After a 1-h incubation at 37°C, the cells were washed five times by centrifugation (1,200 *g* for 5 min) in phosphate buffered saline (PBS, pH7.4; cat no. 10010-031, Life Technologies), and SCB4380 in the cells was extracted with 100 μl of methanol. The supernatant was collected by centrifugation (20,000 *g* for 15 min), and absorbance at 525 nm was measured with a microplate reader (SH-9000Lab, Corona Electric Co., Ltd.). (**D**) Relative cell numbers 1 h after the addition of the SCB4380/liposome complex, as shown in Figure 8C. (**E**) Representative images of the Boyden chamber assay shown in Figure 8D.

Supplementary Figure S6. No inhibitory activity of compound 2/liposome on the growth of C6 cells. Cells were counted 1 and 25 h after the addition of vehicle/liposome, SCB4380/liposome, or compound 2/liposome (the final concentration was 40 μM) and increases in 24 h were presented as relative values.

Data are the mean ± S.E. (n = 3). *, $P < 0.05$, significantly different from the vehicle/liposome-treated cells by the Student's t-test.

Supplemental References

Ref. S1. Suzuki, T. *et al.* Efficient protein production using a *Bombyx mori* nuclear polyhedrosis virus lacking the cysteine protease gene. *J. General. Virol.* **78**, 3073–3080 (1997).

Ref. S2. Sakuraba, J., Shintani, T. Tani, S. & Noda M. Substrate specificity of R3 receptor-like protein-tyrosine phosphatase subfamily toward receptor protein-tyrosine kinases. *J. Biol. Chem.* **288**, 23421-23431 (2013).

Ref. S3. Canoll, P. D., Petanceska, S., Schlessinger, J. & Musacchio, J. M. Three forms of RPTP-ß are differentially expressed during gliogenesis in the developing rat brain and during glial cell differentiation in culture. *J. Neurosci. Res.* **44**, 199–215 (1996).

Ref. S4. Kavan, D. & Man, P. MSTools-Web based application for visualization and presentation of HXMS data. *Int. J. Mass Spectrom.* **302**, 53–58 (2011).

Supplementary Figure S1

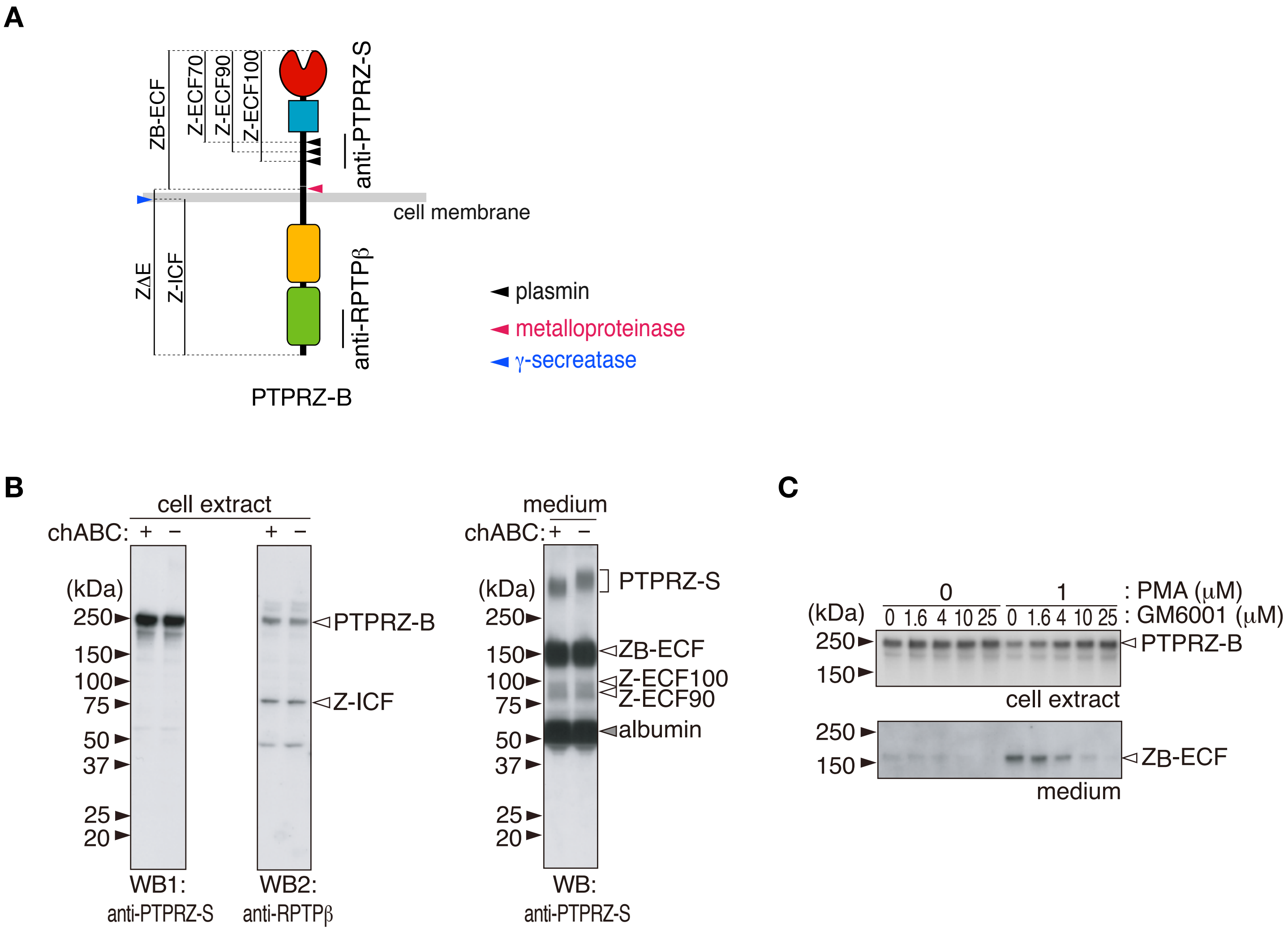

Supplementary Figure S2

A

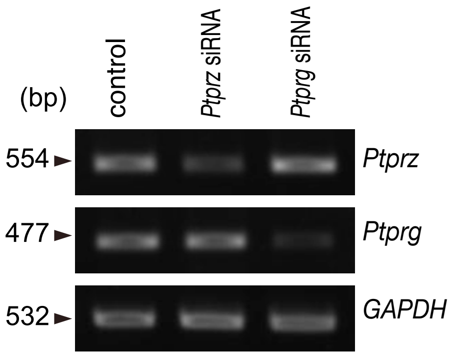

B

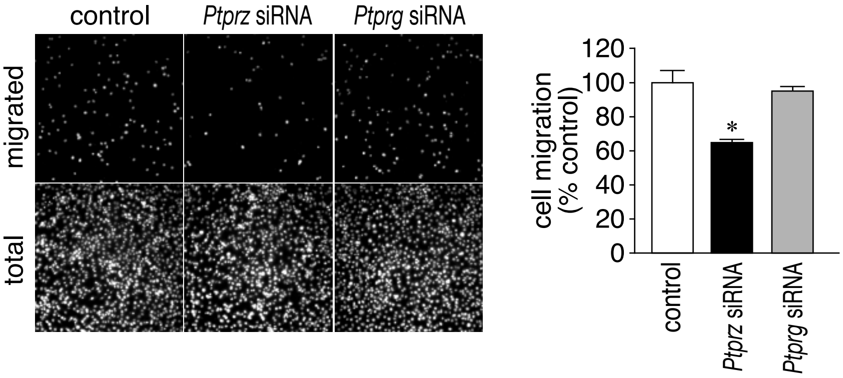

Supplementary Table S1

Small Molecule Screening Data

Category	Parameter	Description
Assay	Type of assay	*in vitro*
	Target	protein tyrosine phosphatase receptor type Z (PTPRZ)
	Primary measurement	detection of a fluorescence increase upon substrate hydrosis
	Key reagents	pCAP (phosphocoumaryl amino propionic acid) peptides, and recombinant enzyme proteins
	Assay protocol	see the section of compound screening in Online Methods
Library	Library size	c.a. 26,000 compounds
	Library composition	a set of diverse compounds including small organic molecules and drug-oriented species
	Source	Asubio Pharma Co., Ltd.
Screen	Format	384-wel plate
	Concentration(s) tested	10 µg/ml, 0.5% DMSO
	Plate controls	at room temp
	Reagent/ compound dispensing system	EDR-384UX Multi-channel Pipetter (BIOTEC)
	Detection instrument and software	EnVision® Multilabel Reader (PerkinElmer)
	Assay validation/QC	Z' score > 0.5
	Correction factors	none
	Normalization	% inhibition; 100 x [1 – (signal of compound treated well – average signal of wells without enzyme) / (average signal of DMSO control wells – average signal of wells without enzyme)]
Post-HTS analysis	Hit criteria	% inhibition > 40%
	Hit rate	a total of 32 initial hits from 26,000 compounds (0.1%) at the criteria
	Additional assay(s)	determination of IC_{50}, selectively for other PTP molecules as secondary assays
	Confirmation of hit purity and structure	compounds were repurchased (see Online Methods)

Supplementary Figure S3A

A

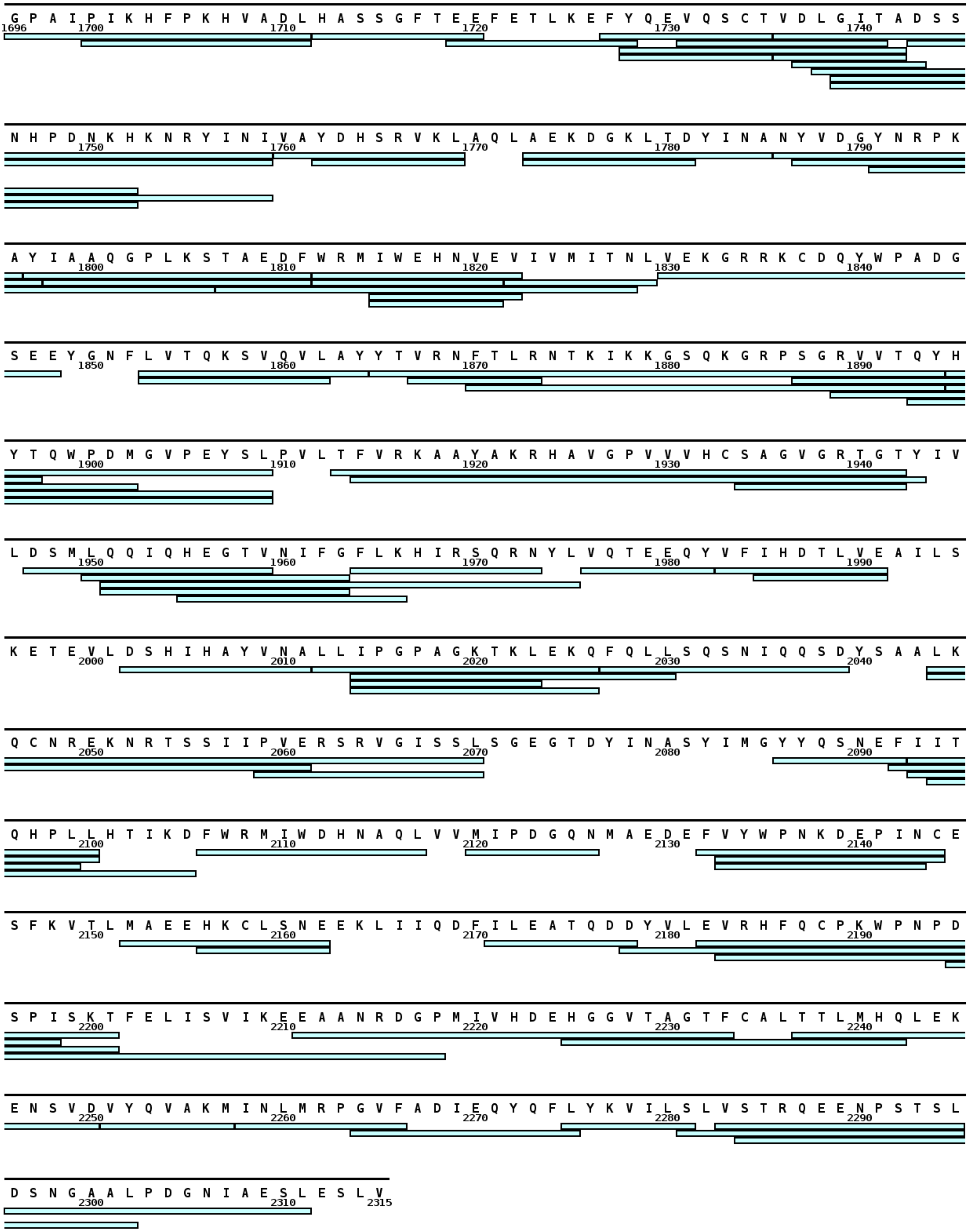

Supplementary Figure S3B

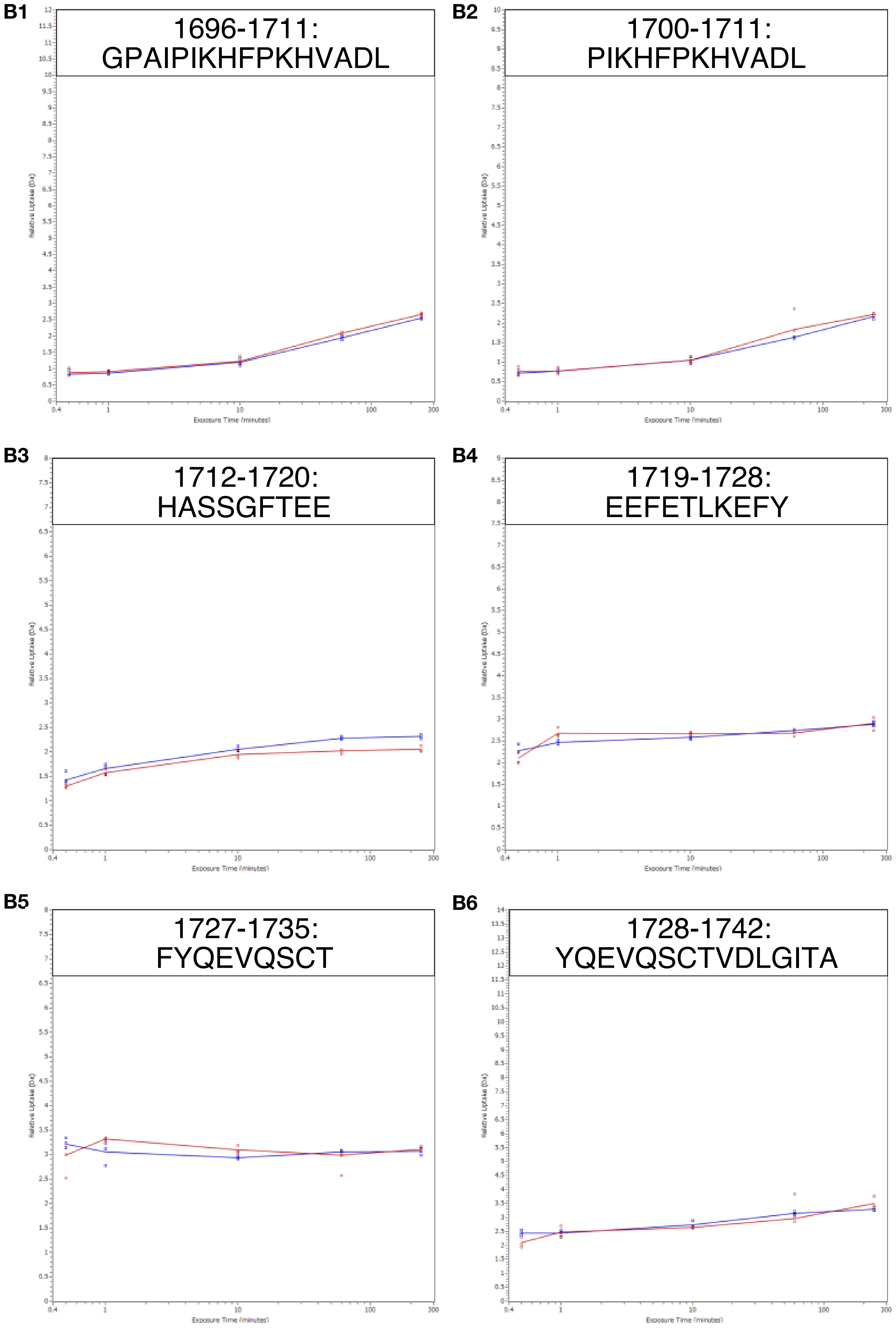

Supplementary Figure S3B

B7

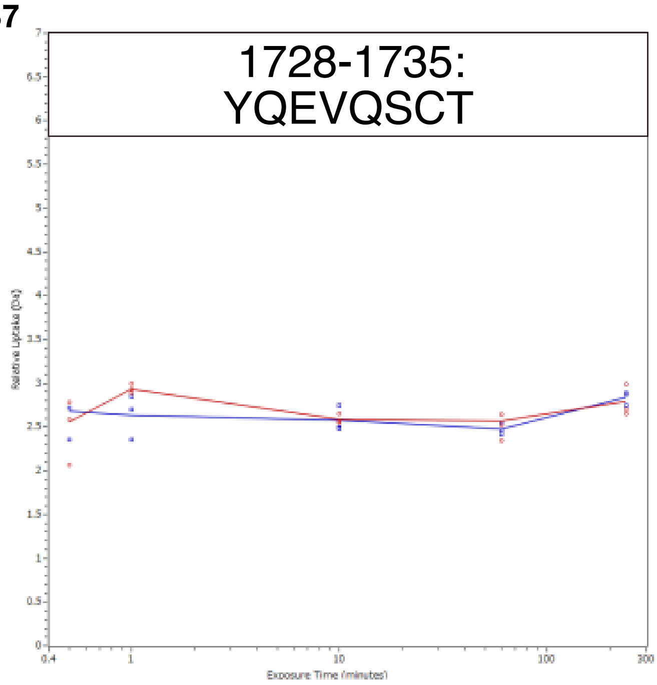

B8

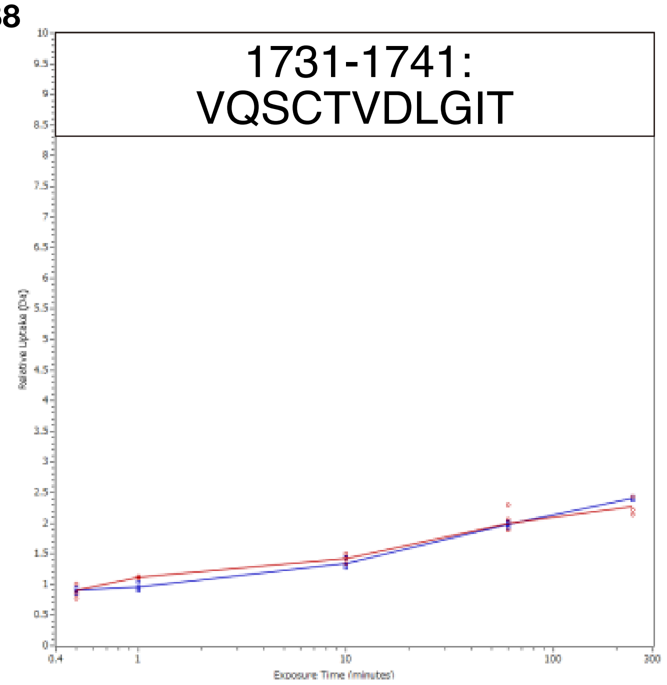

B9

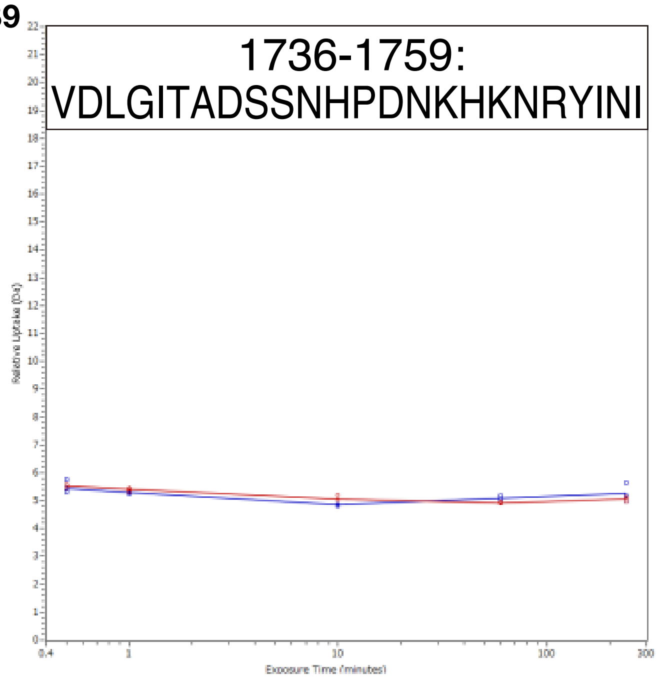

B10

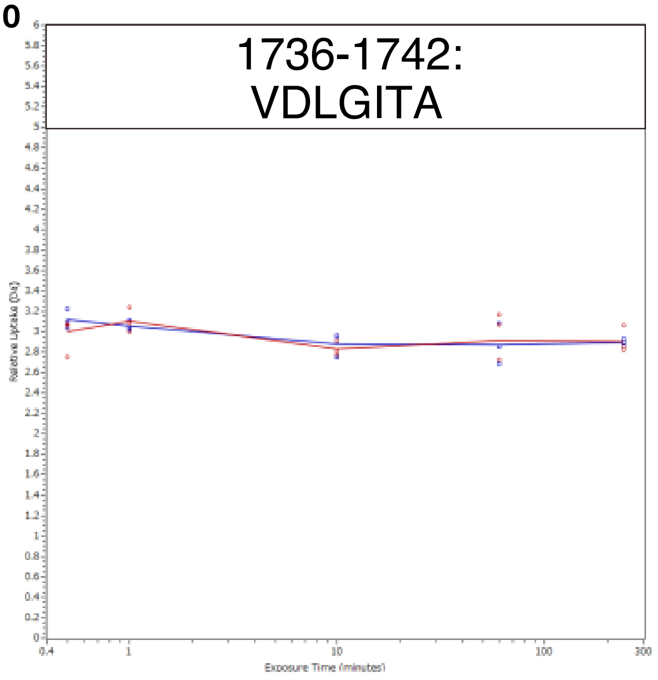

B11

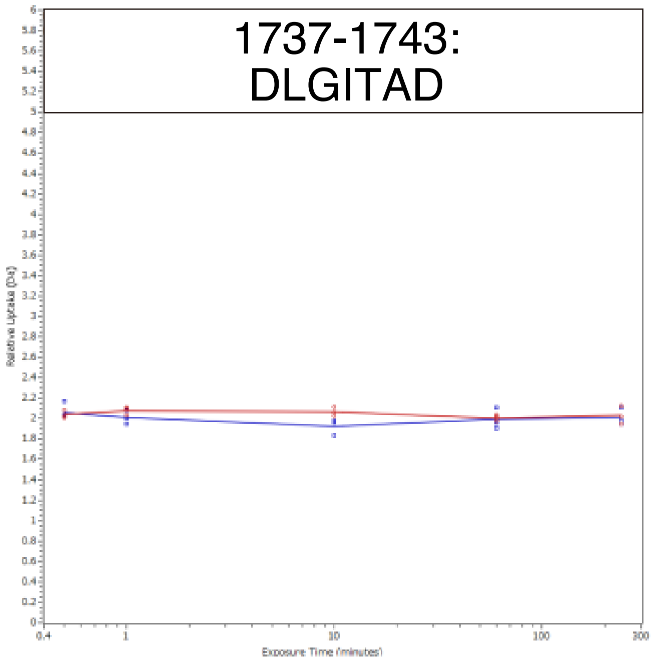

B12

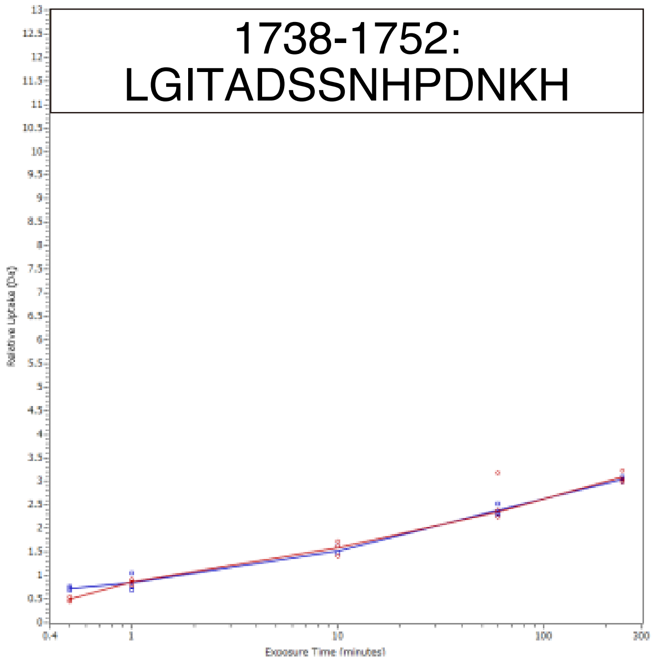

Supplementary Figure S3B

B13

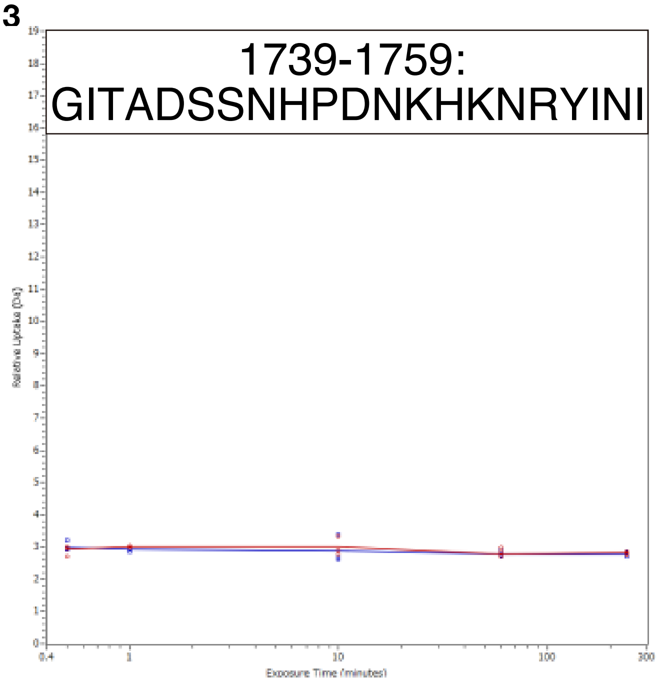

B14

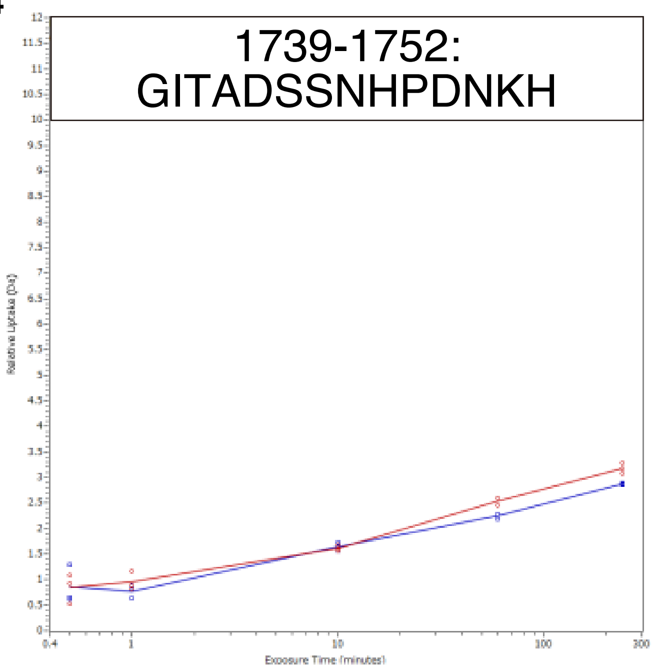

B15

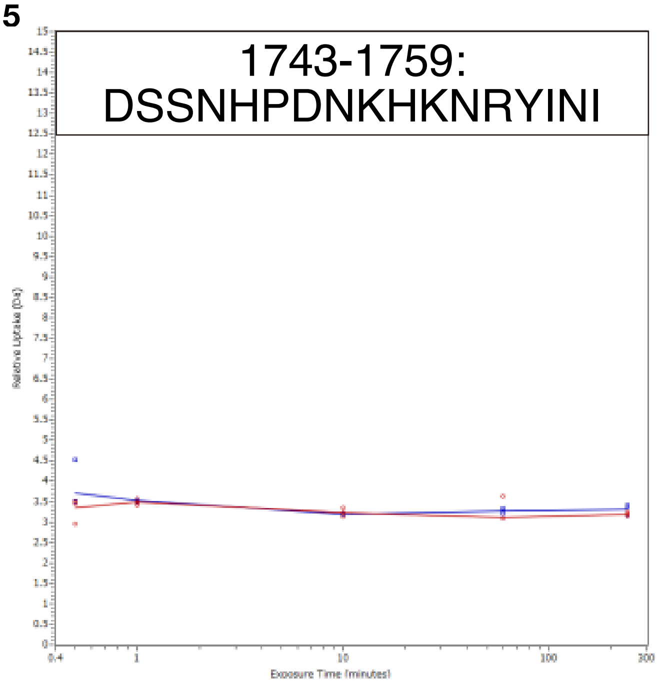

B16

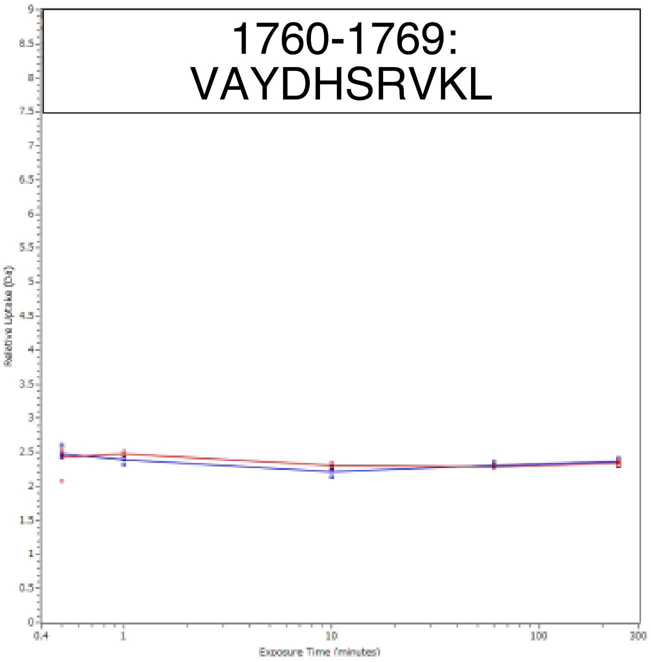

B17

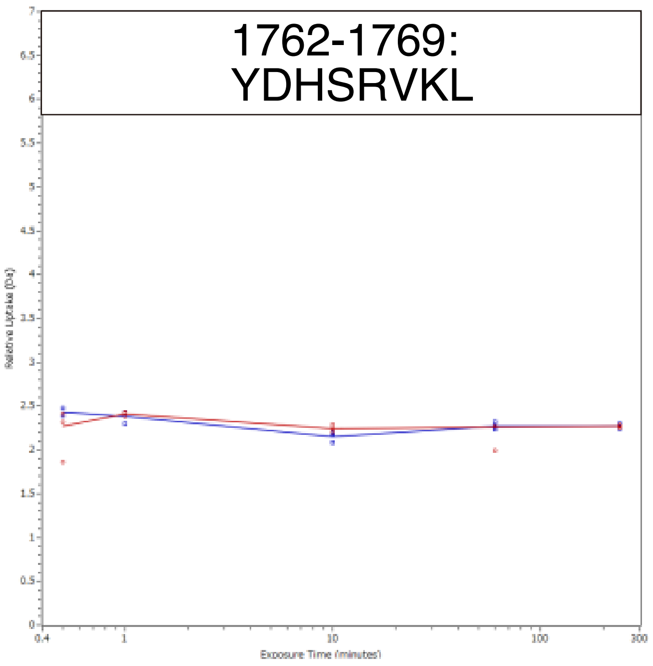

B18

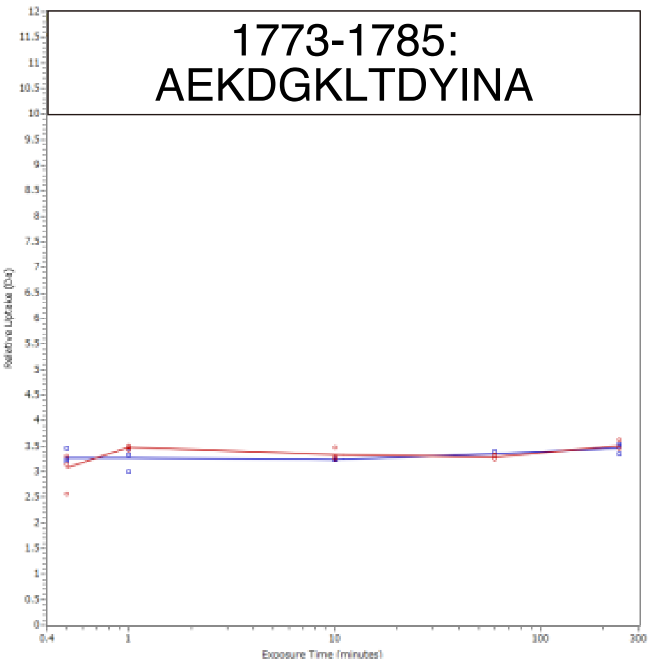

Supplementary Figure S3B

B19

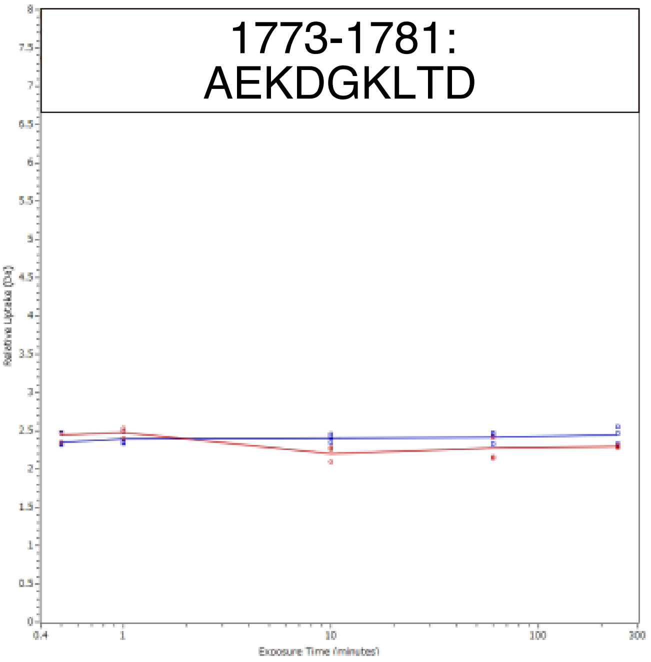

B20

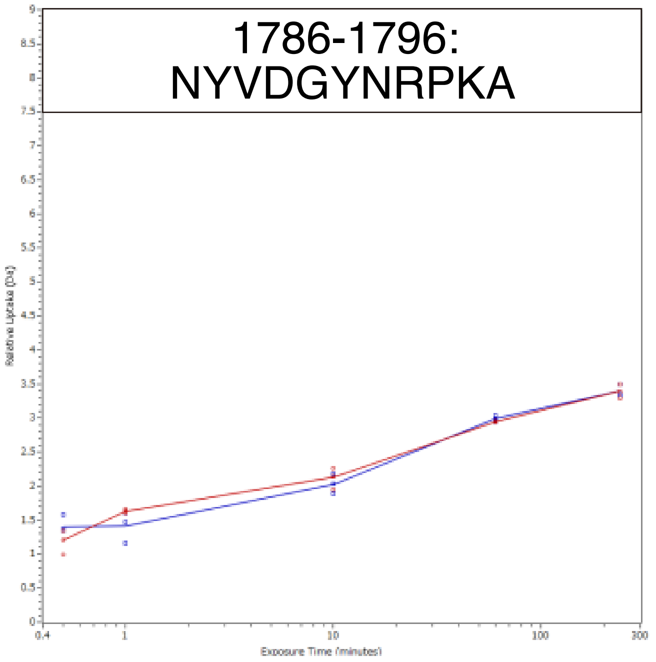

B21

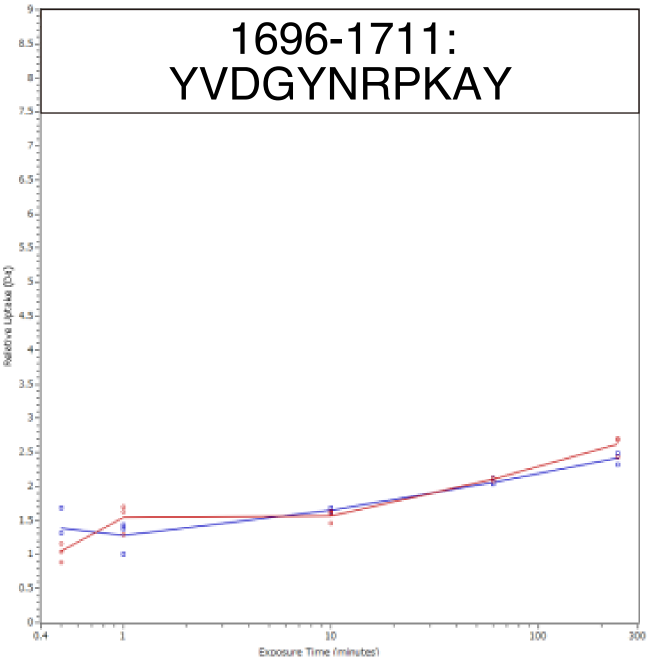

B22

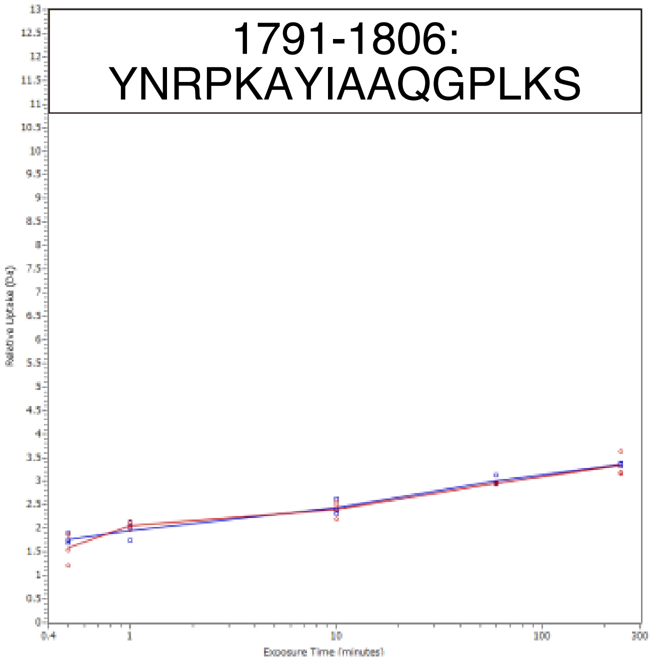

B23

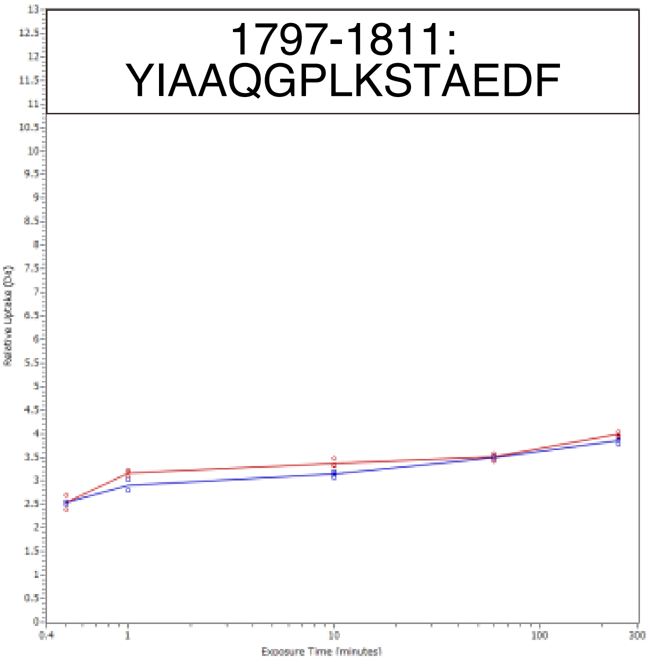

B24

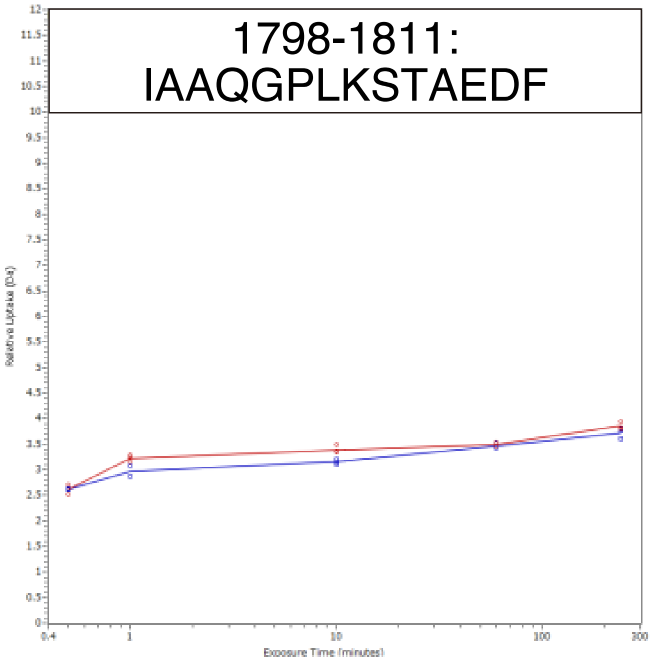

Supplementary Figure S3B

B25

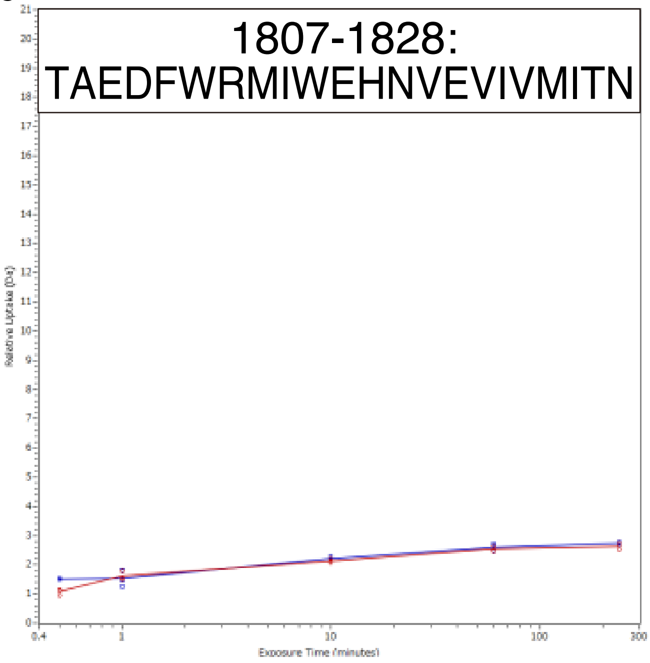

B26

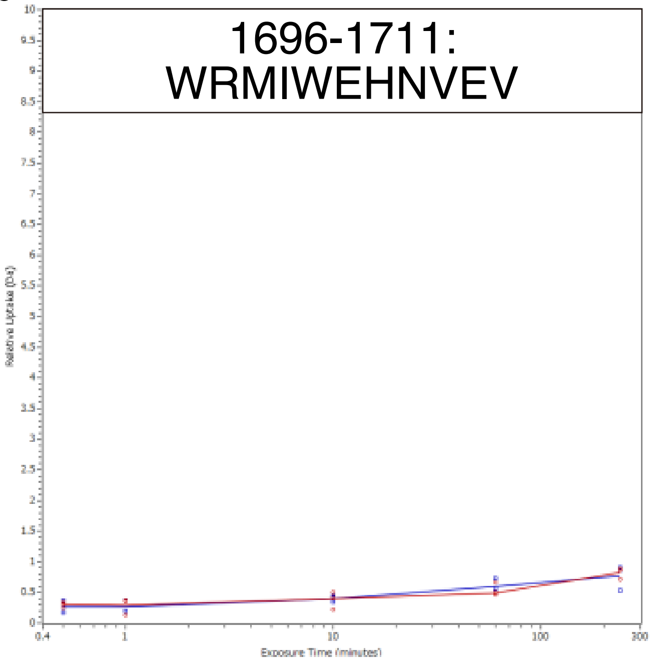

B27

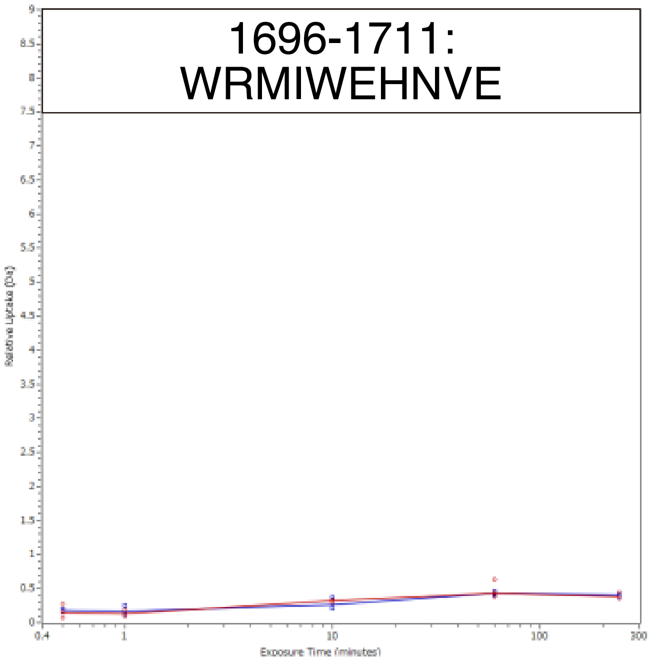

B28

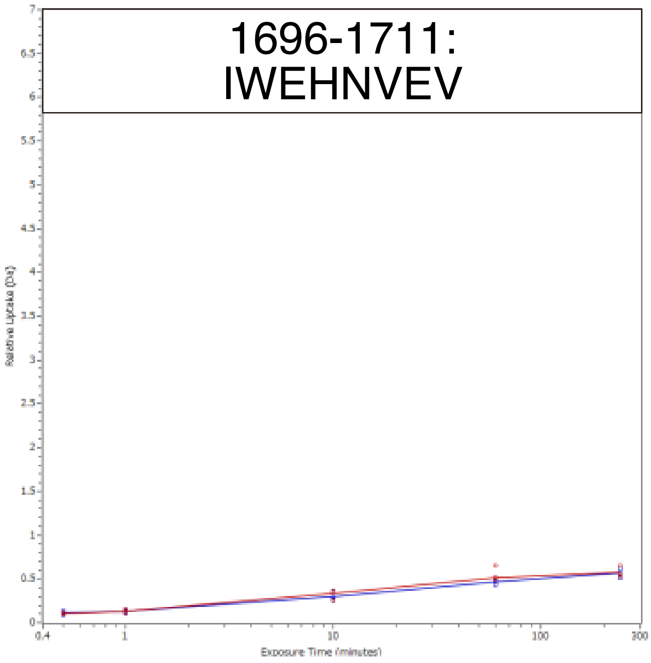

B29

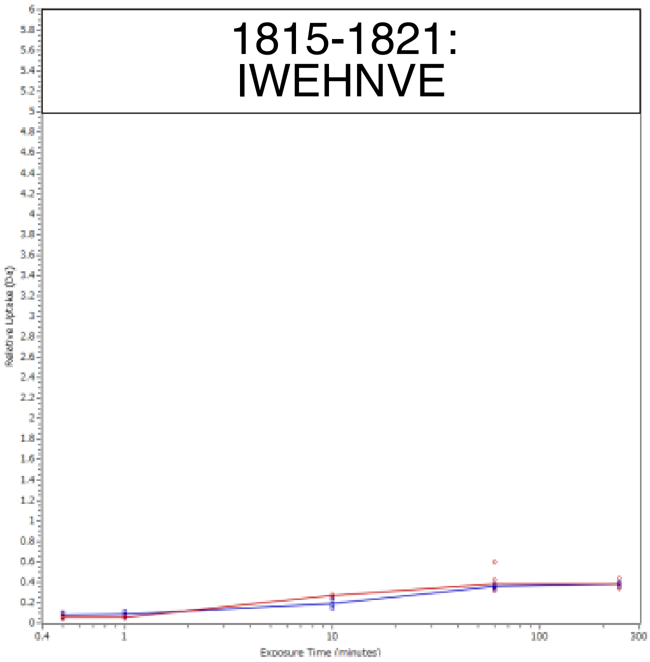

B30

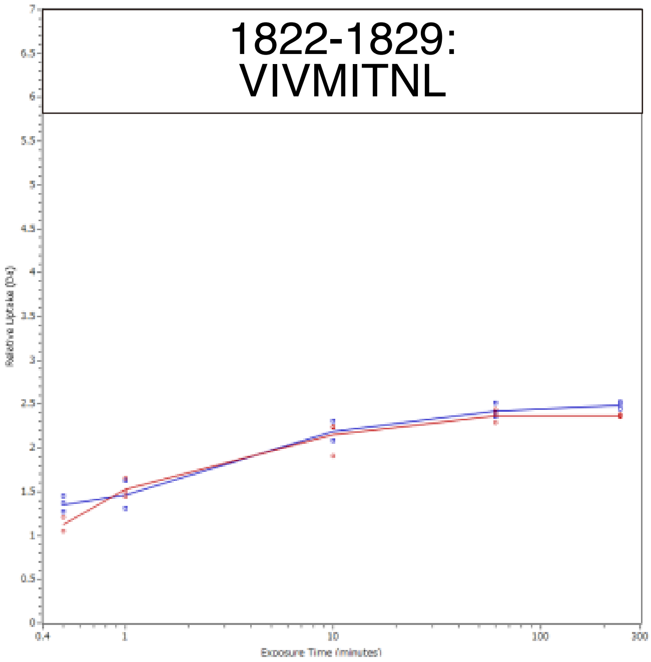

Supplementary Figure S3B

B31

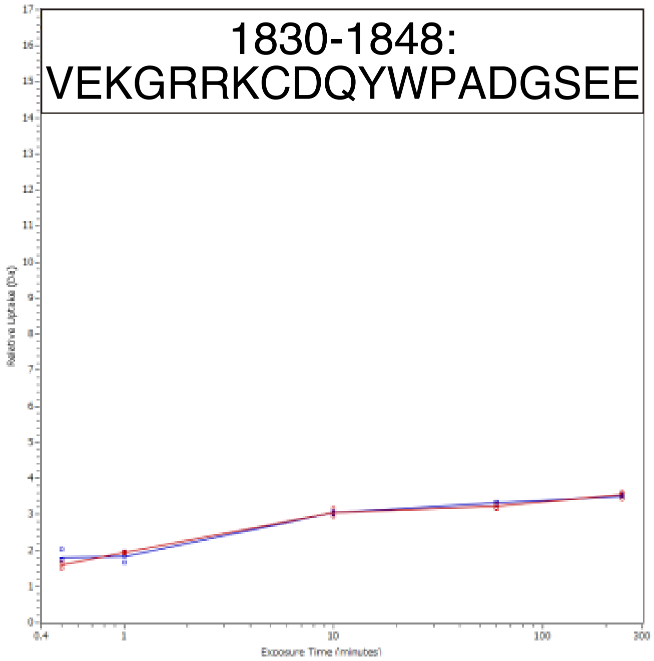

B32

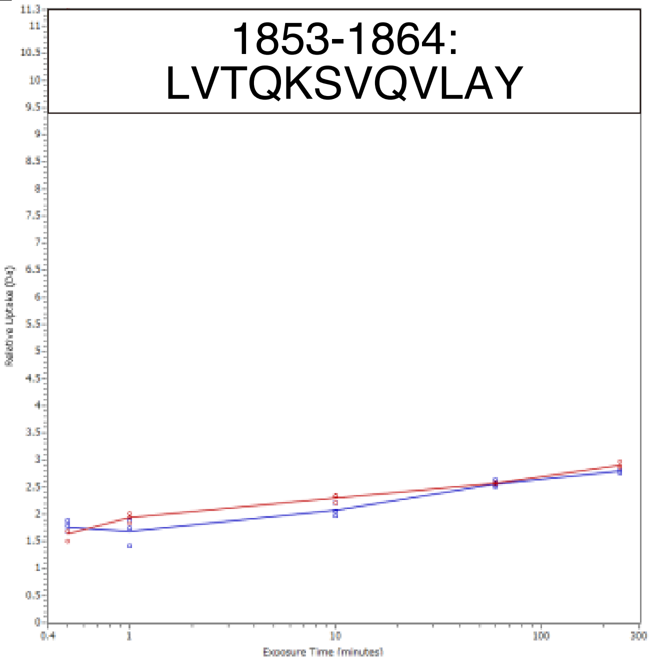

B33

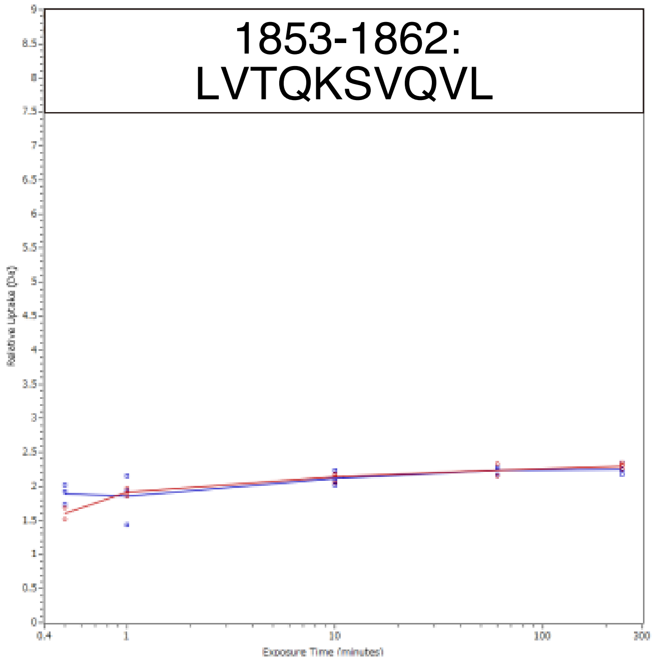

B34

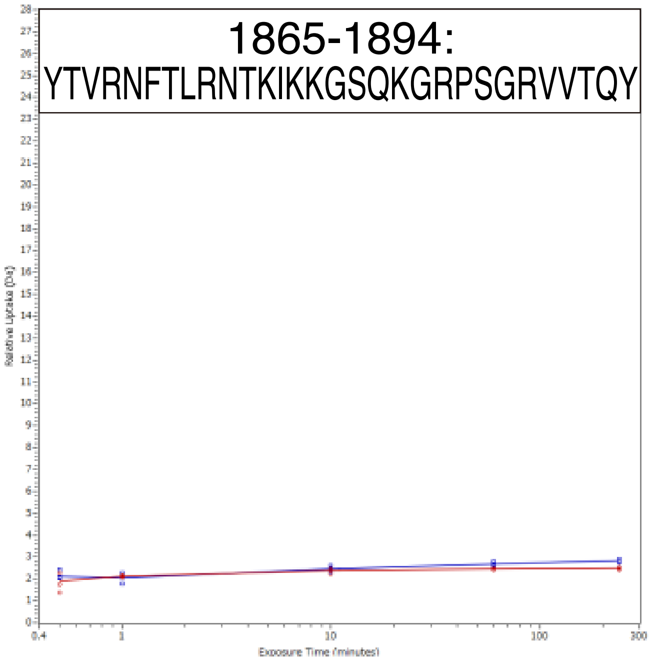

B35

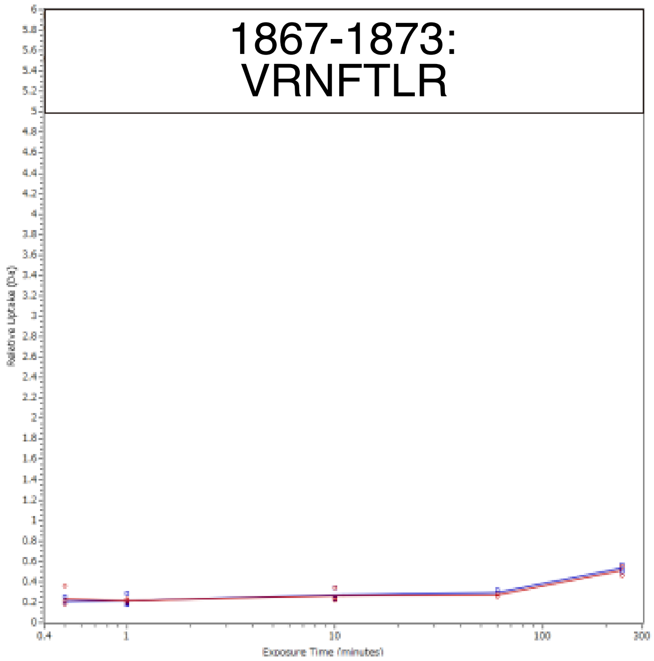

B36

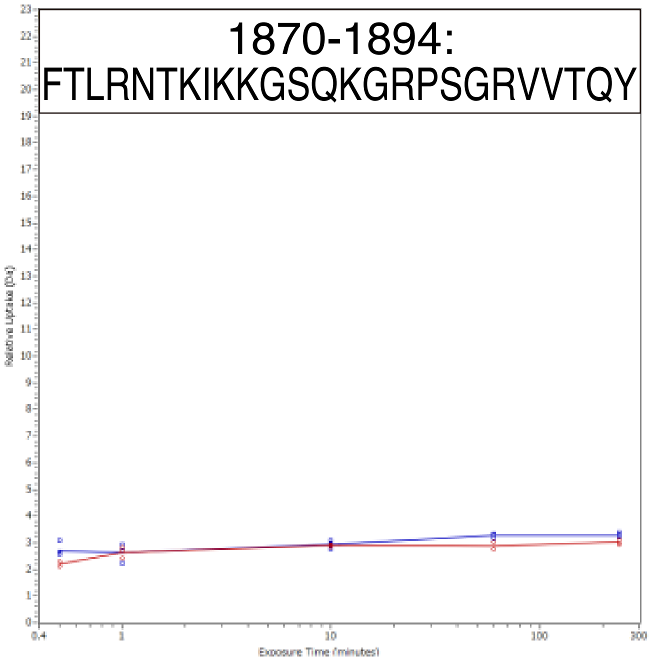

Supplementary Figure S3B

B37

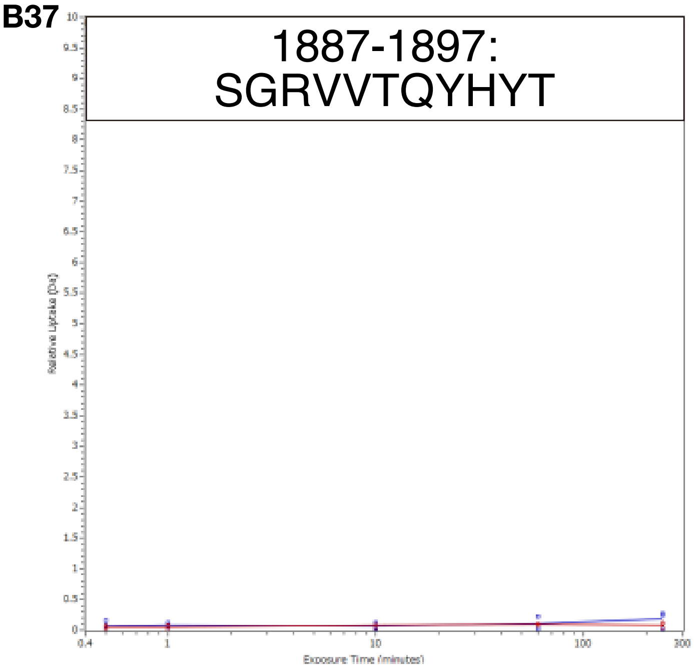

B38

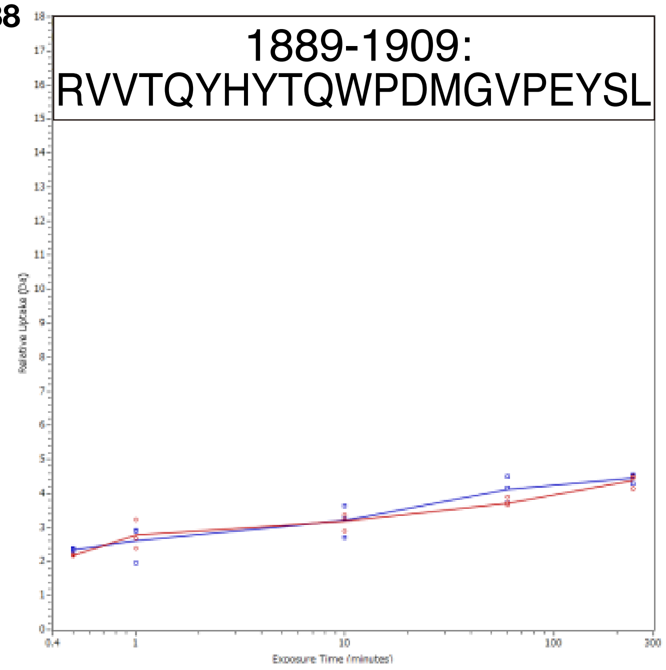

B39

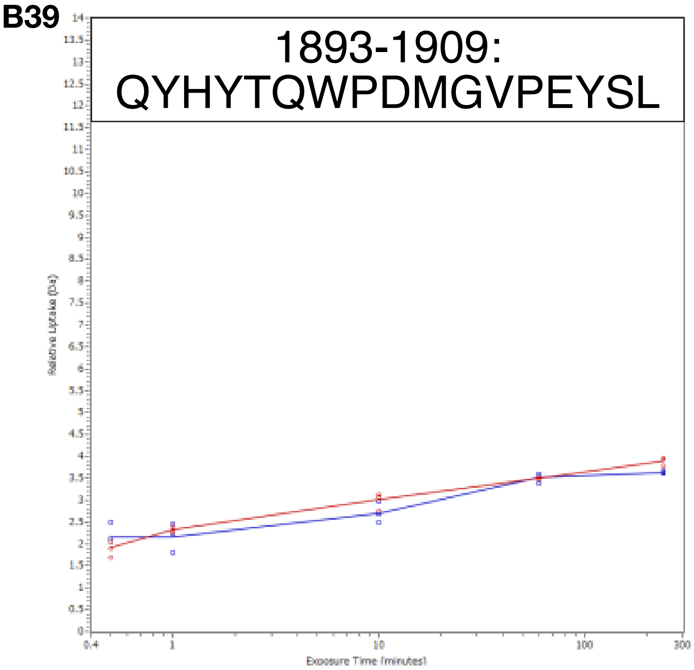

B40

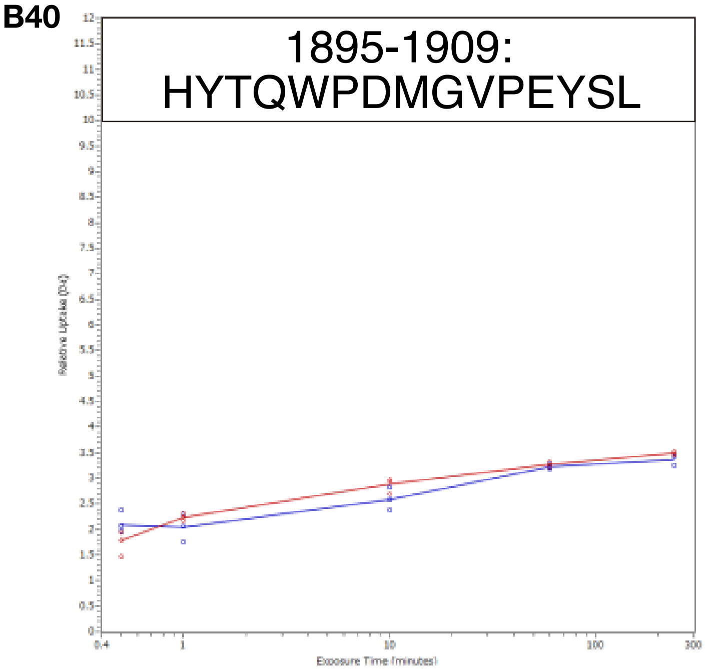

B41

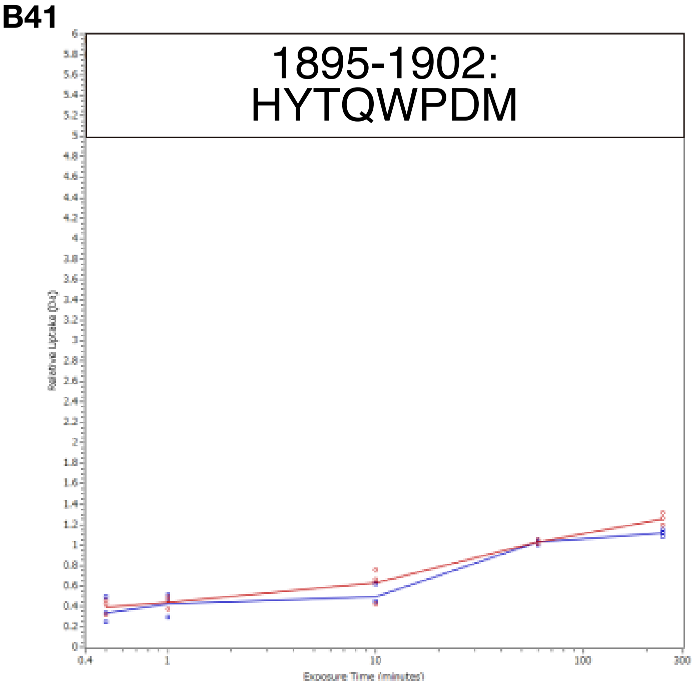

B42

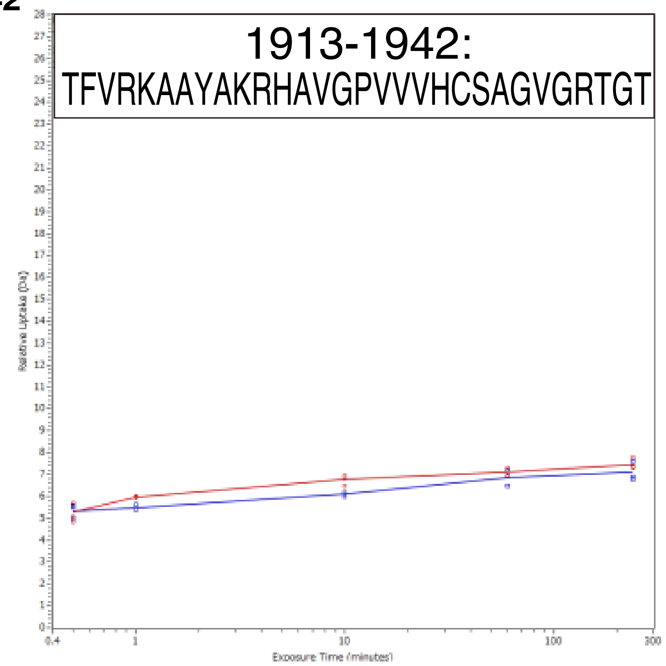

Supplementary Figure S3B

B43

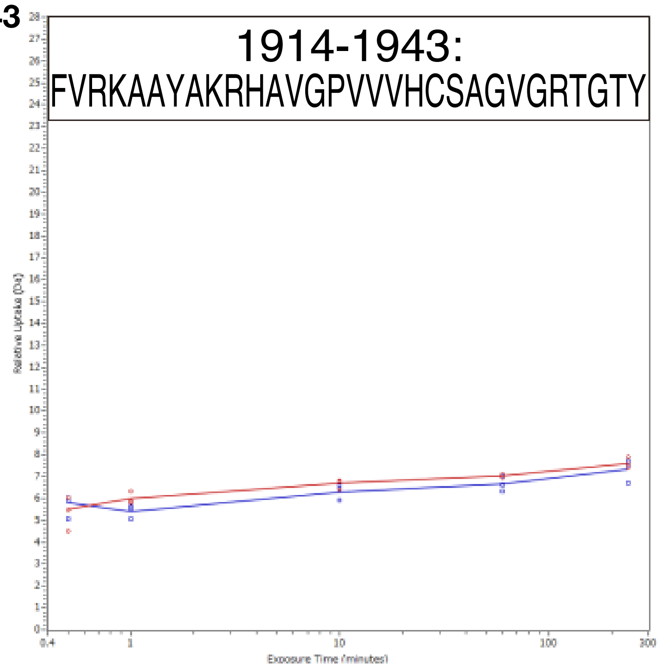

B44

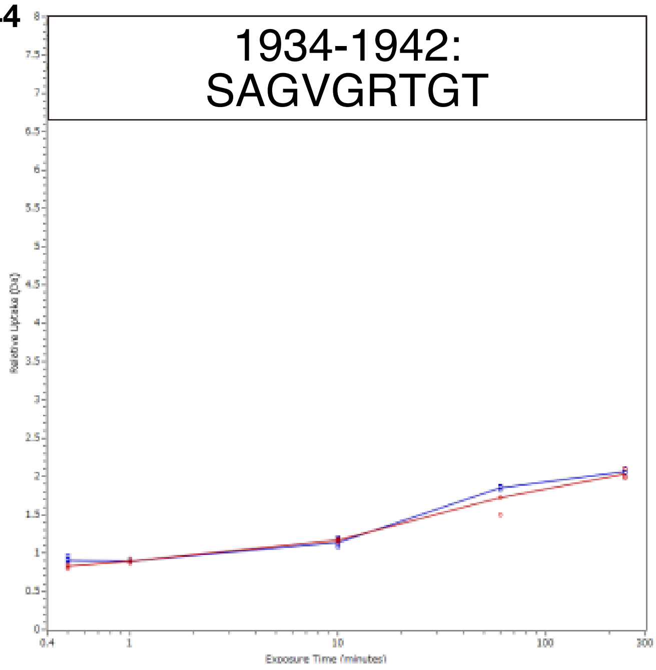

B45

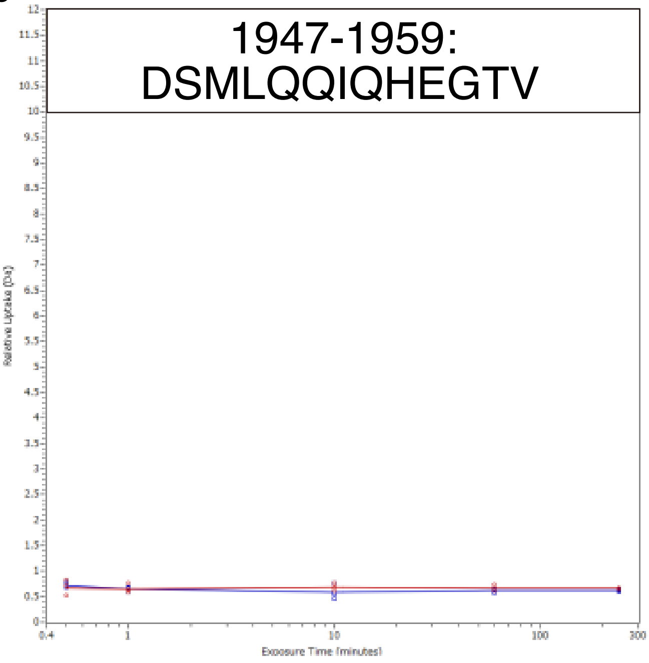

B46

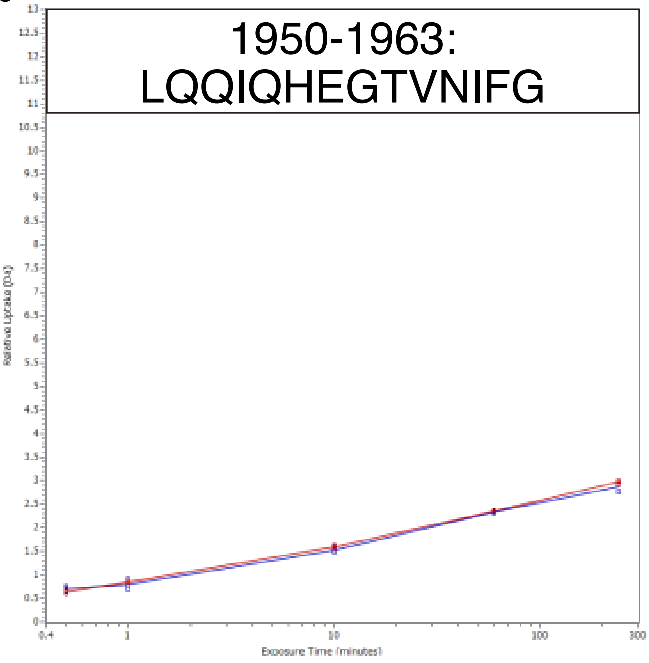

B47

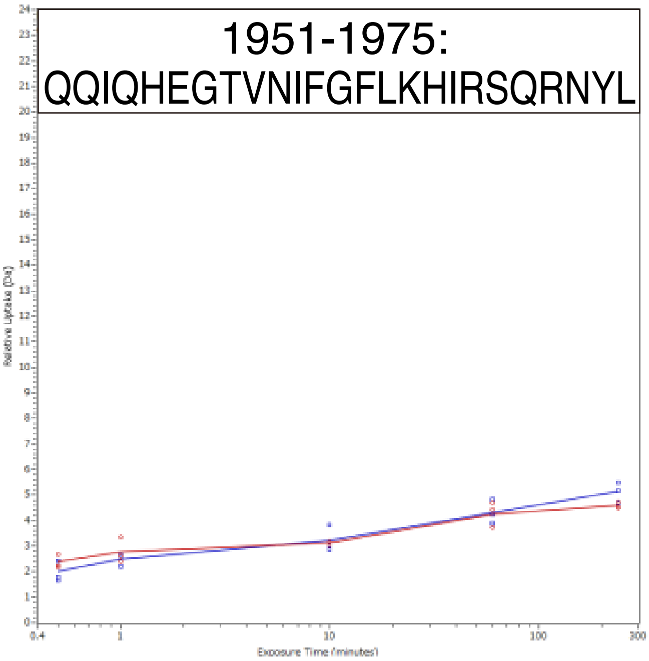

B48

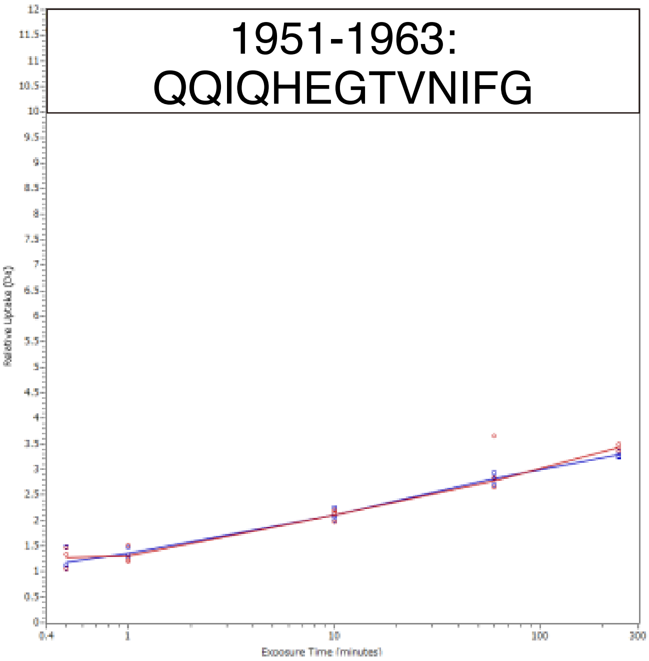

Supplementary Figure S3B

B49

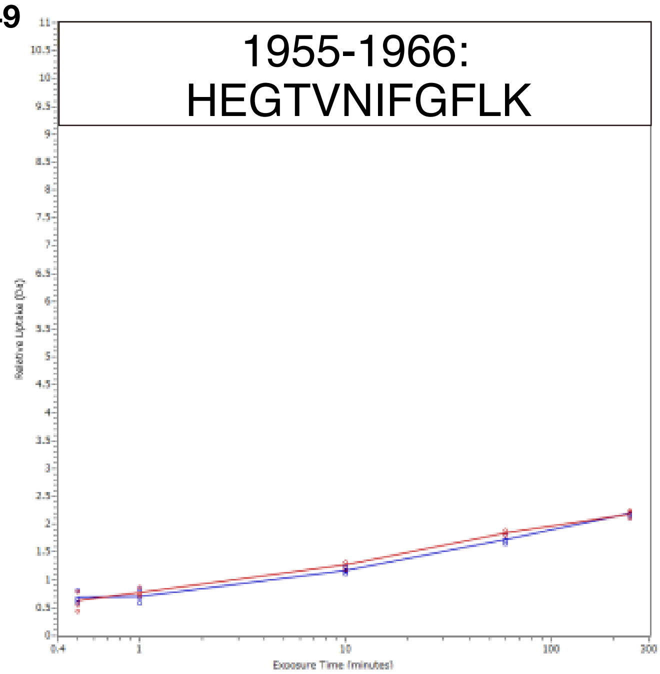

B50

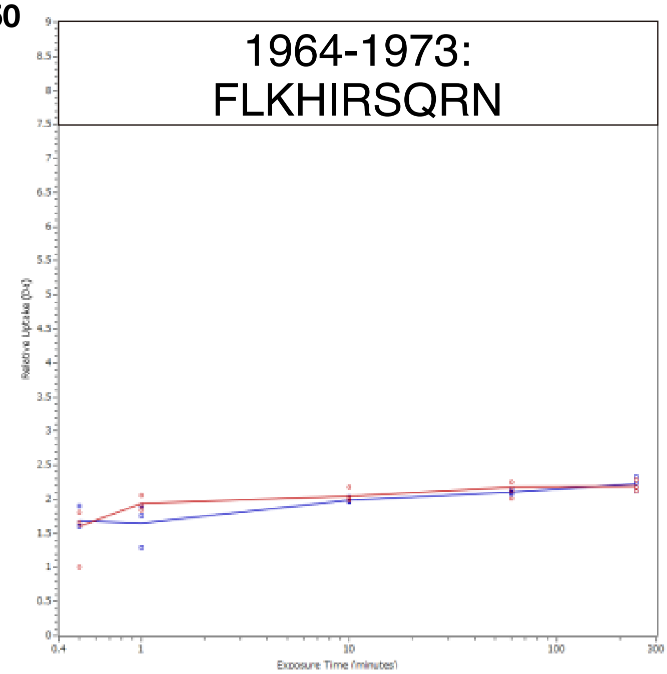

B51

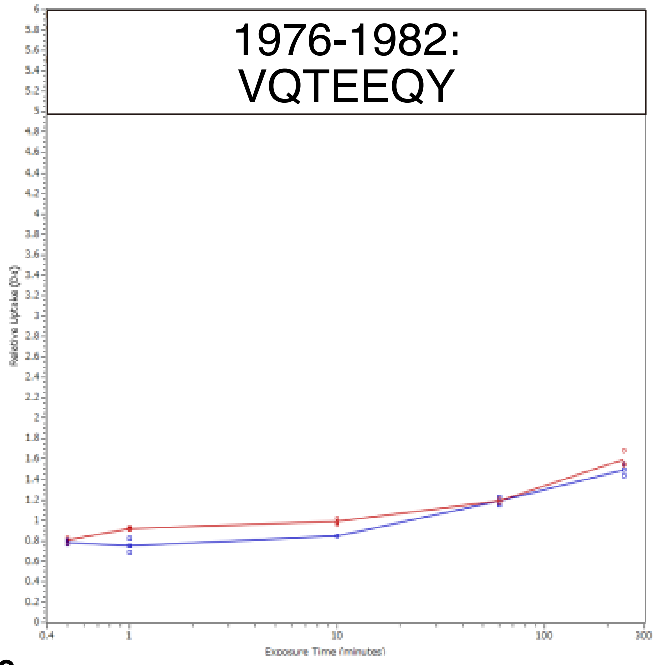

B52

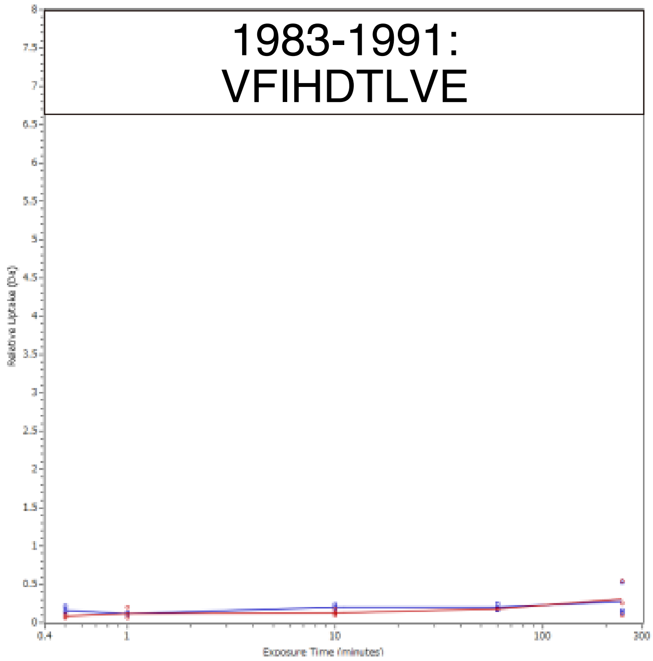

B53

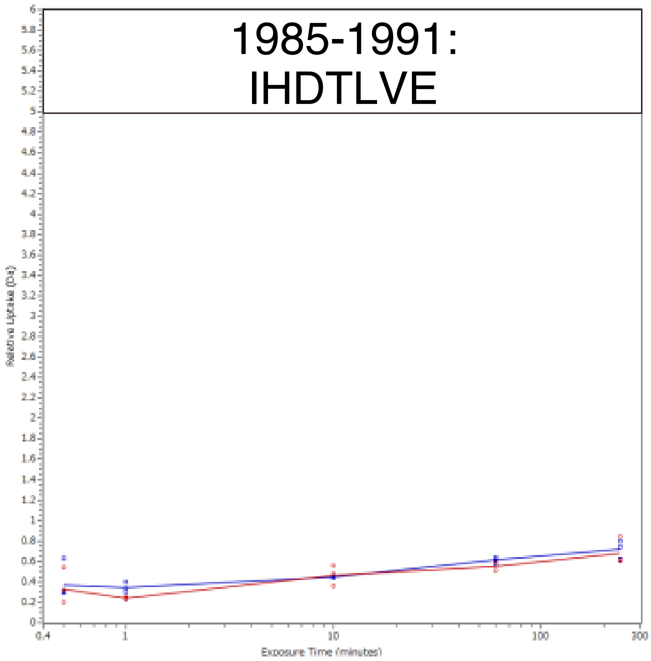

B54

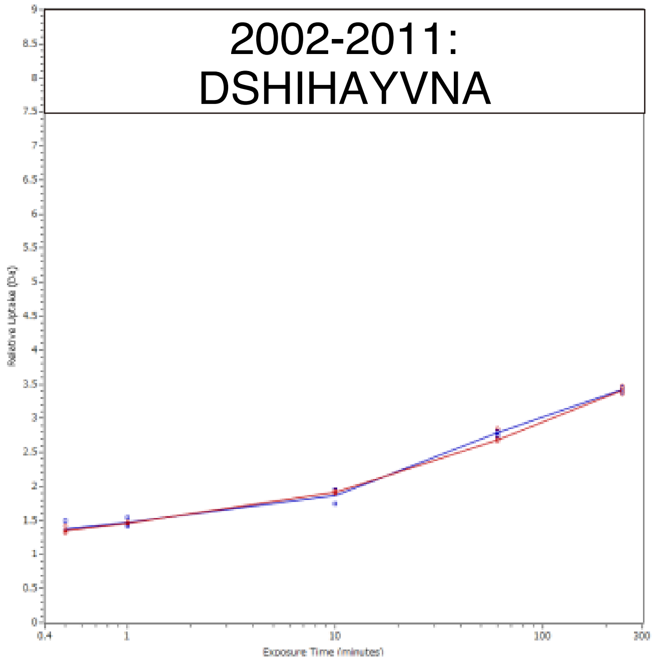

Supplementary Figure S3B

B55

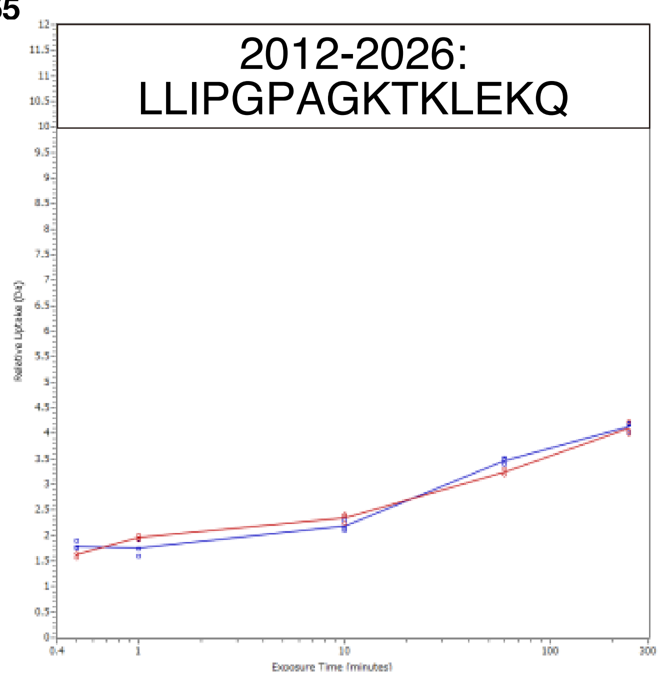

B56

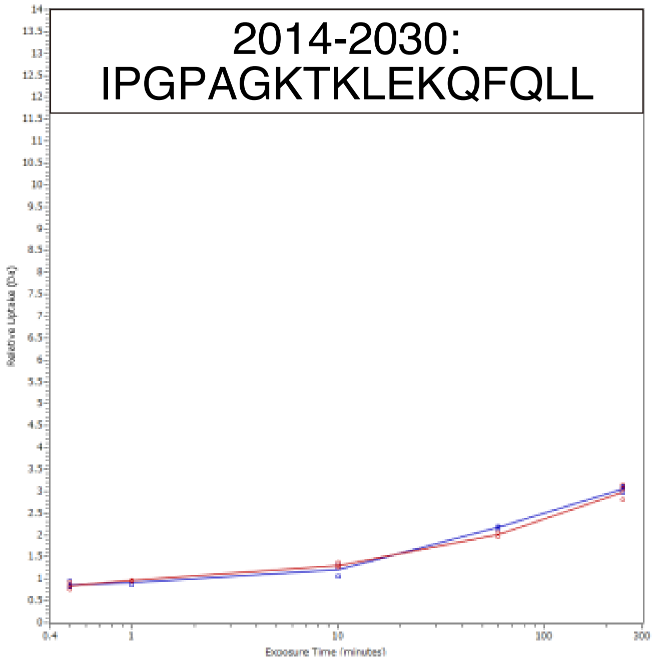

B57

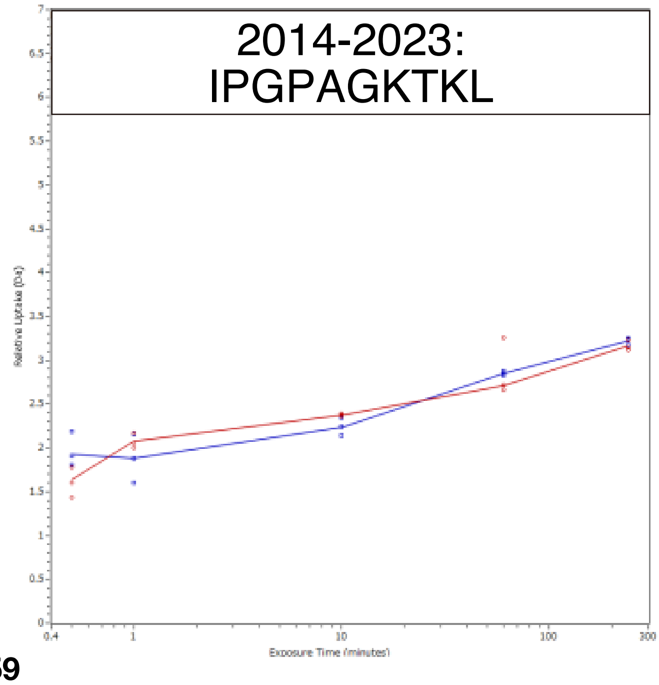

B58

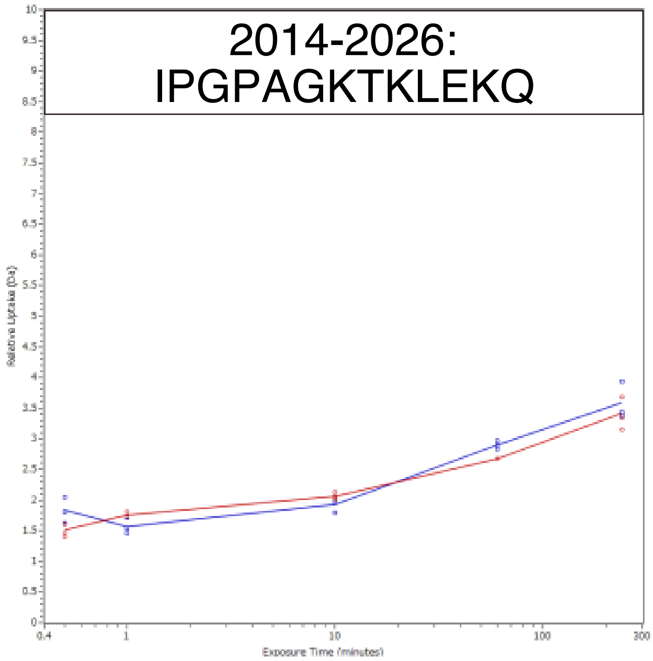

B59

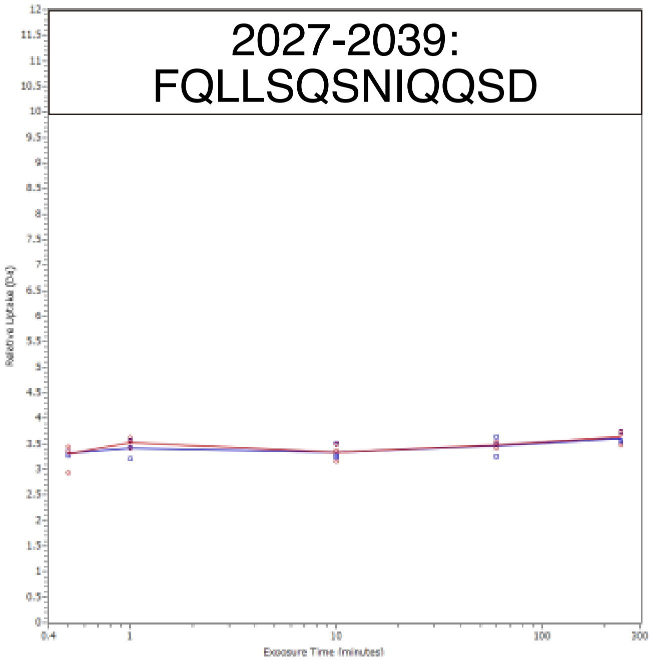

B60

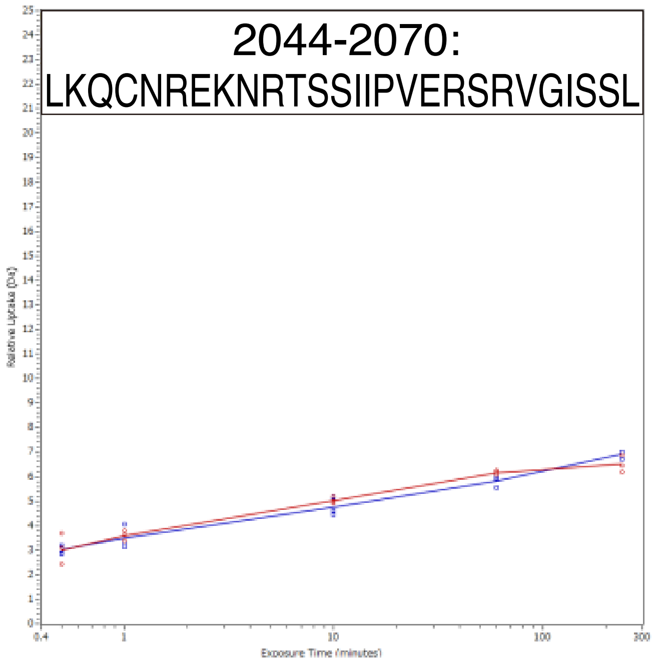

Supplementary Figure S3B

B61

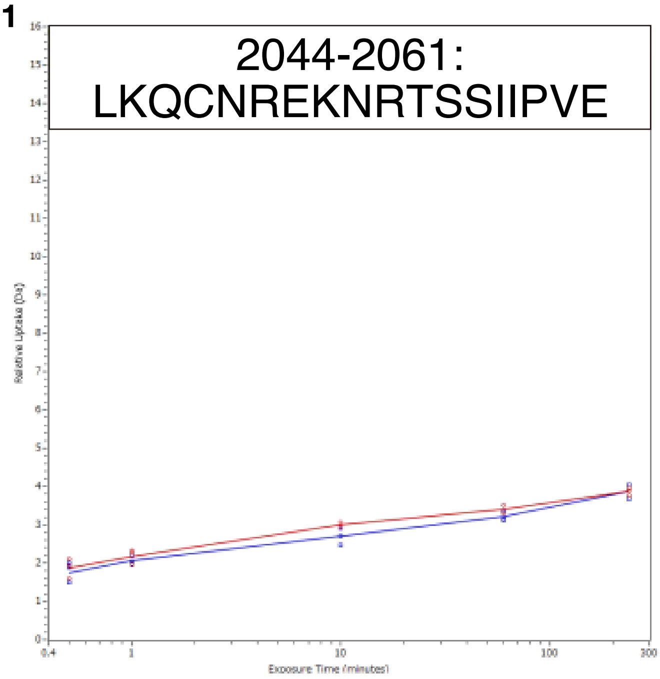

B62

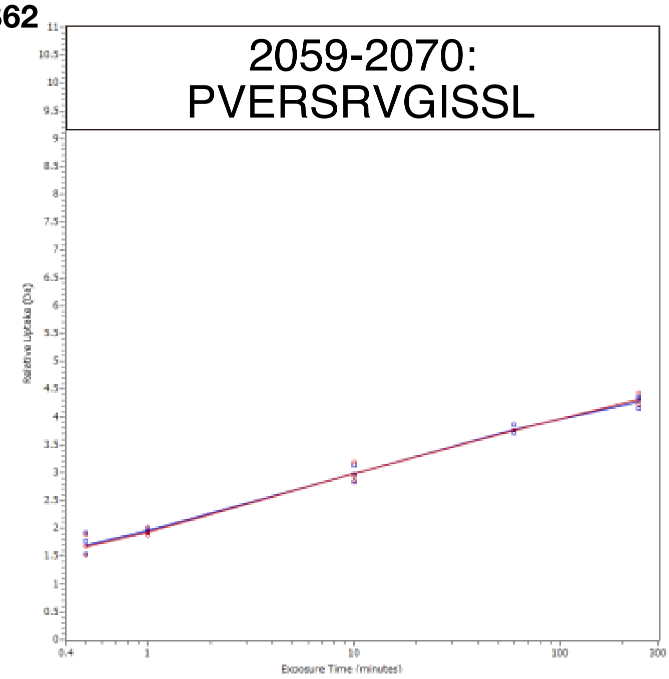

B63

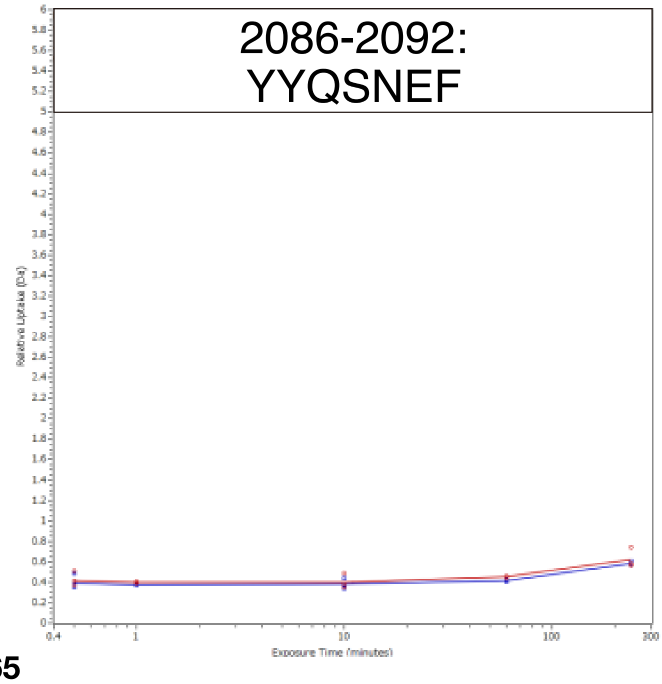

B64

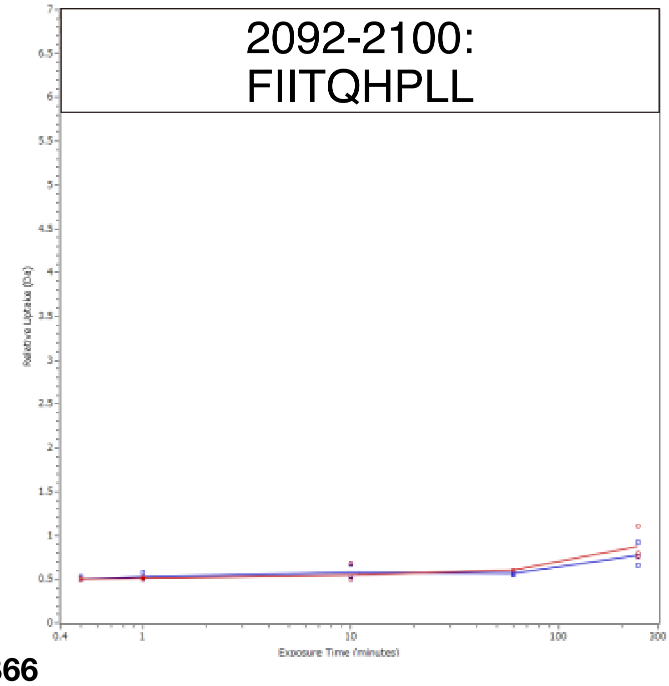

B65

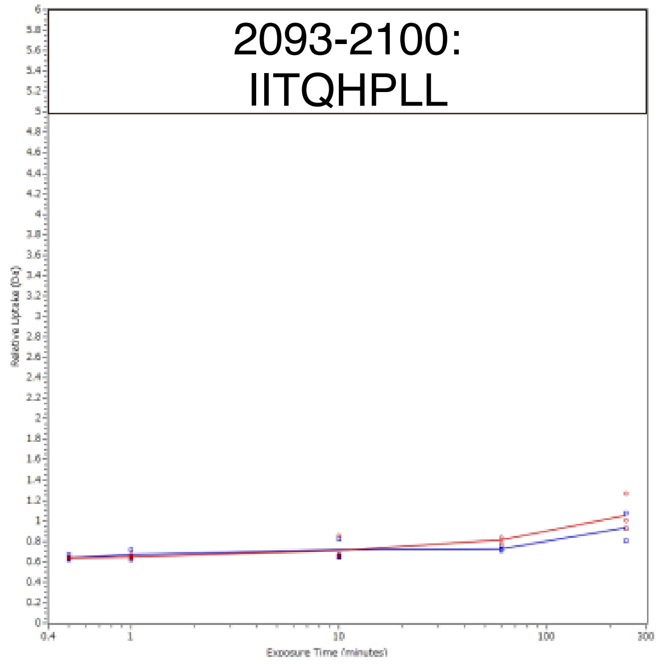

B66

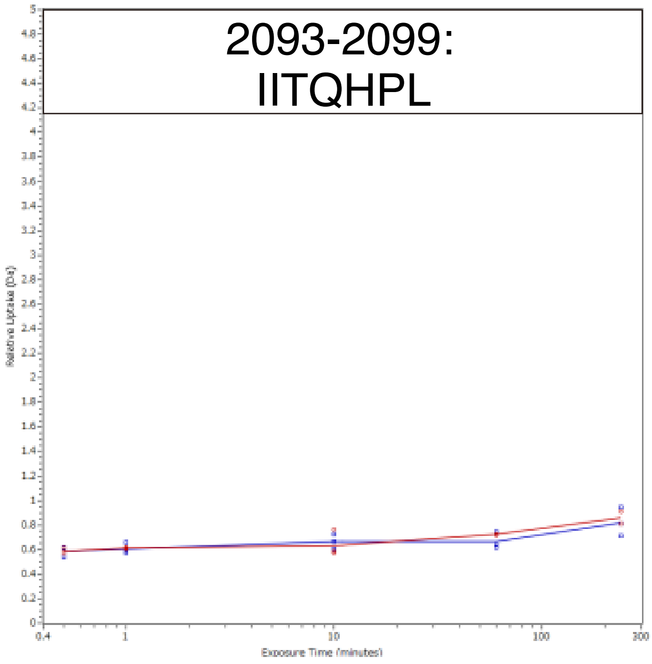

Supplementary Figure S3B

B67

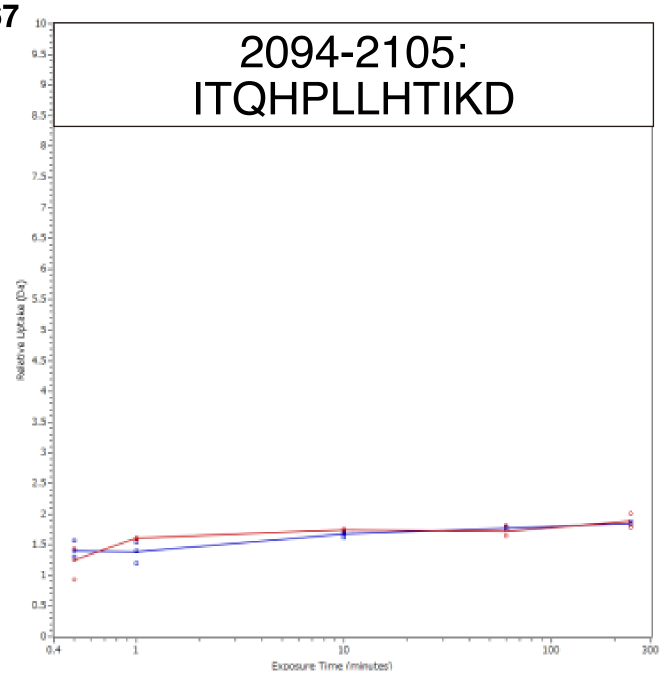

B68

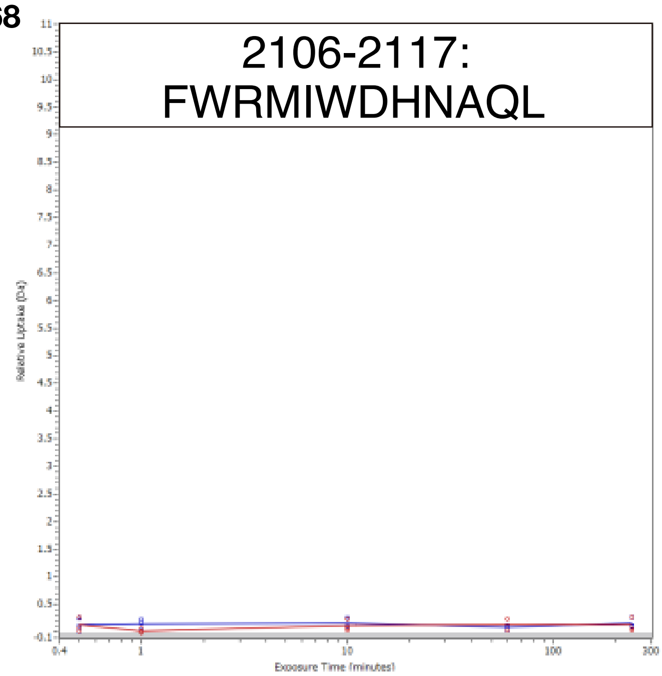

B69

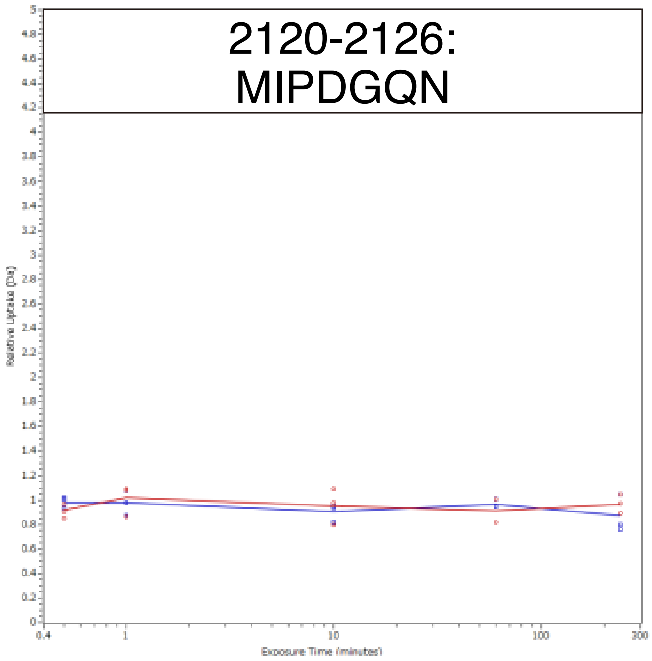

B70

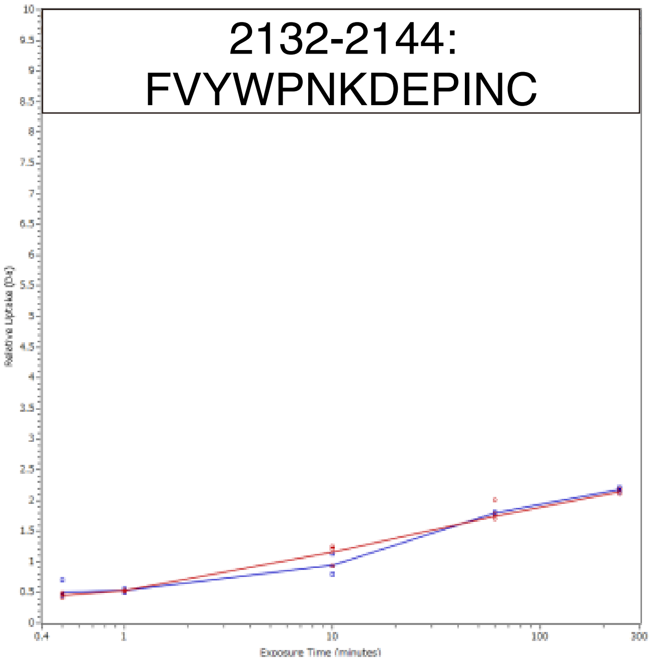

B71

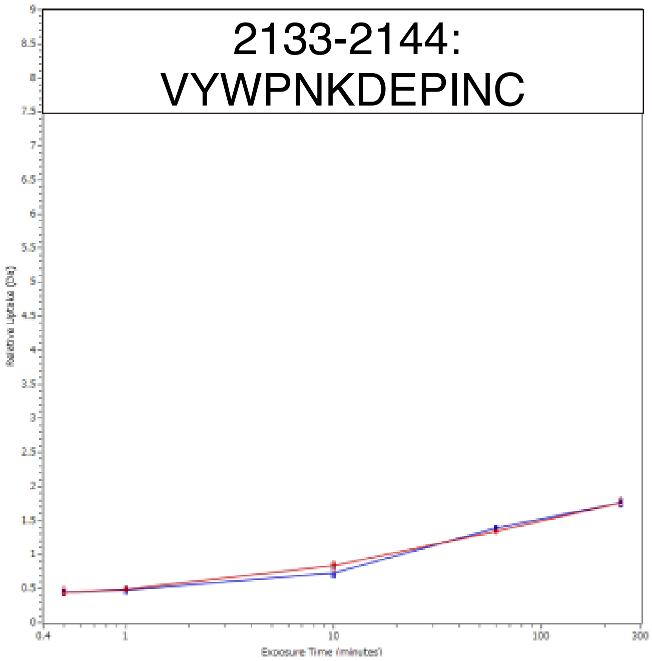

B72

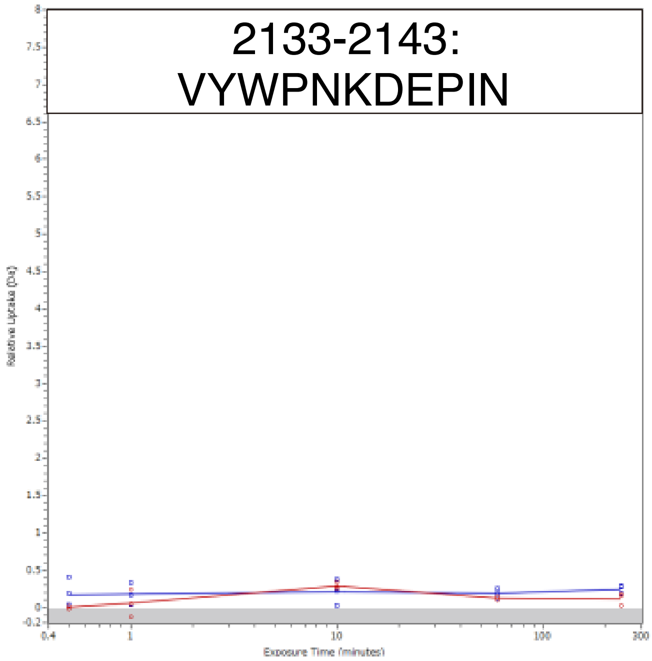

Supplementary Figure S3B

B73

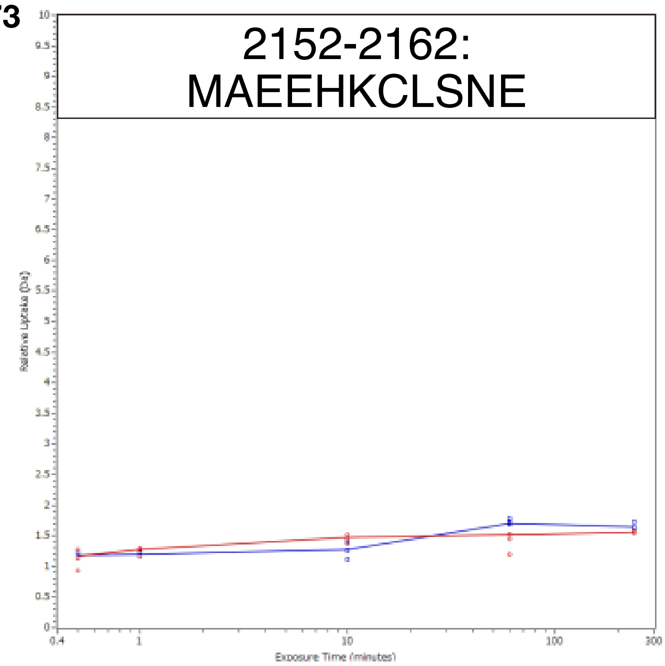

B74

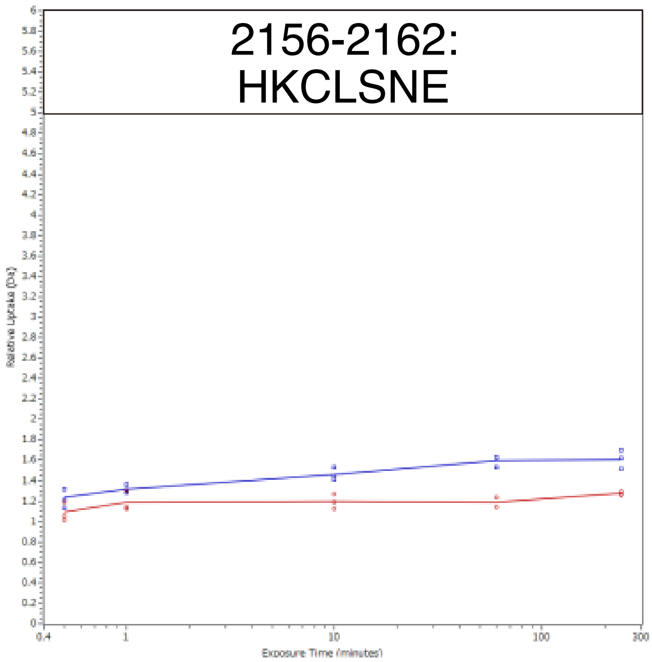

B75

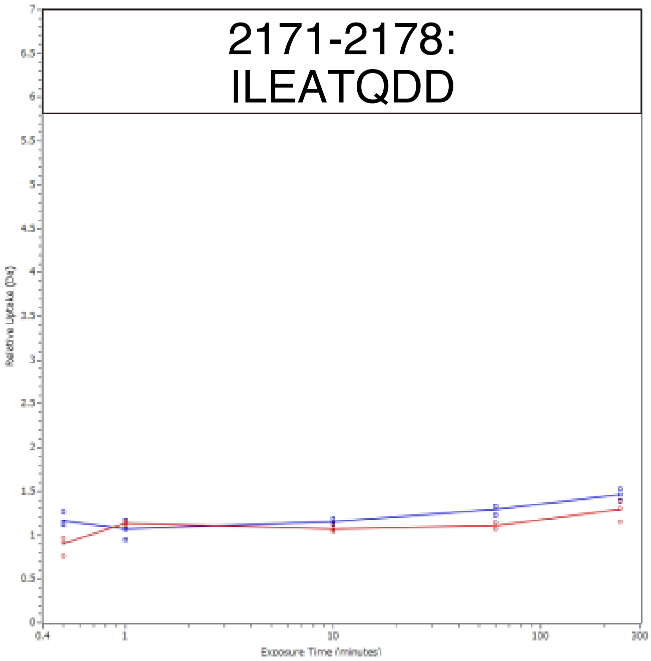

B76

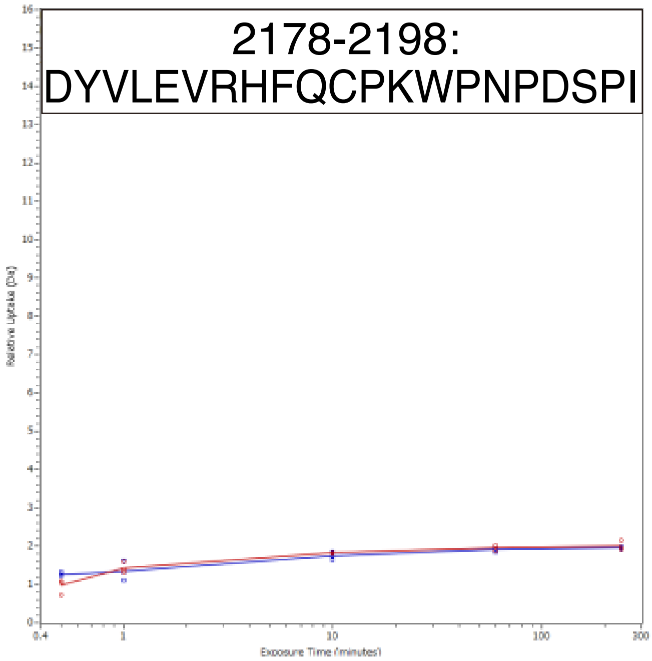

B77

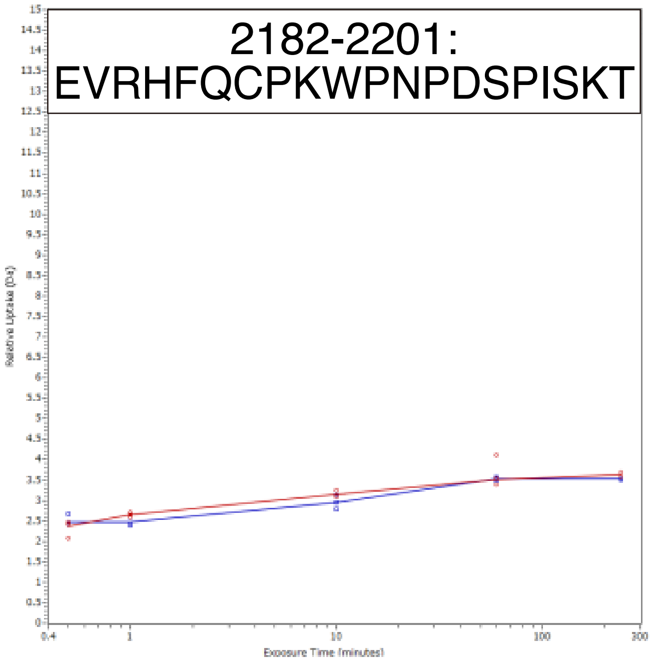

B78

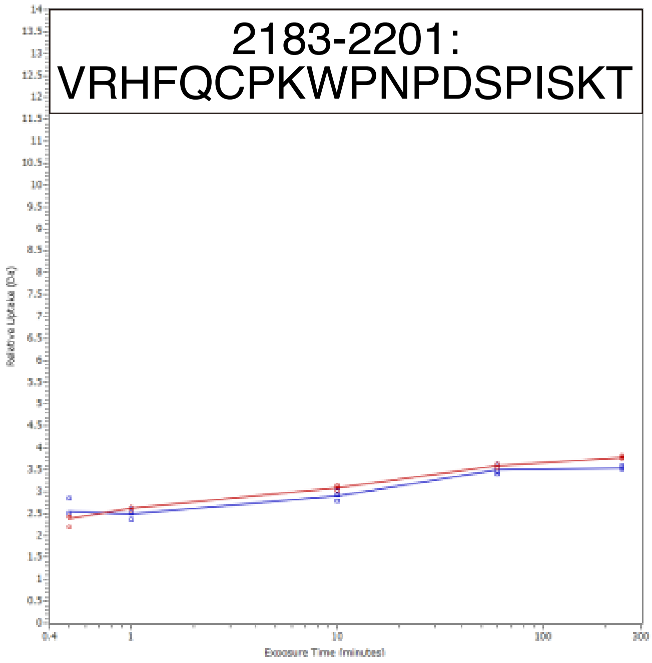

Supplementary Figure S3B

B79

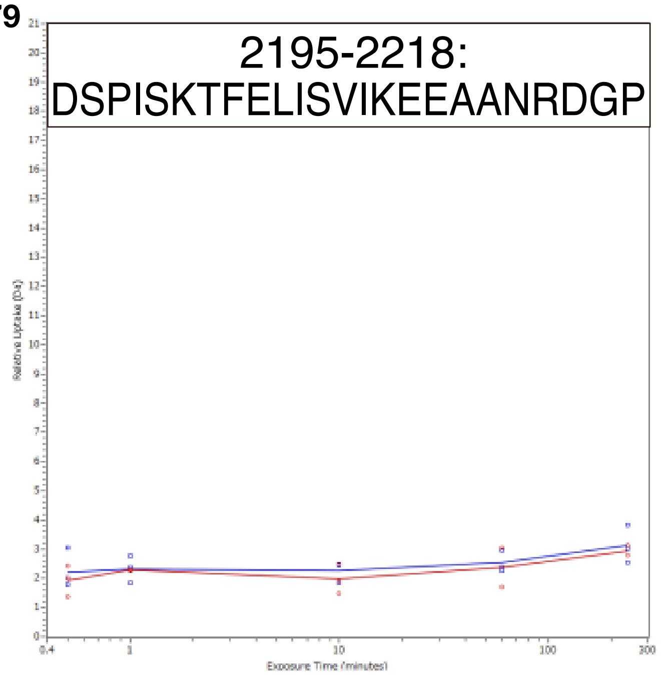

B80

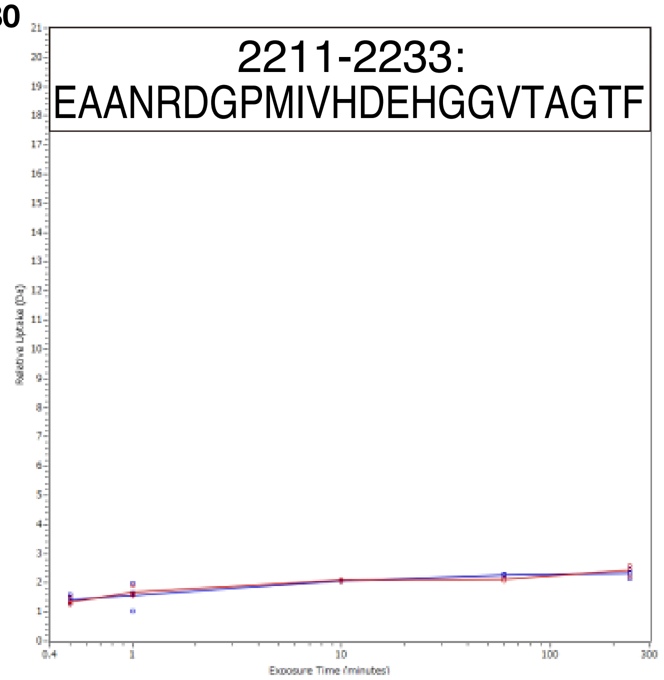

B81

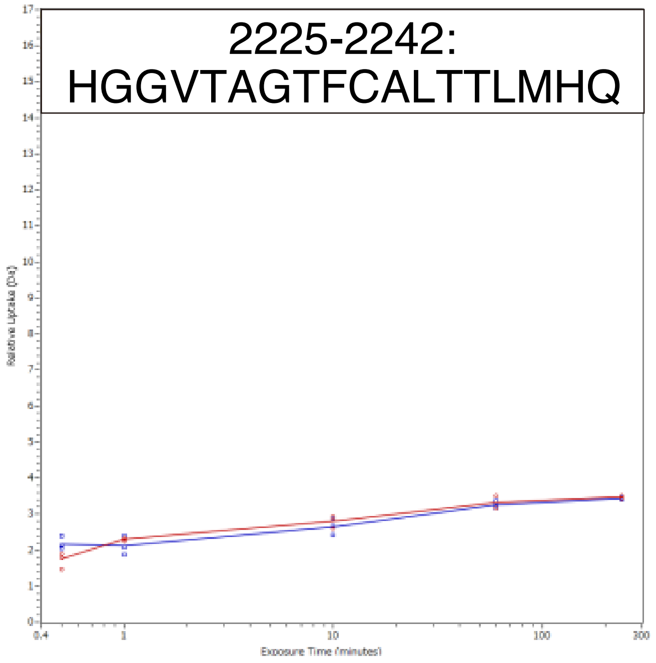

B82

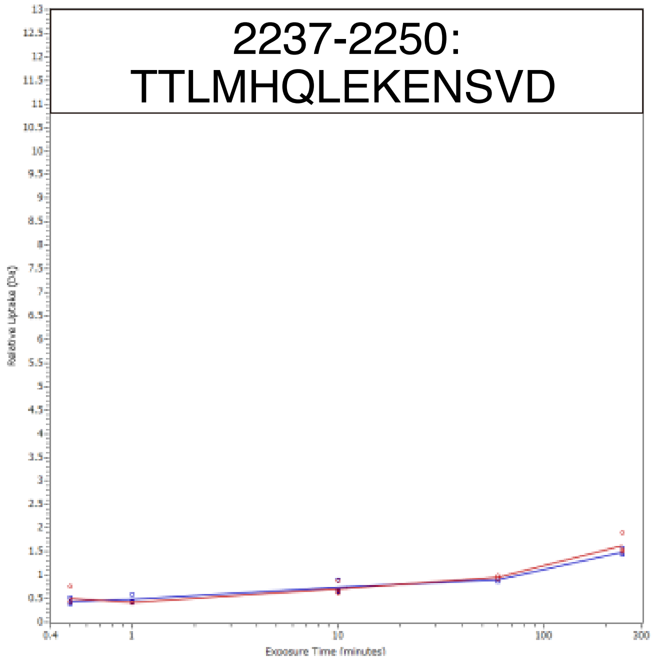

B83

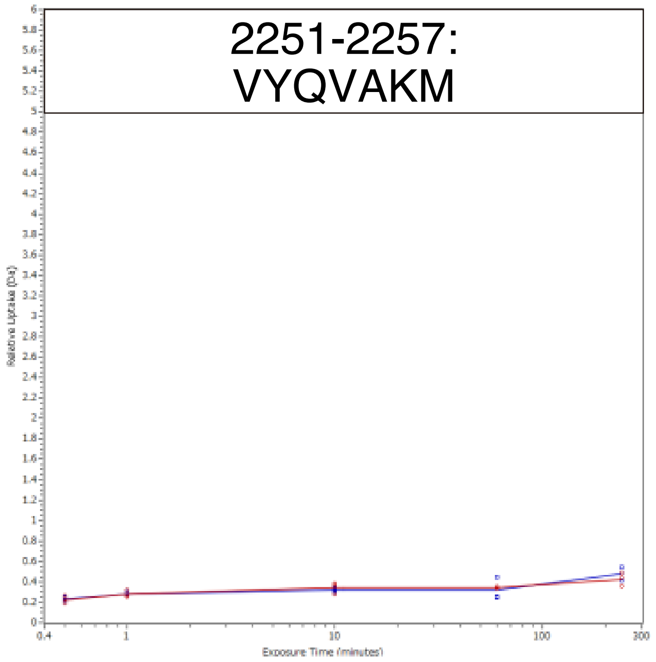

B84

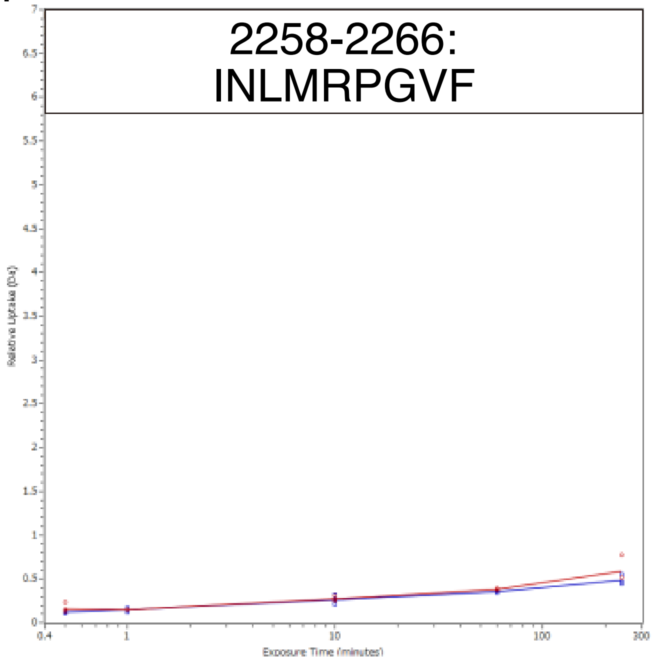

Supplementary Figure S3B

B85

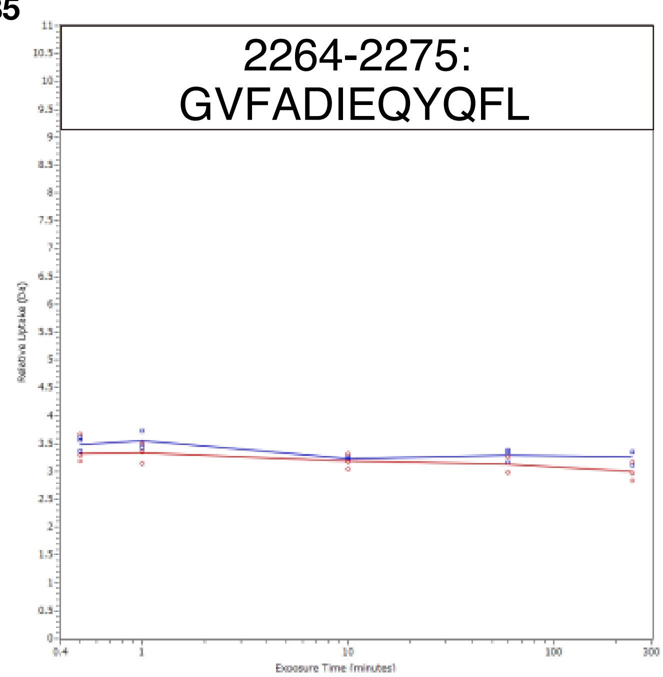

B86

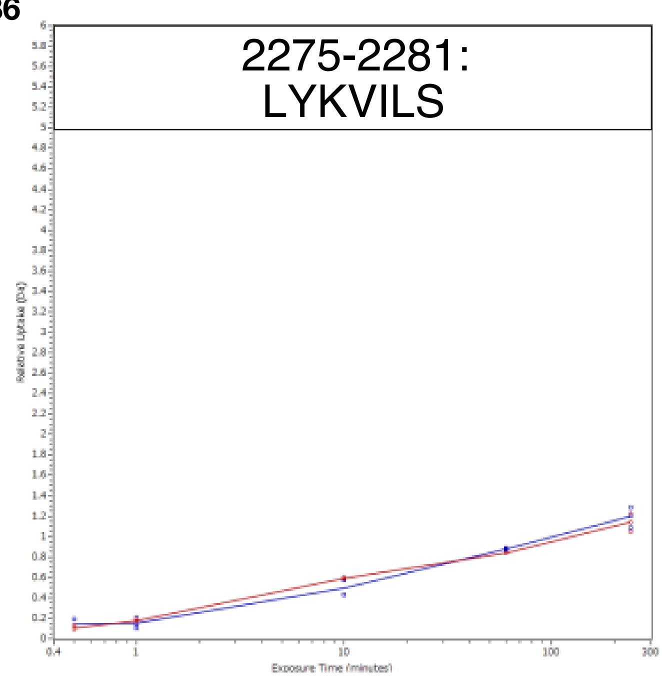

B87

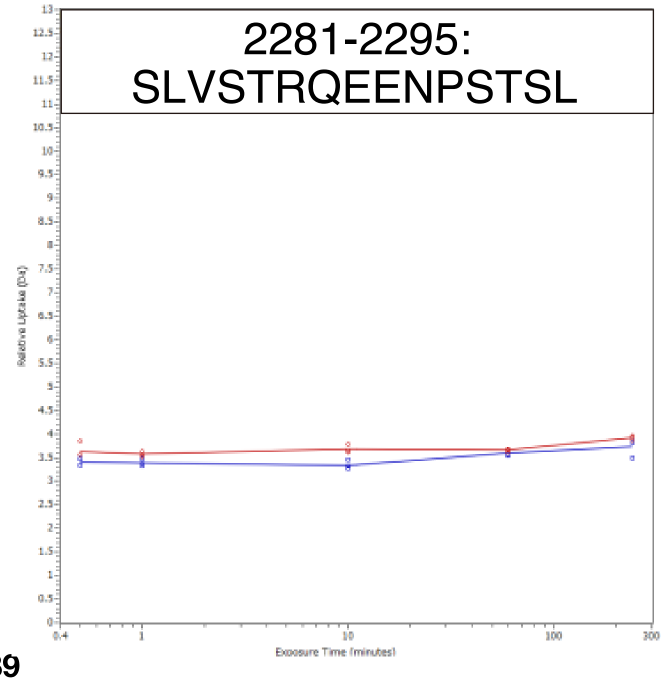

B88

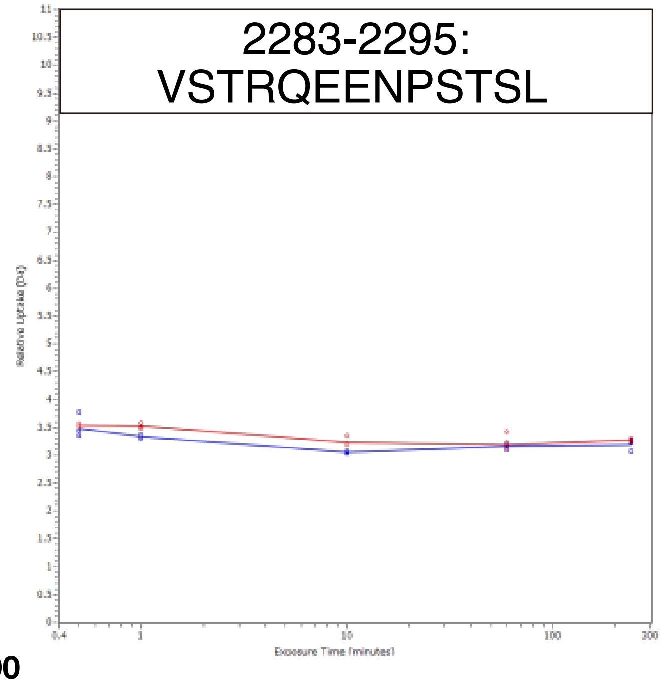

B89

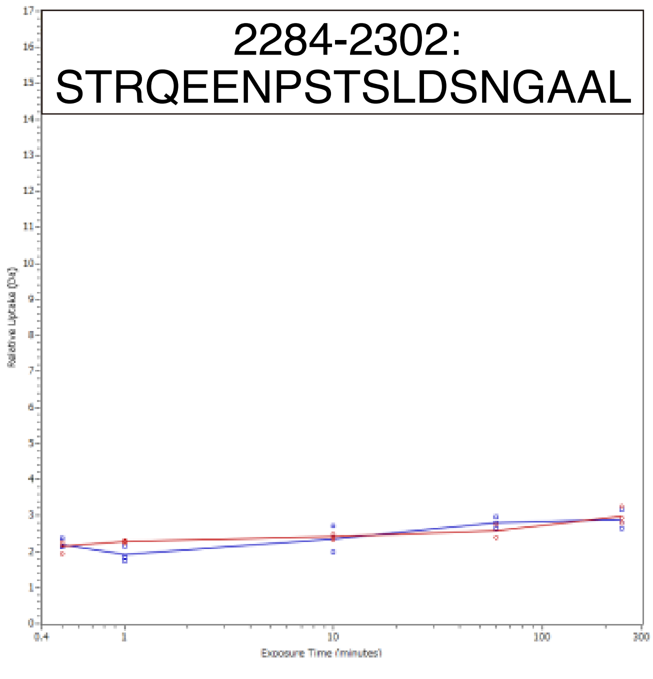

B90

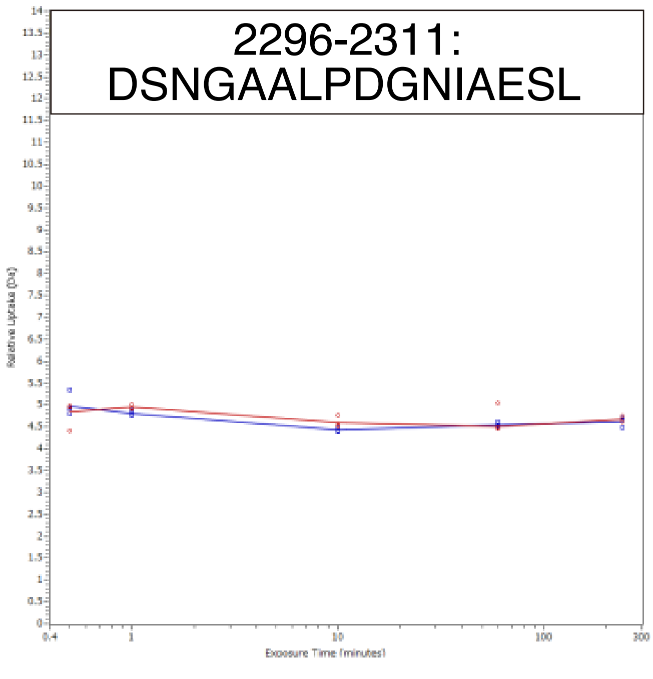

Supplementary Figure S4

A

Data collection and refinement statistics for PTPRZ-D1	
**Data Collection**	
Space group	$P2_12_12_1$
Cell Dimensions	
a,b,c (Å)	54.64, 72.25, 90.56
Resolution (Å)	1.86
R_{merge}	0.039 (0.432)
$I/\sigma I$	45.9 (3.6)
Completeness (%)	99.5 (99.4)
Redundancy	7.6 (6.4)
**Refinement**	
Resolution (Å)	1.86
No. reflections	29084
R_{work} / R_{free}	18.6 / 22.1
No. atoms	
Protein	2289
Ligand/ion	4
Water	247
B-factors	
Protein	16.392
Ligand/ion	47.912
Water	28.829
R.m.s deviations	
Bond lengths (Å)	0.021
Bond angles (°)	1.977

B

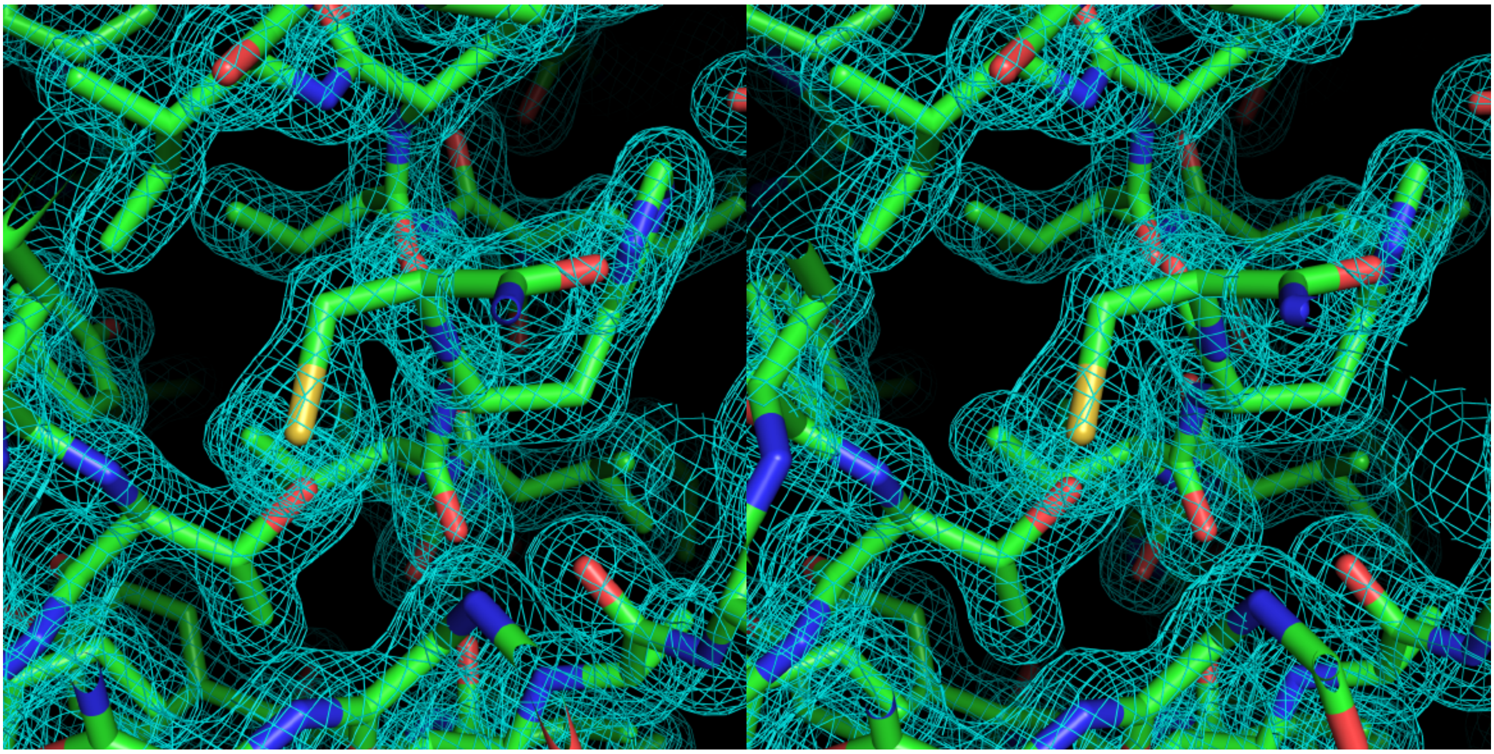

Supplementary Figure S5

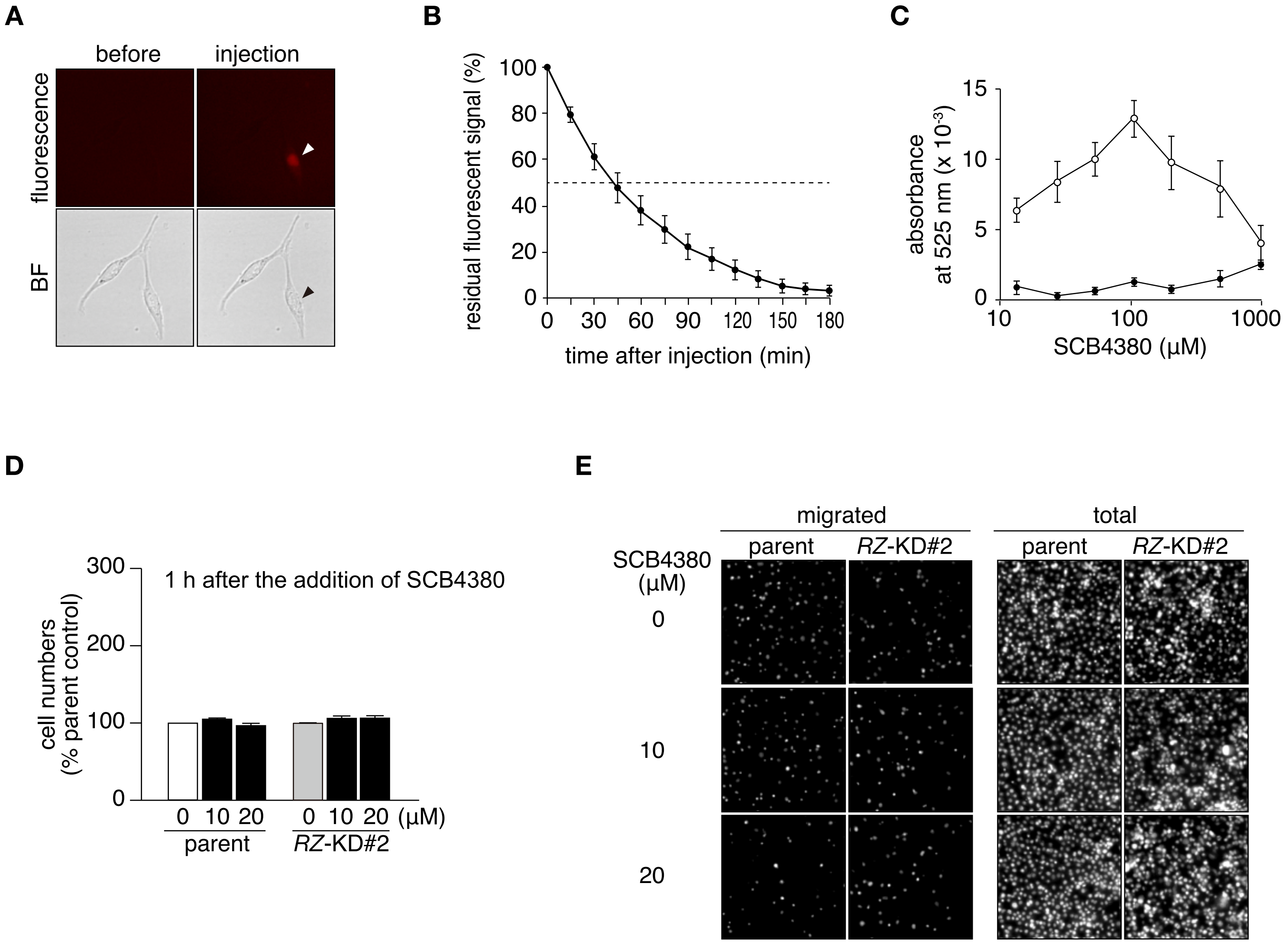

Supplementary Figure S6

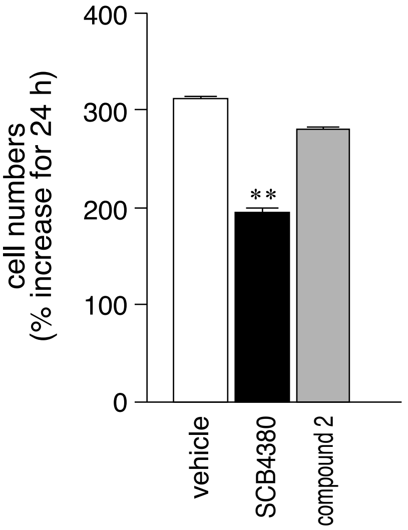

Supplement: Supplementary Information [file srep20473-s1.pdf]
